# Microwave gallium-68 radiochemistry for kinetically stable bis(thiosemicarbazone) complexes: structural investigations and cellular uptake under hypoxia†
†Electronic supplementary information (ESI) available. CCDC 1001632–1001634. For ESI and crystallographic data in CIF or other electronic format see DOI: 10.1039/c5dt02537k



**DOI:** 10.1039/c5dt02537k

**Published:** 2015-11-19

**Authors:** Israt S. Alam, Rory L. Arrowsmith, Fernando Cortezon-Tamarit, Frazer Twyman, Gabriele Kociok-Köhn, Stanley W. Botchway, Jonathan R. Dilworth, Laurence Carroll, Eric O. Aboagye, Sofia I. Pascu

**Affiliations:** a Department of Medicine , Imperial College , Du Cane Road , W12 0NN , London , UK . Email: l.carroll@imperial.ac.uk ; Email: eric.aboagye@imperial.ac.uk; b Department of Chemistry , University of Bath , Claverton Down , BA2 7AY , UK . Email: s.pascu@bath.ac.uk; c Oxford Brookes University , Faculty of Health and Life Sciences , The Science and Technology Facilities Council , Rutherford Appleton Laboratory , Harwell , Oxford , UK; d Inorganic Chemistry Laboratory , South Parks Road , Oxford OX2 6TT , UK

## Abstract

We report the microwave synthesis of several bis(thiosemicarbazones) and the rapid gallium-68 incorporation to give the corresponding metal complexes. These proved kinetically stable under ‘cold’ and ‘hot’ biological assays and were investigated using laser scanning confocal microscopy, flow cytometry and radioactive cell retention studies under normoxia and hypoxia. ^68^Ga complex retention was found to be 34% higher in hypoxic cells than in normoxic cells over 30 min, further increasing to 53% at 120 min. Our data suggests that this class of gallium complexes show hypoxia selectivity suitable for imaging in living cells and *in vivo* tests by microPET in nude athymic mice showed that they are excreted within 1 h of their administration.

## Abstract 2

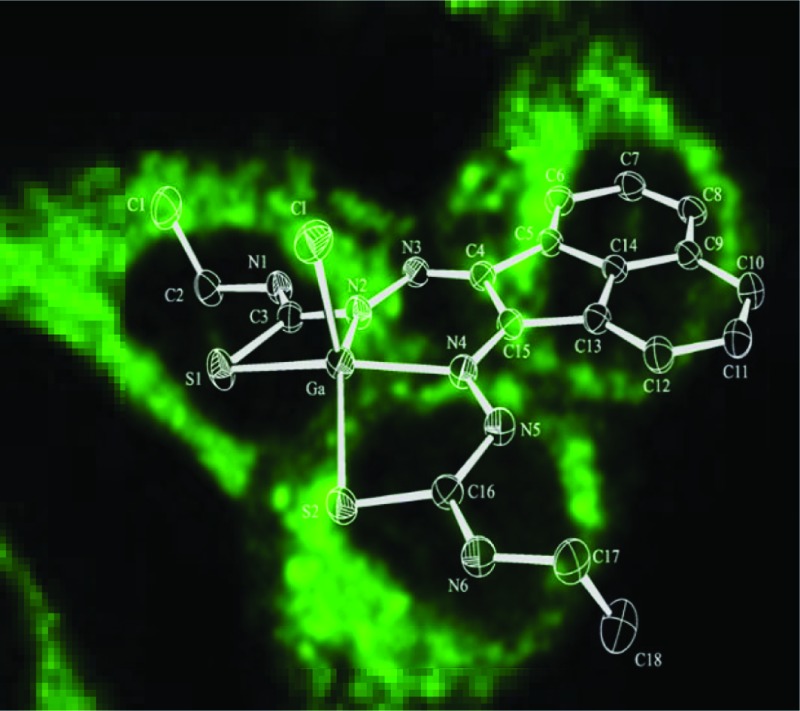
Hypoxia selectivity of new gallium-68 bis-(thiosemicarbazones) synthesised *via* microwave heating were investigated.

## Introduction

The aqueous radiochemistry of Group 13 metals has become a matter of paramount importance in the design of new imaging agents anchored onto unusual metallic radioisotopes for PET (gallium-68) or SPECT (indium-111).^1–3^ To date however, there have been relatively few gallium labelling reactions that do not consist of the well-established NOTA or DOTA core.^4^

Gallium-68 complexes have the potential to be extremely useful in clinical imaging, due to their rapid radiosynthesis and easy availability of the isotope from on-site generators (then making it cost effective and simple to use, by analogy to ^99m^Tc). Gallium-68 also has a rather short half-life (*t*_1/2_ = 68 min), preventing the exposure of the patient to any larger dose of radiation than is required for the imaging procedure.

Hypoxia is the term used to describe low oxygen concentrations present in a specific environment and has a large impact on the development and subsequent treatment of a number of disease types, including cancer. The lack of oxygen usually indicates there is poor vascularisation within a tumour and this is, additionally causing the delivery of chemotherapeutic drugs to the target to become rather challenging. A number of these agents also interact with oxygen, making their activation impossible under hypoxia.^5–7^ Effective radiotherapy also requires molecular oxygen which acts as a potent chemical radiosensitiser, thus hypoxia renders cells more resistant to treatment.^8,9^ As such, patients with hypoxic tumours have a lower survival rate, and its early identification is of great interest to the clinical community. Direct methods of diagnosis such as biopsy are highly invasive to the patient, and in recent years positron emission tomography (PET) imaging has taken the lead in the identification of hypoxic regions *in vivo* as a non-invasive imaging method.^10^

The detection of hypoxia using PET imaging has been developed over the past twenty years since the discovery of nitroimidazoles in the mid-1980s, with subsequent research moving into metal-containing complexes. The current gold standards for tumour hypoxia imaging are [^18^F]FMISO and [^64^Cu]Cu-ATSM. Despite their obvious utility, both have significant drawbacks: [^18^F]FMISO suffers from a low log *P* and no active uptake mechanism, leading to long imaging protocols to allow for sufficient uptake in the desired tissue and good signal-to-background ratios. [^64^Cu]Cu-ATSM has high liver uptake, making tumours in the lower gastrointestinal region challenging to distinguish from background. There have been recent developments elucidating some of the uncertainties with its suspected mechanism, and even with provocative clinical data demonstrating the ability of [^64^Cu]Cu-ATSM to delineate therapy “responders” from “non-responders” in multiple tumour types, its activity *in vivo* is currently under discussion within the wider imaging community.^11–18^

Along with altering the metal centre, we recently discovered that a modification of the ligand backbone to include a naphtyl group allows for an intrinsic fluorescence. Prior to our work there were only very few ligand systems which could give invaluable information on intracellular distribution that PET isotopes alone cannot provide. As this ligand system contains N/S hard/soft donor centres similar to H_2_ATSM (which has displayed hypoxia selective retention for ^64^CuATSM *in vitro* in contrast to ^64^Cu(OAc)_2_ alone, whereas both display similar hypoxia selectivity *in vivo*, hypoxia selectivity of any new complex would need to be explored.^18–21^

**Fig. 1:**
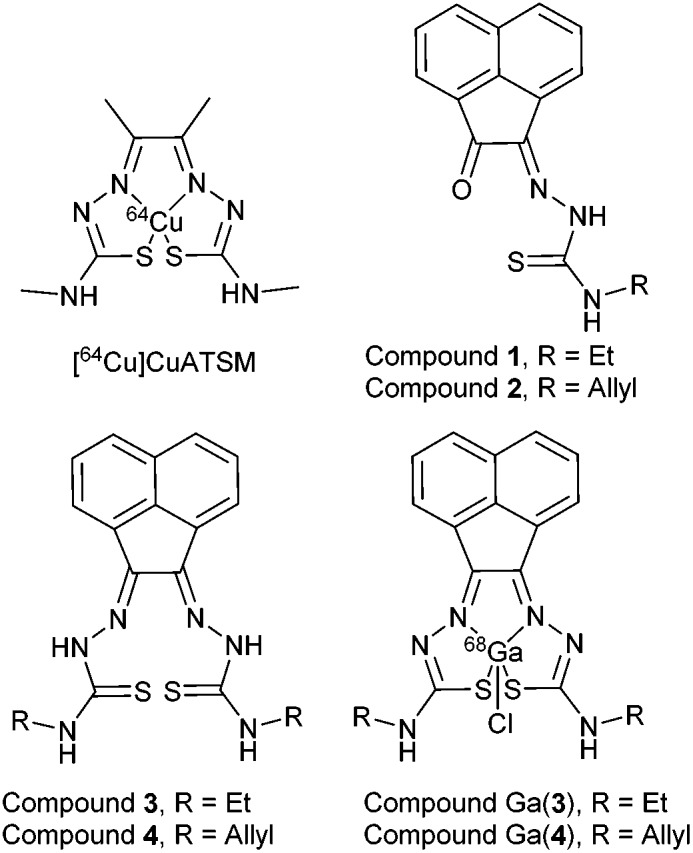
Schematic representative structure of [^64^Cu]Cu-ATSM and the structure of mono-substituted and bis-substituted ligand precursors to the target [^68^Ga]Ga-BTSC complexes studied herein.

Despite the differing coordination chemistry possessed by gallium(iii) *vs.* copper(ii), we propose that a bis(thiosemicarbazonato) complex is an attractive target in allowing access to new kinetically stable bifunctional gallium-chelators (Fig. 1) and their radiolabelled analogues in this family.

## Results and discussion

### Microwave-assisted synthesis

**Scheme 1:**
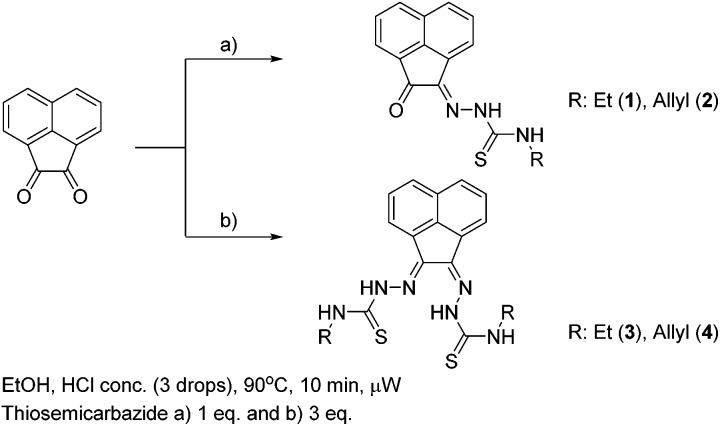
Synthesis of mono and bis(thiosemicarbazones) *via* microwave assisted heating.

The synthesis of mono and bis(thiosemicarbazones) using microwave assisted heating conditions was investigated (Scheme 1). Despite being a common method in organic synthesis, particularly involving nucleophilic substitution reactions, microwave synthesis has been little used in the synthesis of thiosemicarbazones and not applied in the synthesis of acenaphthenequinone thiosemicarbazones and their radiochemistry before.^22,23^

Compounds **1–4** (as well as their Me-functionalised derivatives, see ESI†) were successfully obtained by applying microwave assisted reaction conditions. In comparison with the conventional heating procedure, this rapid method led to formation of thiosemicarbazones in comparable yields for the case of the mono(thiosemicarbazone) ligands **1** and **2** and with significantly higher yields in the case of bis(thiosemicarbazones) **3** and **4**. Also, the reaction time was reduced from 3–4 h to just under 10 min (see information in ESI†). The applicability of microwave heating in the synthesis of mono and bis(thiosemicarbazones) has been demonstrated and should be the method of choice in future such conversions. Furthermore, the cyclisation of thiosemicarbazide starting materials, a process which occurred frequently as a side-reaction upon prolonged heating (which limited the bis(thiosemicarbazonate) ligand and complexes formation yield and caused difficulties in purification) did not occur under the microwave synthesis. The transmetallation reaction from cold Zn(**3**) and Zn(**4**) to their gallium equivalents was also performed under microwave conditions, but in this case the overall yields did not seem particularly improved with respect to conventional heating. However, the reaction time was vastly improved from several hours heating to just under 10 min. The optimisation of the reaction conditions in the synthesis of the corresponding gallium complexes by microwave remain under investigation (Table 1).

**Table 1:** Reaction conditions for the μW synthesis of ligands 1–4

	Conventional synthesis			Microwave synthesis		
Comp.	*T*/°C	*t*/min	Yield	*T*/°C	*t*/min	Yield
1	78	180	85%	90	9	73%
2			63%			77%
3			85%			90%
4			74%			94%

### Solution and solid-state characterisation of ‘cold’ ligands and complexes

**Fig. 2:**
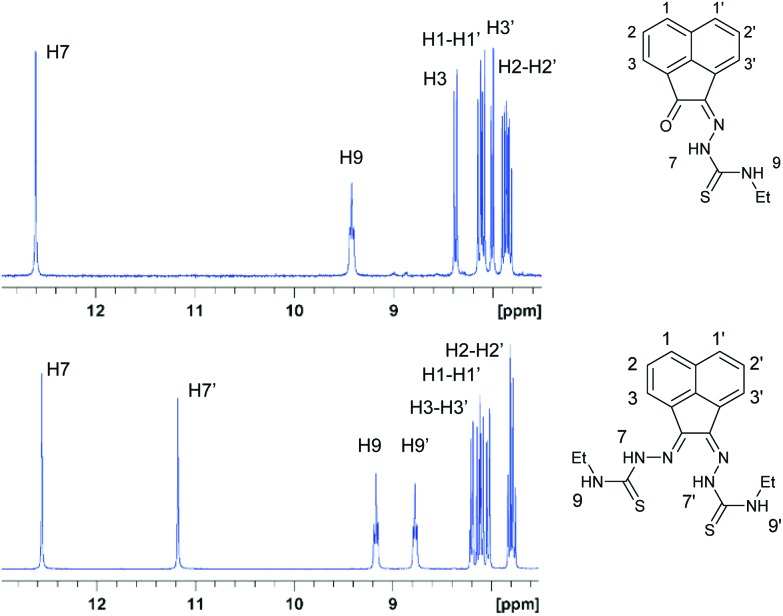
^1^H NMR spectroscopy of the aromatic and amino N*H* shifts of compounds **1** and **3** in the 7.5 to 13 ppm region.

^1^H-NMR spectroscopy indicated the high purity of the ligand precursors and that the bis(substituted) compounds showed an asymmetric conformation. Fig. 2 shows the ^1^H-NMR spectra of proligands **1** and **3** in *d*_6_-DMSO. Distinctive nitrogen-bonded protons (referred to as N*H*) for both mono(substituted) and bis(substituted) ligand precursors appear between 8.8 and 12.9 ppm. The (*E*,*Z*) N*H* resonances can be assigned accurately for the bis(substituted) ligand precursor, due to the characteristic resonances corresponding to the mono(substituted) proligand, which has been determined crystallographically to represent the *Z* geometry. In the case of compound **3** and **4** (R = allyl) bis(substituted) proligand resonances analogous to the mono(substituted) ligand precursor for between 12.5 and 12.7 ppm are representative of the *Z* hydrazone proton, with shifts between 9.1 and 9.4 ppm corresponding to external *Z* proton. This indicates that shifts of the *E* nitrogen-bound hydrogen atoms are upfield of their *Z* counterparts, with resonances between 11.2 and 11.3 ppm (hydrazonal) and 8.8 and 9.1 ppm (external).

To gain an ultimate proof into the precise nature of the synthesised species, we performed the X-ray structure determination of the ligands and complexes isolated from the parallel ‘cold’ synthesis.

**Fig. 3:**
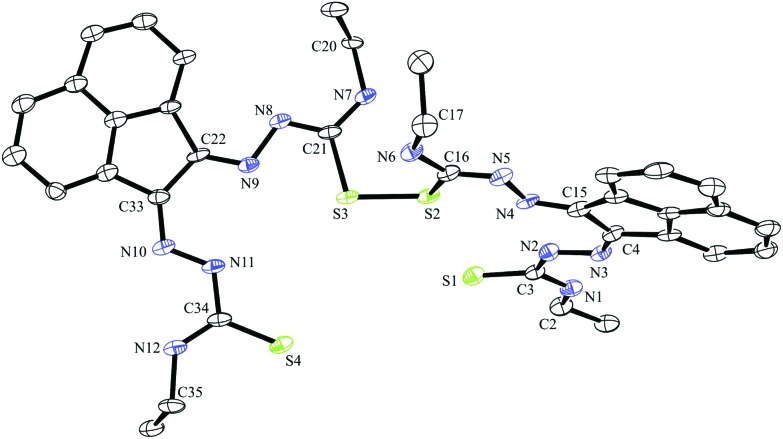
ORTEP representations at 50% probability of dimer of ethyl-substituted bis(thiosemicarbazonato) ligand compound **3**.

**Fig. 4:**
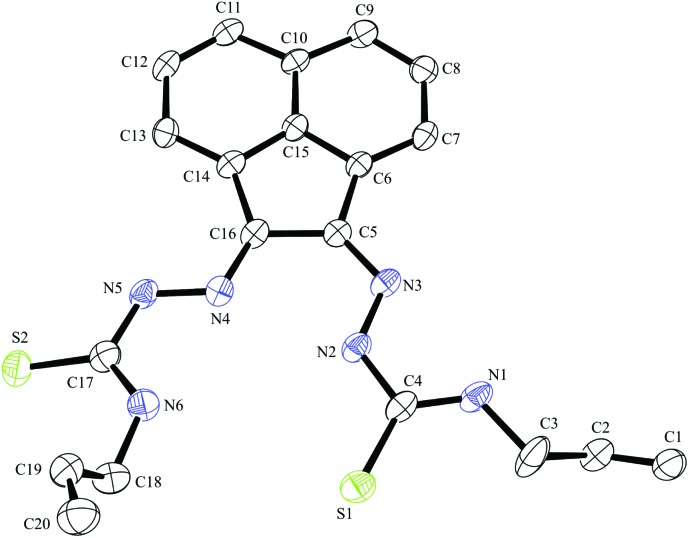
ORTEP representations at 50% probability of allyl-functionalised bis(thiosemicarbazonato) ligand compound **4**.

Crystals of gallium complex **3**, bis(thiosemicarbazonato) ligand precursor **4** and of a sulphur–sulphur dimerised form of compound **3** suitable for X-ray crystallographic analysis were grown from DMSO solutions (Fig. 2–4). The proligand **4** was found to be the unsymmetric (*E*,*Z*) isomer unlike several other related known compounds incorporating C=N bonds such as bis(alklimino)acenaphthene (alkyl-BIAN) precursors, where the (*E*,*E*) geometry was observed. Cowley *et al.* had reported (*E*,*Z*) alkyl-BIAN proligand isomers, suggesting that the observed geometry was due to the substituents steric effects, imino-nitrogen lone pair repulsion and crystal packing, which could explain the isomerism displayed by compounds Ga(**3**) and Ga(**4**).^24^ Structures reported by Besenyei *et al.* also showed that aryl(BIAN) derivatives may be observed as either (*E*,*E*) (major isomer) or (*E*,*Z*) isomers depending on the polarity of the solvent.^25^ Furthermore, Dilworth *et al.* reported a benzil bis(phenylthiosemicarbazone) ligand precursor and related proligand, which display an (*E*,*E*) and a (*Z*,*Z*) geometry respectively.^26^

**Fig. 5:**
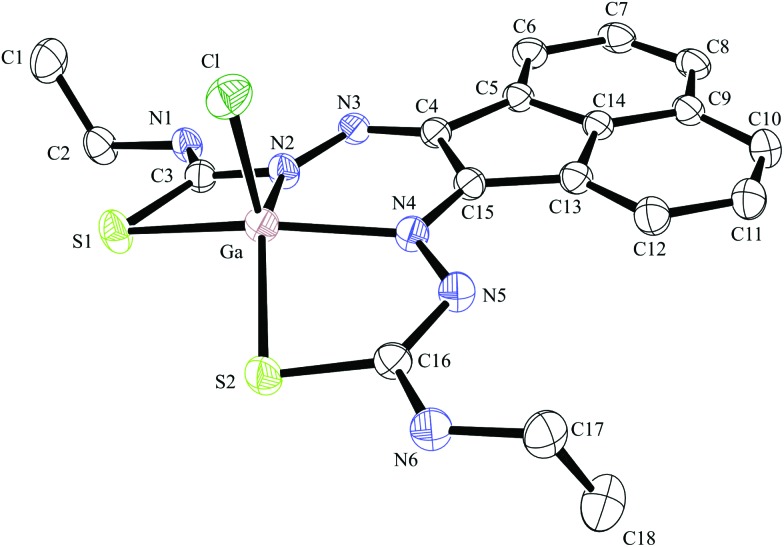
ORTEP representation of Ga(3), ellipsoids drawn at 50% probability; note hydrogen atoms were omitted for clarity.

A dimerised structure of the ethyl-functionalised bis(substituted) ligand precursor has been formed *via* sulphur–sulphur bridging: single crystals occurred from aqueous DMSO solutions over a prolonged period, and structure was elucidated by X-ray crystallography. The capacity for DMSO to favour the formation of disulphide bonds in peptides containing cysteine is well-known.^27^ Previously reported thiosemicarbazone disulphide-bridged ligand precursors were formed by oxidation *via* a metal-based catalyst. It has been well documented that Cu(ii) and Fe(iii) oxidise organo-thiols to produce sulphur–sulphur bonds.^18^ Cu(ii)- and Mn(ii)-catalysed thiosemicarbazone sulphur-bridged proligand were reported by López-Torres *et al.* and Bermejo *et al.* respectively (Fig. 3).^28,29^ The S–S bond lengths of these published ligand precursors were correspondingly 2.034(11) Å and 2.039(1) Å, which is somewhat shorter than the disulphide bond of the compound **3** dimer, 2.051(2) Å. This suggests that the proligand **3** dimer bond may be labile and the dimer formation is reversible. This is not unexpected on basis of interchange onto thiol disulphide dynamic combinatorial chemistry and related dynamic exchange (Fig. 5).^30^

Compound Ga(**4**) showed similar bond lengths and angles to those found in a methyl-functionalised mono(thiosemicarbazonato) ligand precursor reported previously by Pascu *et al.* ^31^ Interestingly, the coordination mode of the ligands were found to be asymmetric in the solid state structures of both Ga(**3**) and Ga(**4**). All complexes display a distorted square pyramidal structure around the metal centre with the chlorine atom in the axial position with the base formed by two nitrogen atoms and two sulphur atoms.

All bond lengths and angles of the ligands within Ga(**3**) and Ga(**4**) complexes are close to those determined for their reported Zn(ii) precursors. It is likely that that the rigid backbone and tight control of the ligand bite angles by the presence of the aromatic naphthyl substituents in complexes Ga(**3**) and Ga(**4**) may account for the significantly higher kinetic stability of aromatic gallium complexes with respect to aliphatic analogues. This trend is consistent with what was found previously for the aromatic Cu(ii) analogues.^28,29,32,33^

### Optical spectroscopy and cellular imaging with ‘cold’ gallium complexes

The naphthyl backbone of the ligand precursors provides electron-rich π-bonds and facilitates fluorescence emission. Therefore, fluorescence and UV-vis spectroscopies and confocal and epi-fluorescence imaging techniques allowed the monitoring of the fluorescent compounds in solution and biologically in living cells. This intrinsic fluorescence of the ‘cold’ species bypassed the necessity of conjugating the metal complexes with commercially available fluorophores, such as fluorescein or BODIPY, as we found that such conjugates are often difficult to synthesise and significantly affect the biological properties of the compound under investigation.

Appropriate biological assays and microscopy conditions were chosen once the fluorescent properties of the ligand precursors and complexes are understood in solution. Therefore, fluorescence excitation scans and corresponding fluorescence emission scans between 200–800 nm were obtained for stock solutions of 100 in μM DMSO. Furthermore, ranges of absorption and emission were assessed using 2D contours, which haven used as tools in subsequent choice of cytotoxicity assays and fluorescence imaging settings. The fluorescence of the bis(substituted) free ligands **3** and **4** was very weak, with the overall range of absorption for these compounds approximately between 260 nm and 550 nm and an emission range of 450 nm and 680 nm (see ESI† for further information) (Table 2).

**Table 2:** Fluorescence spectroscopy data of bis(thiosemicarbazonato) ligand precursors and complexes in DMSO at a concentration of 100 μM

Compound	*λ*_ex-max_/nm	Excitation range/nm	*λ*_em-max_/nm	Emission range/nm
**2**	490	240–615	554	454–735
**3**	490	380–530	550.5	496–663
**4**	480	260–560	547	499.5–695
Ga(**3**)	500	250–270	556	500–685
Ga(**4**)	520	250–550	554	490–690

**Fig. 6:**
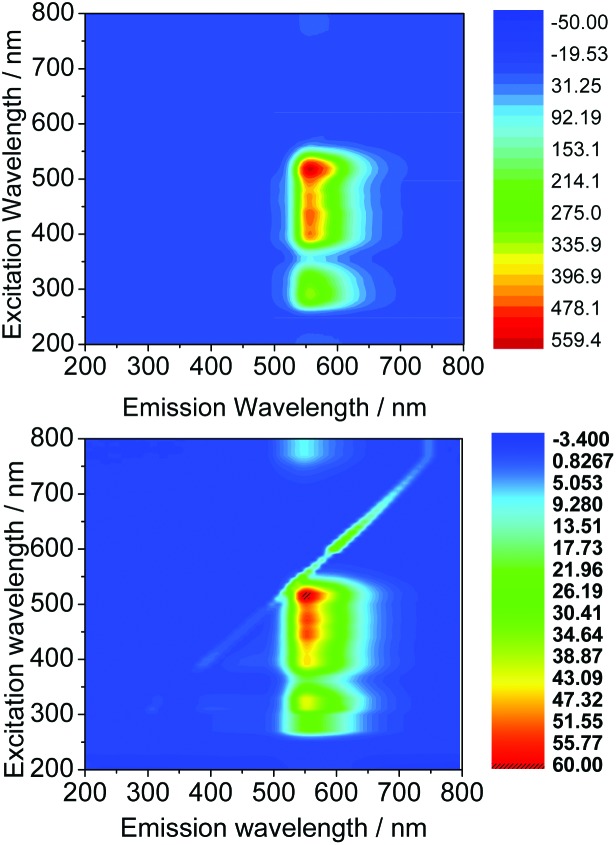
Excitation/emission 2D map of Ga(**3**) (above) and Ga(**4**) (below) in DMSO at a 100 μM concentration.

The wavelengths resulting in maximum excitation (*λ*_ex-max_) were found between 470 to 490 nm, indicating that compounds would be appropriate for excitation *via* the 488 nm laser of a confocal microscope. A kinetic stability estimation was conducted *in vitro* for the ligand precursors in order to allow comparisons with the previously reported corresponding metal complexes. Complexes Ga(**3**) and Ga(**4**) showed to be sufficiently stable in the timescale of the imaging experiment with respect to decomposition in a mixture of DMSO and several aqueous buffers (see the ESI† for further details) which are of relevance to standard cellular imaging assays (Fig. 6).

Additionally, it is generally accepted that prior to performing hypoxia selectivity tests in cellular environments, the redox chemistry of complexes based upon the ATSM core needs to be assessed in solution, since it has been ascertained that their biologically accessible *E*_1/2_ is a critical parameter in explaining the differences between identical ligands with differing metal centres (*i.e.* copper *versus* zinc, nickel). It was noticed that ligand 3-bound Copper(ii) has an *E*_1/2_ of –0.54 V, almost identical with that of the Cu(ii) ATSM hypoxia tracer, however, that did not show hypoxia selectivity on a 1 h experiment timescale including [^64^Cu](**3**).

In this case, the redox chemistry of the gallium complexes of the ligands **3** and **4** was also tested by cyclic voltammetry using a standard three-electrode electrochemical setup in dry and degassed DMF, adding tetrabutylammonium hexafluorophosphate as supporting electrolyte in a 0.1 M concentration and ferrocene as an internal standard. For complexes present in a 1 mM concentration, no oxidation or reduction waves were observed for Ga(**3**) or Ga(**4**) in the cyclic voltammograms, suggesting these complexes would likely be stable to redox processes inside normal redox potentials found in cells. This is as expected given their close structural similarity to the redox-chemistry inert Zn(ii) species anchored onto N/S. Unlike in the case of CuATSM, where the redox process is associated to the release of copper and hence to the hypoxia selectivity, gallium does not undergo a redox process to be released *in vitro*. Once in the cell the metal would be trapped by one of the potent iron chelators such as DFO found in cells and ultimately, gallium would be tracking changes in iron metabolism that are established to change under hypoxia.^31^

**Fig. 7:**
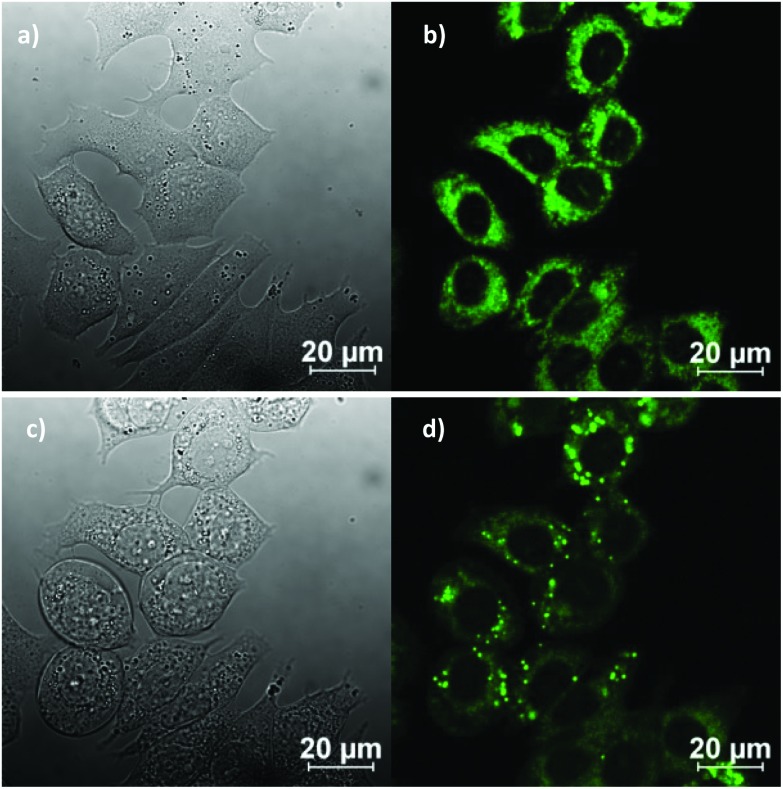
Representative confocal fluorescence imaging of the complex Ga(4) at 50 μM in 0.5% DMSO, for 20 min, at 37 °C continuous illumination experiment at 488 nm in MCF-7 breast cancer cells: before irradiation (a and b) and after irradiation (c and d) for *ca.* 10 min, where DIC image (a, c), micrograph of cells after excitation at 488 nm (b, d, compound, green channel).

**Fig. 8:**
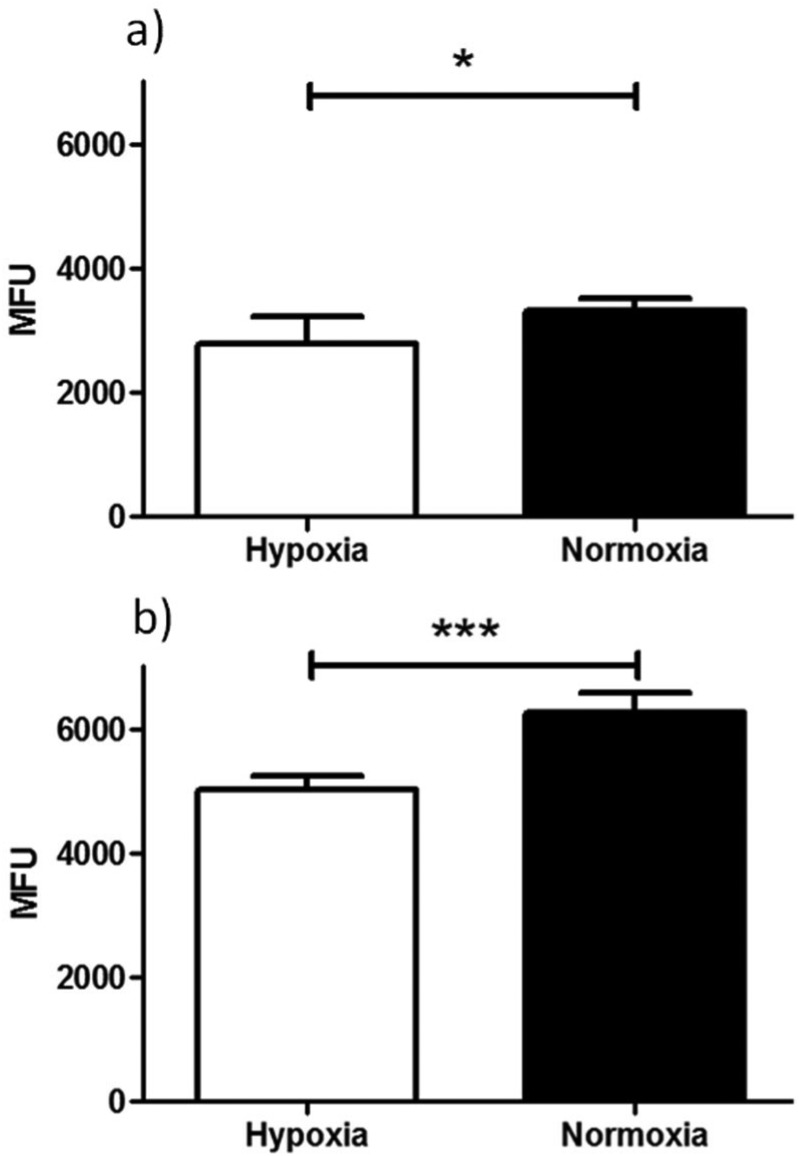
Flow cytometry results of ‘cold’ compound Ga(4) in (a) EMT6 and (b) PC-3 cells under hypoxic (white bars) and normoxic (black bars) conditions. Hypoxia in cells was induced for 20 min prior to addition of compound in 1% O_2_ and for a further 20 min in the same conditions post addition of the compound. Data shown are median fluorescence intensities (MFU) ± SD (*n* = 3, samples run in triplicates). MFU values are in arbitrary units (a.u.). (Exc, *λ* = 490 nm; Em, *λ* = 525 nm, *x*-axis). Staining is significantly lower in cells incubated under hypoxic conditions than under normoxic conditions for both EMT6 (*P < 0.05, *p* = 0.0204) and PC-3 (***P < 0.001, *p* = 3.2512 × 10^–6^) cell lines. For both cell lines a significant difference in fluorescence was observed under hypoxia *vs.*. normoxia (*P < 0.05 and ***P < 0.001).

*In vitro* imaging experiments were carried out by confocal multiphoton laser scanning microscopy, coupled with fluorescence lifetime imaging (excitation at 810 nm and 910 nm), which ascertained that (although weak) the fluorescence emission of the ligand precursors and corresponding complexes as sufficient to be observed *in vitro*. Cells were cultured using the analogous protocols to earlier investigations on fluorescent thiosemicarbazones (ESI). The compounds were loaded into EMT6 (breast cancer), HeLa (cervical carcinoma), PC-3 (prostate carcinoma), MCF-7 (breast cancer) as well as FEK-4 (non-cancerous fibroblast) cell lines and imaged using standard confocal fluorescence microscopy using single-photon excitation at 488 nm. The imaging studies were performed using concentrations of 50 μM in a DMSO : RPMI cell medium 1 : 99 solvent mix, whereby the final DMSO concentration on the imaging plate was lower than 1%. Fig. 7 shows representative images in the series and full details of the imaging assays are given in ESI.† (Fig. 8).

Experiments showed that the bis(substituted) free-ligand **4** possessed weak uptake in HeLa cells, which was barely detectable when incubated in FEK-4 cells, under the same conditions in contrast to the strong uptake seen with Ga(**4**) (see ESI† for further information). The weak fluorescence of the ligand precursor within cells constitutes a significant advantage in being able to assess the stability of their respective metal complexes *in vitro* in that significant emission using concentrations below 100 μM would be indicative of presence of the complex rather than the ligand precursor.

**Fig. 9:**
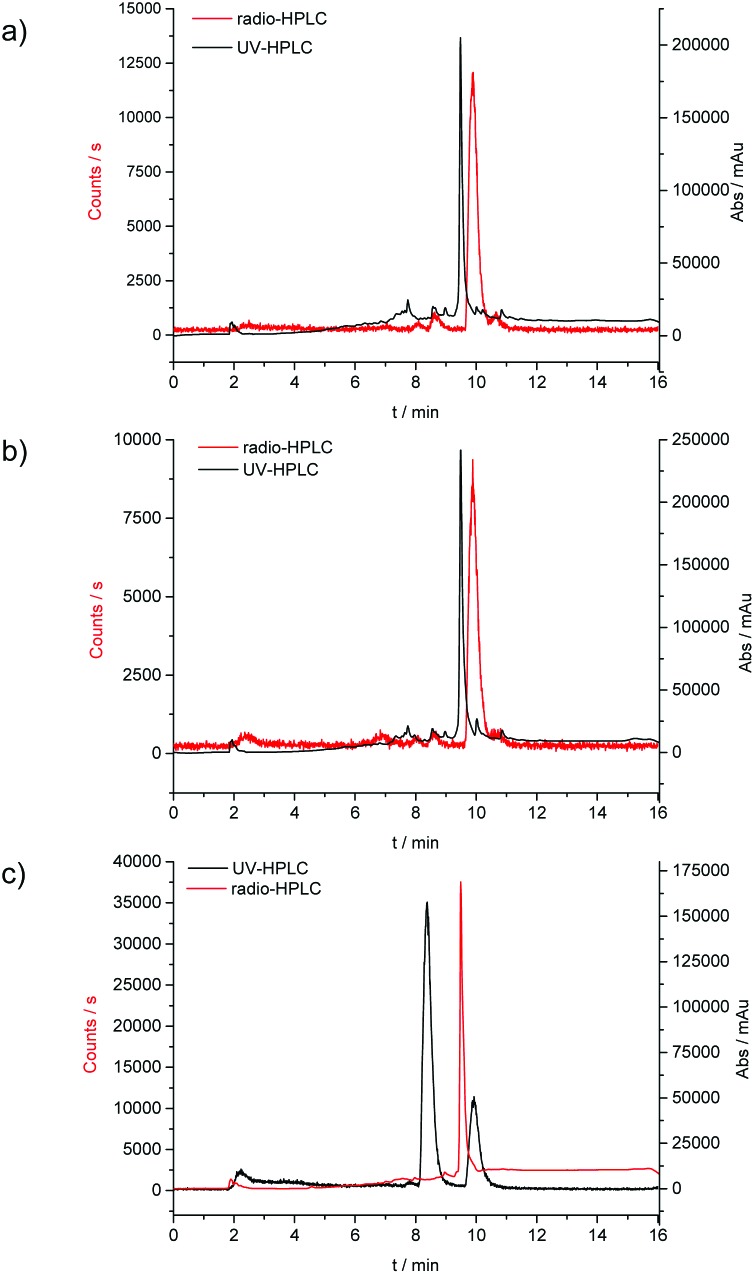
Overlay of the UV-HPLC trace (black) and the radio-HPLC trace (red) for the radiolabelling experiments; (a) conventional synthesis, 30 min reaction time, (b) microwave synthesis, 10 min reaction time and (c) microwave synthesis, 10 min reaction time scale up for micro PET experiments.

An initial investigation to assess the hypoxic selectivity of the gallium complex Ga(**4**) was carried out in EMT6 and PC-3 cell lines using flow cytometry (Fig. 9). Interestingly, whilst the changes seen were small, a significant decrease in fluorescence was observed in both EMT6 and PC-3 cell lines under hypoxia than observed under normoxia, (17% lower for EMT6 (*P < 0.05, *p* = 0.0204) and 22% lower in PC-3 cells (***P < 0.001, *p* = 3.2512 × 10^–6^)). The most likely explanation for the reduction in fluorescence is that the gallium metal centre is decomplexed from the molecule, leaving the free ligand and what is assumed to be gallium hydroxide (since the experiment was carried out in 96% aqueous buffer), neither of which have any noticeable fluorescent signal from experiments described earlier. These results suggest that there is a definite effect as a result of hypoxic conditions, thus further *in vitro* characterisation was carried out using radiochemical retention experiments.

### Ga-68 radiochemical synthesis

**Scheme 2:**
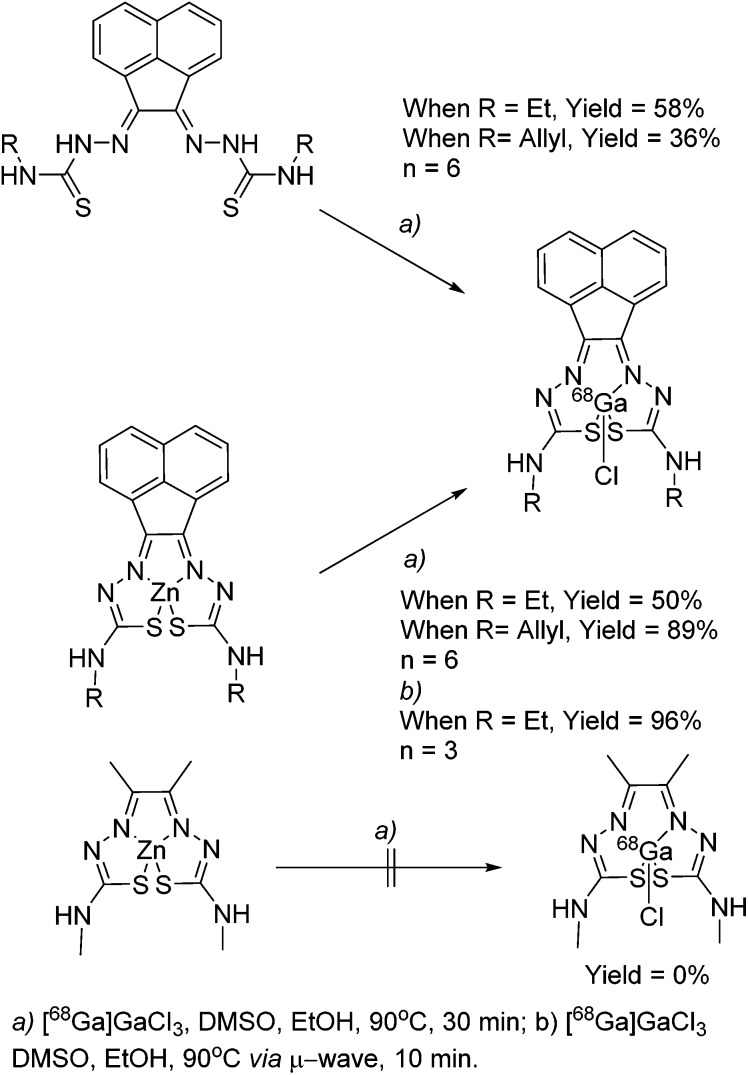
^68^Ga radiolabelling of compounds [^68^Ga]**3** and [^68^Ga]**4**.

General procedures for the chemical synthesis of the Zn(ii) precursors required for ^68^Ga labelling under prolonged heating conitions has been described previously for R = allyl and an optimised procedure was carried out here as a control. ^68^Ga radiolabelling was carried out on both free proligand precursors the corresponding zinc-chelated complexes (Scheme 2). ^68^Ga was eluted from a generator containing ^68^Ge using 0.1 M HCl, before being reformulated into a solution containing acetone or dry THF and 0.02 M HCl. When this was used directly for radiolabelling, only free ^68^Ga was identified, and it appeared as though all of the ligand starting material had been degraded in the UV spectra. To counteract this, the complete drying the ^68^Ga solution to remove all acetone and HCl using a stream of dry N_2_ or Ar (at 110 °C on a heating block) was carried out as these traces of solvent/acid appeared to interfere with thiosemicarbazone formation. The ^68^Ga residue, trapped onto the walls of the borosilicate glass test-tube used, was re-dissolved in neat ethanol or dry THF. Depending on the reaction scale, brief sonnication helped ensure that most of the ^68^Ga embedded within the glass walls is solubilised, and this procedure was carried out under a static atmosphere of Ar (pH *ca.* 7–8 was adjusted using NaOAc). The complex was subsequently added in DMSO and gave a successful incorporation of 89% of compound [^68^Ga]**4** (*n* = 6; decay-corrected for the allyl variant of the complex) from its zinc-metallated analogue.

Further labelling experiments gave the ethyl variant in a 50% d.c. yield of compound **[** ^68^Ga]**3** when the conventional heating strategy was used (in a glass vial, on a heating block held at 90 °C).

In a similar fashion, both ethyl and allyl variants of the modified bis(thiosemicarbazone) backbone without zinc pre-complexed gave moderate radiochemical incorporations of ^68^Ga, with d.c. yields of 36% and 58% respectively. When the reaction time was increased from 30 to 45 min, incorporation of ^68^Ga increased from 89 to 95%, but the increased time (and therefore decay) negated any potential advantage to this. Total synthesis time was found to be around 60 min, making it appropriate for its use in biological investigations, as it is within one half-life of ^68^Ga.

Interestingly, we found that when radiolabelling with ^68^Ga was carried out by transmetallation from a zinc-chelated **3** *via* microwave under Argon and using EtOH or THF as the solvent of choice, the reaction went to completion within 10 min, giving a radiochemical incorporation over 95%. This suggested that gallium-chelation reactions, carried out under kinetic control as it is the case for radiochemical incorporations, could be sped up significantly when using a microwave reactor instead of traditional heating. This process was scaled up to the use of 111 MBq gallium-68 precursor, and the product isolated by a semi-prep HPLC to allow for *in vivo* imaging by micro PET. As a result of the significant reduction in radiochemical process, the entire radiolabelling procedure including semi-prep purification and concentration of the [^68^Ga]**4** radio-tracer under reduced pressure (by rota evaporator) and pH adjustment (using NaOAc buffer, to 8.4) rendered this tracer ready for *in vivo* PET imaging experiments in less than 2 h (ESI).

**Fig. 10:**
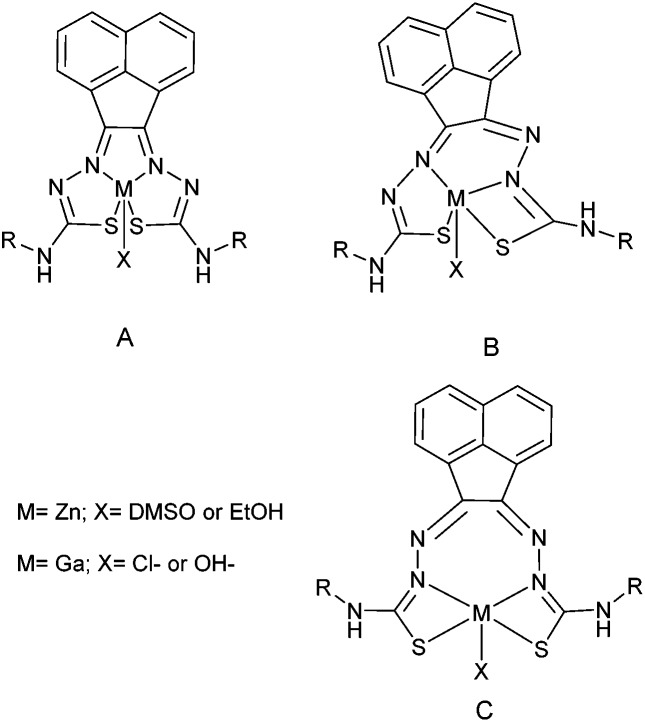
Proposed coordination isomers of the bis(thiosemicarbazone) ligand to the metal centre. A and B forms were observed by X-ray crystallography and NMR. C is considered but not isolated or characterised. All species were observed in HPLC and radio HPLC.

To probe the kinetic stability of the ^68^Ga-labelled compounds, we carried out several standard assays of relevance to tracer preparation for *in vivo* imaging. Citric acid (1 hour, 75% parent compound with respect to decomposition to free hydrated gallium ions), EDTA (1 hour, 40% parent compound with respect to decomposition to free hydrated gallium ions), and saline/PBS (upto 4 hours, >99% parent compound remaining) were all used to look at the stability of the labelled complex [^68^Ga]**4**. Over 24 hours in an aqueous environment, isomerisation equilibria occurs as suggested by HPLC traces (R_*t*_ = 7 min, 8.5 min and 10 min), in a ratio of approximately 1 : 1 : 1. When any of these individual fractions was collected by semi-prep HPLC and re-injected on the HPLC for analysis, using the same method after 1 hour, the formation of the original, parent compound (R_*t*_ = 10 min) was observed. Subsequent ^1^H NMR experiments involving non-labelled gallium complexes complemented this study and confirmed the existence of several isomers in aqueous solution, most likely influenced by protonation of the complex under HPLC conditions (H_2_O/CH_3_CN, TFA) (ESI) (Fig. 10).

For a direct comparison to the tracers in clinical trial for hypoxia, the ^68^Ga labelling of Zn-ATSM (a typical precursor for the formation of [^64^Cu]Cu-ATSM) was attempted using the same reaction conditions. Despite numerous attempts and subsequent variations, no product could be identified by HPLC, with only free hydrated ^68^Ga ions present at 2–3 min by radio HPLC. This strengthens the need for a modified backbone to the bis(thiosemicarbazonato) structure for the incorporation of gallium, to allow for a higher kinetic stability with respect to association/dissociation post-radiolabelling.

### Radiochemical *in vitro* assays with gallium-68 complexes

**Fig. 11:**
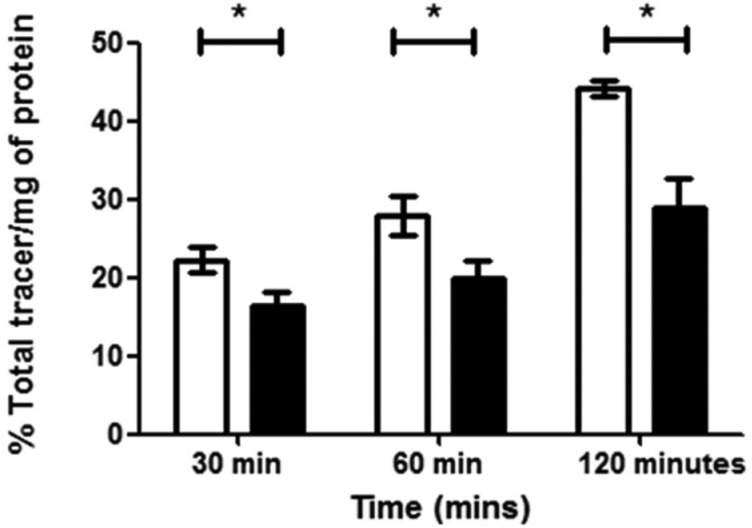
[^68^Ga]**4** retention in EMT6 cells under hypoxic (white bars) or normoxic conditions (black bars) was measured at 30, 60 and 120 min post addition of tracer and is expressed as % of total tracer/mg of protein. Hypoxia in cells was induced for 20 min prior to addition of the tracer in 1% O_2_ and cells were maintained under the same conditions for the remainder of the time course. Higher retention is observed under hypoxic conditions. Data represents mean values ± SD (*n* = 3), (*P < 0.05, p values at 30, 60 and 120 min are 0.04235, 0.0367 and 0.02360 respectively).

The complex [^68^Ga]**4**, radiolabelled as described and then purified *via* solid phase extraction, was incubated with EMT6 cells under normoxia and hypoxia (induced as described in ESI,† and compared with standard hypoxia assays). During the time of the experiment, aliquots were isolated, centrifuged and the radioactivity associated with the resulting pellet estimated with a well counter. Subsequently a time course of the Ga-68 labelled compound 4 retention was analysed. Control experiments were also performed using ^68^GaCl_3_ as-made from the generator to eliminate the possibility that the active species may be the dissociated ligand-free aqueous ^68^Gallium(iii). At each time point, there was a significant increase in ^68^Ga retention under hypoxia compared to normoxia, with the difference being as high as 53% after 2 hours (*p* = 0.02360) (Fig. 11). The EMT6 model has been used extensively in previous studies with [^64^Cu]Cu-ATSM, giving a 500% increase in retention between hypoxic and normoxic cells (in part, due to the extremely low retention observed in normoxic tissue), which is most likely indicative that **[** ^68^Ga**]** **4** log *P* is significantly greater than that of [^64^Cu]Cu-ATSM, leading to a higher background signal and lower differential, which increases from 30 min through to 120 min, albeit at a lower level than the increase in hypoxic retention.

Strikingly, the experiments clearly show a response to hypoxia which is measureable and statistically significant. This was unexpected due to the chemical and redox differences in the copper(ii) and gallium(iii) variants of this family of complexes. Experiments were also carried out using [^68^Ga]GaCl_3_ under similar conditions, which showed minimal retention, with no differences between hypoxia and normoxia, proving that the retention seen for [^68^Ga]**4** is at least partially ligand-controlled rather than simply any gallium biological pathway. As the complexes described within this report are structurally similar to that of Cu-ATSM and its related family of compounds, conclusions on the possible mechanism of selectivity could be drawn, despite the change in metal co-ordination centre from copper to gallium. In the case of Cu-ATSM, recent work has shown that the ligand backbone system is required to get hypoxia selectivity *in vitro* but simple [^64^Cu]Cu(OAc)_2_ gives identical hypoxia selectivity *in vivo*.^12,14^ As indicated in the data we have acquired using flow cytometry, it appears likely that our Ga-BTSC complexes are able to pass inside the cell, leading to dissociation upon hypoxia. How these observations will translate *in vivo* is currently difficult to predict, with knowledge of any gallium pathways in biology much more limited than that of copper pathways, but work is on-going to try and elucidate this. Preliminary *in vivo* testing in mice using [^68^Ga]**4** showed rapid excretion through the bladder (5 min–1 h) and only a slight accumulation in liver after 4 hours imaging observation (see ESI† for further details), similar to the distribution seen when using uncomplexed [^68^Ga]GaCl_3_.

## Conclusions

Although hypoxia has been a key area of PET tracer research since the introduction of [^18^F]FMISO in 1984, the desired ‘knockout’ hypoxia tracer remains to be discovered. The problems indicated with current gold standards, both emergent from this work and within the current literature show that the clinic is still waiting for a robust, reliable and effective hypoxia radiotracer that can be routinely used and give results within a 60 minute PET scan.

The data presented here suggests that a symbiosis of the short-lived isotope gallium-68 and the ligand family of aromatic bis(thiosemicarbazones) could bring this quest a step closer towards completion with the advancements in radiosynthesis due to microwave technologies. Gallium generators are now wide-spread throughout hospitals in GMP radiopharmacies, and the possibility of delivering the tracer to the clinician within one half-life and getting decisive *in vivo* retention within a further 90 min suggests it is worth pursuing.

To summarise, we have successfully synthesised several [^68^Ga]Ga-BTSC analogues, having already looked at their structure and co-ordination using X-ray crystallography. Furthermore, non-labelled chemistry work using ^1^H NMR, fluorescence microscopy and cyclic voltammetry add to the picture of kinetic stability in biological media in the timescale of the imaging experiment and shed some light into the biological fate of the complexes, while both flow cytometry and ^68^Ga radiochemical retention assays strongly indicate that there is hypoxic retention response of these complexes, when compared to simple [^68^Ga]GaCl_3_ and [^64^Cu]Cu-ATSM retention. Work is now on-going in two directions in our laboratories: (1) pursuing the lead compound **[** ^68^Ga**]** **4** *in vivo* to look at its retention in differing tumour models and conditions and (2) further investigations into the mechanism of this complex in light of recent publications discussing the mechanism and selectivity of [^64^Cu]Cu-ATSM against [^64^Cu]Cu(OAc)_2_.

## Experimental section

All reagents were purchased from Sigma-Aldrich, Alfa-Aesar, Merck Chemicals or Acros Organics and were used as received unless otherwise stated. Microwave reactions were performed in a Biotage Initiator 2.5 system in 5 mL glass capped vials. The reaction mixture was prestirred for 30 s and then heated for the selected time. If the irradiation power is not set, it reaches its maximum (300 W from magnetron at 2.45 GHz) at the start of the reaction until the target temperature is reached, decreasing to lower values afterwards. ^1^H NMR spectra were recorded on a Bruker Avance spectrometer (300 MHz, 400 MHz) or a Bruker Avance II+ (500 MHz) spectrometer at 298 K. Chemical shifts *δ* in ppm were referenced to the solvent residual peak as an internal standard. Peak multiplicities are given as follows: s, singlet; d, doublet; t, triplet; q, quartet; qt, quintet and m, multiplet. ^13^C NMR was recorded on a Bruker Avance spectrometer (300 MHz, 400 MHz) or a Bruker Avance II+ (500 MHz) and spectra was referenced to the solvent residual peak.

Mass spectrometry was performed in a Bruker Micromass LCT TOF spectrometer under conditions of electrospray ionization. Accurate masses are reported to the fourth decimal place using tetraoctylammonium bromide (466.5352 Da) as an internal standard.

HPLC was carried out using a Phenomenex Ultracarb C-18 column (4.6 × 150 mm) with UV/visible detection measured at up to four wavelengths; *λ*_obs_ = 254 nm. The gradient elution was 1.0 mL per minute, with 0.1% TFA MilliQ water as solvent A and 0.1% TFA MeOH as solvent B. Start 95% A reverse gradient until 5% A at 12 min, hold until 15 min.

UV-visible spectra were obtained using a Lamda 650 Perkin-Elmer Spectrometer in DMSO and processed using UV Winlab 3 software. The orientation of the 1.00 cm quartz cuvette was the same for each experiment for consistency.

Fluorescence spectra were measured in a LS55 Perkin-Elmer luminescence spectrophotometer using a 1.00 cm quartz cuvette. A scan from 200–800 nm with increments of 10 nm was initially carried out to discover excitation wavelength of maximum emission (*λ*_ex-max_).

General procedure for microwave-assisted synthesis, A: acenaphthenequinone (0.200 g, 1.04 mmol) and the corresponding thiosemicarbazide (1.04 mmol) were suspended in 5 mL of ethanol in a microwave vial. Then, 3 drops of conc. HCl added were added and the vial was sealed. The reaction mixture was prestirred for 30 s and then heated to 90 °C for 9 min. The yellow solid was filtered whilst hot, resuspended in hot methanol, stirred for 15 min, washed with methanol and diethyl ether and dried under vacuum.

General procedure for microwave-assisted synthesis, B: acenaphthenequinone (0.200 g, 1.04 mmol) and the corresponding thiosemicarbazide (3.12 mmol) were suspended in 5 mL of ethanol in a microwave vial. Then, the procedure continues as in A.

Compound **1** was prepared following the method of Pascu *et al.* 2010 (ref. 20) and by microwave synthesis, general procedure A (216.5 mg, 73%). Compound **2** was prepared following the method of Pascu *et al.* 2011 (ref. 21) and by microwave synthesis, general procedure A (237.2 mg, 77%).

### Bis(4-ethyl-3-thiosemicarbazone) acenaphthenequinone (**3**)

Acenenaphthenequinone (0.25 g, 0.137 mmol) and 4-ethyl-3-thiosemicarbazide (0.48 g, 0.411 mmol) were suspended in 40 mL ethanol and refluxed for 4 h. 10 drops of conc. HCl were added upon reflux. The solid was isolated by filtration whilst hot, resuspended in hot methanol (10 mL) and stirred for 15 min before filtering and washing with further methanol. The resultant yellow solid (446.7 mg, 85%), compound 3, was dried under vacuum. Microwave synthesis: method B (359.0 mg, 90%).

^**1**^ **H NMR** (300 MHz, d_6_-DMSO, 25 °C): *δ* 12.55 (s, 1H, N-NH′), 11.21 (s, 1H, N-NH), 9.19 (t, 1H, NH′Et, *J* = 6.0 Hz), 8.80 (t, 1H, NHEt, *J* = 5.5 Hz), 8.19 (d, 1H, H-1, *J* = 7.2 Hz), 8.13 (d, 1H, H-3′, *J* = 8.3 Hz), 8.09 (d, 1H, H-1′, *J* = 7.0 Hz), 8.03 (d, 1H, H-3, *J* = 8.3 Hz), 7.79 (overlapping t, 2H, H-2 and H-2′), 3.66 (m, 4H, CH_2_CH_3_), 1.24 (m, 6H, CH_2_CH_3_). ^**13**^ **C NMR** (75.5 MHz, d_6_-DMSO, 25 °C): *δ* 178.77, 176.92, 139.25, 139.16, 136.66, 133.06, 130.04, 128.69, 128.58, 128.12, 128.35, 126.17, 124.64, 119.60, 31.05. **Mass spectrum** ESI-MS Calc. for C_18_H_19_N_6_S_2_ [M – H]^–^ 383.1113, found 383.1155. **Elemental Analysis**: Found C; 56.1%, H, 5.23%, N; 21.8%. Calc.: C; 56.22%, H; 5.24%, N; 21.86%.

### Bis(4-allyl-3-thiosemicarbazone) acenaphthenequinone (**4**)

Acenaphthenequinone (0.25 g, 0.137 mmol) and 4-allyl-3-thiosemicarbazide (0.54 g, 0.411 mmol) were suspended in 40 mL ethanol and refluxed for 4 h. 10 drops of conc. HCl were added upon reflux. The solid was isolated by filtration whilst hot, resuspended in hot methanol (10 mL) and stirred for 15 min before filtering and washing with further methanol. The resultant yellow solid **4** (415 mg, 74%) was dried under vacuum. Microwave synthesis: method B (402.4 mg, 94%).

^**1**^ **H NMR**: (300 MHz, d_6_-DMSO, 25 °C): *δ* 12.63 (s,1H, N-NH′), 11.27 (s,1H, N-NH), 9.32 (t, 1H, NHAllyl, *J* = 6.0 Hz), 9.02 (t, 1H, NH′Allyl, *J* = 5.7 Hz), 8.21 (d, 1H, H-1 or H-1′, *J* = 7.2 Hz), 8.11 (overlapping d, 2H, H-3 and H-3′), 8.01 (d, 1H, H-1 or H-1′, *J* = 7.1 Hz), 7.80 (overlapping t, 1H, H-2 or H-2′), 7.78 (overlapping t, 1H, H-2 or H-2′), 5.95 (m, 2H, CH2CHCH2) 5.35 + 529 (ddt, 2H, H *trans*, *J* = 17.4 Hz), 5.25 + 5.18 (ddt, 2H, H *trans*, *J* = 17.4 Hz), 5.17 + 5.14 (m, 2H, Hcis, *J* = 10.6 Hz), 4.31 (m, 4H, CH_2_CHCH_2_). ^**13**^ **C NMR**: (75.5 MHz, d_6_-DMSO, 25 °C): *δ* 182.52, 178.14, 138.23, 136.45, 134.86, 134.40, 133.21, 130.32, 129.28, 128.88, 128.55, 128.18, 127.09, 124.93, 116.83, 116.28, 47.07, 46.52. **Mass spectrum** ESI-MS Calc. for C20H19N6S2 [M – H]^–^ 407.1113; found 407.1106. **Elemental Analysis**: Found C; 58.9%, H; 4.94%, N; 20.5%. C20H20N6S2. Calc.: C; 58.80%, H, 4.93%, N; 20.57%.

### Zinc(ii) bis(4-ethyl-3-thiosemicarbazone) acenaphthenequinone Zn(**3**)

Gallium(iii) chloride bis(4-allyl-3-thiosemicarbazone) acenaphthenequinone was prepared following the method of Pascu *et al.* 2008.^34^

### Zinc(ii) bis(4-allyl-3-thiosemicarbazone) acenaphthenequinone Zn(**4**)

Gallium(iii) chloride bis(4-allyl-3-thiosemicarbazone) acenaphthenequinone was prepared following the method of Pascu *et al.* 2007.^32^

### Gallium(iii) chloride bis(4-ethyl-3-thiosemicarbazone) acenaphthenequinone Ga(**3**)

A suspension of zinc bis(4-ethyl-3-thiosemicarbazone) acenaphthenequinone (0.216 g, 0.482 mmol) in MeOH (50 mL) was added to GaCl_3_ (0.420 g, 1.478 mmol). The resulting suspension was heated at reflux for 6 h. The red solid that precipitated on cooling to room temperature was then isolated by filtration, washed with Et_2_O and dried under vacuum (0.146 g, 0.299 mmols, 62%). The (*E*,*E*) and (*E*,*Z*) isomers were found to be present in the integral ratio of approximately 1 : 1 in d_6_-DMSO by ^1^H NMR analysis. (*E*,*Z*) isomer (51%). ^**1**^ **H NMR** (300 MHz, d_6_-DMSO, 25 °C): *δ* 9.58 (t, 1H, NH), 8.87 (t, 1H, NH′), 8.50 (d, 1H, H-1, *J* = 7.0 Hz), 8.11 (d, 1H, H-3′, *J* = 8.3 Hz), 8.03 (d, 1H, H-3, *J* = 8.1 Hz), 7.93 (d, 1H, H-1′, *J* = 6.9 Hz), 7.83 (apparent t, 1H, H-2), 7.76 (apparent t, 1H, H-2′), 3.61 + 3.56 (m, 4H, CH2), 1.26 + 1.25 (two t, 6H, CH3, *J* = 7.2 Hz); (*E*,*E*) isomer (49%): *δ* 8.79 (broad t, 2H, NH), 8.16 (d, 2H, H-1, *J* = 7.0 Hz), 8.09 (d, 2H, H-3, *J* = 8.3 Hz), 7.75 (apparent t, 2H, H-2), 3.59 (m, 4H, CH2), 1.25 (t, 6H, CH3, *J* = 7.2 Hz). ^**13**^ **C NMR** (75.5 MHz, d_6_-DMSO, 25 °C): *δ* 177.56, 173.20, 169.93, 138.72, 138.50, 135.84, 132.28, 130.25, 129.65, 129.31, 128.92, 128.47, 127.00, 126.83, 126.06, 124.23, 118.39, 114.13, 41.87, 38.34, 37.57, 14.60, 14.07, 13.67. **Mass spectrum** ESI-MS Calc. for C_18_H_17_ClGaN_6_S_2_ [M – H]^–^ 484.9900; found 484.9907.

### Gallium(iii) chloride bis(4-allyl-3-thiosemicarbazone) acenaphthenequinone Ga(**4**)

Gallium(iii) chloride bis(4-allyl-3-thiosemicarbazone) acenaphthenequinone was prepared following the method of Pascu *et al.* 2011.^21^

### Radiochemistry labelling procedures

10 mL of 0.1 M HCl was used to elute *ca.* 222 MBq of ^68^Ga^3+^ from the generator and was subsequently trapped on a 30 mg mL^–1^ Strata X-C cartridge. This was eluted with 700 μL of 0.02M HCl/98% acetone and dried for 15 min under a stream of nitrogen or argon at 110 °C Alternatively, 1 N aq. HCl may also be used. Next, 25 μL of 2 mg mL^–1^ zinc complex precursor in DMSO and 2 mL of HPLC-grade THF were added. The solution was heated for 30 min at 90 °C, or *via* a microwave for 10 min set to 90 °C. The HPLC methods above were used to analyse the reaction mixtures.

### Cell culturing and cell plate preparation

Cells were cultured at 37 °C, at 5% CO_2_ in a humidified atmosphere and passaged once confluence had been reached. Culture occurred in Eagle's Minimum Essential Medium (EMEM) for HeLa (human cervical cancer cells) and FEK-4 (epithelial fibroblast cells), Dulbecco's Modified Eagle's Medium (DMEM) for MCF-7 (human breast cancer), RPMI 1640 for PC3 (human prostate cancer cells) and Waymouth's medium for EMT6 (murine breast carcinoma cells). The media contained foetal calf serum (FCS) (10% for HeLa, PC-3 and MCF-7 and 15% for FEK-4 and EMT6 cells), 0.5% penicillin/streptomycin (10 000 IU mL^–1^/10 000 mg mL^–1^) and 200 mM l-Glutamine (5 mL). All steps were performed in absence of phenol red. Cells were trypsinised and counted using a haemocytometer and then seeded as appropriate for the biological assays.

### Fluorescence microscopy

Confocal fluorescence microscopy experiments were carried out on a Zeiss LSM510META microscope under normoxic conditions. For all fluorescence microscopy experiments, cells were cultured as above and plated in glass-bottomed dishes as 1.5 × 105 cells per dish (*ca*. 60% coverage) and incubated for 12 h for HeLa, EMT-6 and FEK-4 cells. All steps were carried out in the absence of phenol red. Prior to compound addition, cells were washed 3 times with PBS, before adding serum free medium (1 mL). Subsequently, a small volume of medium was removed (10 μL) and compound in DMSO was added to obtain a final volume of 1 mL and the desired concentration. The final concentration of compounds on the cell plate was 50 μM in medium, containing 0.5% DMSO or 1% DMSO, depending on the compound solubility. After 20 min with the compound cells were washed 3 times with PBS and fresh serum free medium was added (1 mL) and images were recorded immediately.

### Flow cytometry studies

Cells were seeded as 3 × 105 cells per well in a 6 well plate and incubated overnight. The cells were subsequently washed twice with PBS before incubation with the compound at 50 μM, 4% DMSO final concentration in serum free media. This was incubated for 20 min at 20.7% O_2_ and 5% CO_2_ at 37 °C for normoxic samples. Hypoxic conditions were obtained by pre-incubating the cells for 20 min at 1% O_2_ and 5% CO_2_ at 37 °C, followed by incubation for a further 20 min under the same conditions with the compound. Following this, cells were washed three times with PBS, trypsinised and centrifuged at 600*g* for three min. The pellet was washed with PBS, resuspended in 1 mL of serum free medium, kept on ice and analysed with a LSRII cytometer (BD Biosciences, Rockville, MD USA), with 10 000 cells counted per event. Each experiment was carried out at least three times, with data analysis performed using FlowJo software (TreeStar, USA).

### Radioactive cell retention investigation

Cells were seeded as 3 × 105 cells per well in a 6 well plate and incubated for *ca.* 12 h. The cell medium was aspirated and replaced with serum free medium containing the ^68^Ga radiolabelled complex (following the radiochemistry procedure above). This was incubated at 20.7% O_2_ and 5% CO_2_ at 37 °C for normoxia. Hypoxic conditions were obtained by pre-incubating the cells for 20 min at 1% O_2_ and 5% CO_2_ at 37 °C, followed by incubation under the same conditions with the compound for the time course of the study. Cell plates were subsequently placed on ice, washed 3 times with ice-cold PBS and lysed using 0.2 mL RIPA buffer for 10 min (Thermo Fisher Scientific Inc., Rockford, IL, USA). PBS (0.5 mL) was added to each well and cell lysates were transferred to counting tubes, with measurements of decay-corrected radioactivity performed using a gamma counter (Cobra II Auto-Gamma counter, Packard Biosciences Co, Pangbourne, UK). Aliquots were snap-frozen and subsequently protein determination was carried out using a bicinchoninic acid assay (BCA) assay (Thermo Fisher Scientific Inc., Rockford, IL, USA). Decay corrected counts were corrected to protein concentration, with data presented as percent of total radioactivity per mg of protein.

### Statistical analysis

Data were expressed as mean ± standard error of the mean (SEM), unless stated in the text. Student's *t* test (Prism v5.0 software for windows, GraphPad Software, San Diego, CA, USA) was used to assess the significance of comparison between two data sets. If *P* ≤ 0.05 the differences between groups were regarded as significant.

### X-ray crystallography

#### Crystal data for **3**

C_36_H_38_N_12_S_4_, *M* = 767.02, *a* = 8.535(2) Å, *b* = 19.120(5) Å, *c* = 22.172(6) Å, *α* = 90°, *β* = 90°, *γ* = 90°, *V* = 3618.2(16) Å^3^, *T* = 100(2) K, space group *P* 2_1_ 2_1_ 2_1_, *Z* = 4, *μ*(synchrotron) = 0.242 mm^–1^, 28 222 reflections measured, 6895 independent reflections (*R*_int_ = 0.2717). The final *R*1 values were 0.0993 (*I* > 2*σ*(*I*)). The final w*R*(*F* ^2^) values were 0.2565 (*I* > 2*σ*(*I*)). The final *R*1 values were 0.1037 (all data). The final w*R*(*F* ^2^) values were 0.2666 (all data). The goodness of fit on *F* ^2^ was 1.061. Flack parameter = 0.10(18). CCDC ; 1001632.

#### Crystal data for **4**

C_20_H_20_N_6_S_2_, *M* = 408.54, *a* = 7.859(7) Å, *b* = 11.040(9) Å, *c* = 12.049(9) Å, *α* = 90.546(6)°, *β* = 95.3880(10)°, *γ* = 110.304(13)°, *V* = 975.1(14) Å^3^, *T* = 293(2) K, space group *P*1, *Z* = 2, *μ*(synchrotron) = 0.227 mm^–1^, 6825 reflections measured, 3176 independent reflections (*R*_int_ = 0.0509). The final *R*1 values were 0.0573 (*I* > 2*σ*(*I*)). The final w*R*(*F* ^2^) values were 0.1398 (*I* > 2*σ*(*I*)). The final *R*1 values were 0.0802 (all data). The final w*R*(*F* ^2^) values were 0.1510 (all data). The goodness of fit on *F* ^2^ was 1.049. CCDC ; 1001633.

#### Crystal data for Ga(**3**)

C_18_H_18_ClGaN_6_S_2_·2(C_2_H_6_OS), *M* = 643.93, *a* = 11.55230(10) Å, *b* = 11.7595(3) Å, *c* = 12.0749(3) Å, *α* = 114.468(2)°, *β* = 105.4834(17)°, *γ* = 93.4737(18)°, *V* = 1411.20(6) Å^3^, *T* = 150(2) K, space group *P*1, *Z* = 2, *μ*(MoKα) = 1.397 mm^–1^, 50963 reflections measured, 12 291 independent reflections (*R*_int_ = 0.0414). The final *R*1 values were 0.0445 (*I* > 2*σ*(*I*)). The final w*R*(*F* ^2^) values were 0.1019 (*I* > 2*σ*(*I*)). The final *R*1 values were 0.0695 (all data). The final w*R*(*F* ^2^) values were 0.1163 (all data). The goodness of fit on *F* ^2^ was 1.023. CCDC ; 1001634.
