# Correction: Microwave gallium-68 radiochemistry for kinetically stable bis(thiosemicarbazone) complexes: structural investigations and cellular uptake under hypoxia

**DOI:** 10.1039/c6dt90021f

**Published:** 2016-02-08

**Authors:** Israt S. Alam, Rory L. Arrowsmith, Fernando Cortezon-Tamarit, Frazer Twyman, Gabriele Kociok-Köhn, Stanley W. Botchway, Jonathan R. Dilworth, Laurence Carroll, Eric O. Aboagye, Sofia I. Pascu

**Affiliations:** Department of Medicine, Imperial College Du Cane Road W12 0NN London UK l.carroll@imperial.ac.uk; Department of Chemistry, University of Bath Claverton Down BA2 7AY UK s.pascu@bath.ac.uk; Oxford Brookes University, Faculty of Health and Life Sciences, The Science and Technology Facilities Council, Rutherford Appleton Laboratory Harwell Oxford UK; Inorganic Chemistry Laboratory South Parks Road Oxford OX2 6TT UK

## Abstract

Correction for ‘Microwave gallium-68 radiochemistry for kinetically stable bis(thiosemicarbazone) complexes: structural investigations and cellular uptake under hypoxia’ by Israt S. Alam *et al.*, *Dalton Trans.*, 2016, **45**, 144–155.

The authors regret that in the published version of the above article the position of the footnote symbol (†) indicating three authors who contributed equally to the paper was incorrect. The footnote symbol should be positioned after the first three authors Israt S. Alam,† Rory L. Arrowsmith,† and Fernando Cortezon-Tamarit† as shown above.

The Royal Society of Chemistry apologises for these errors and any consequent inconvenience to authors and readers.

## Supplementary Material

