# Supplementary material for: Microwave gallium-68 radiochemistry for kinetically stable bis(thiosemicarbazone) complexes: structural investigations and cellular uptake under hypoxia
Source: Dalton Trans. 2015 Nov 19;45(1):144–55. doi: 10.1039/c5dt02537k (PMC4758186; doi:10.1039/c5dt02537k)
Supplement: Supplementary file 1 [file DT-045-C5DT02537K-s001.pdf]

Electronic Supplementary Material (ESI) for Dalton Transactions.
This journal is © The Royal Society of Chemistry 2015

Microwave Gallium-68 radiochemistry for kinetically stable *bis*(thiosemicarbazone) complexes: Structural investigations and cellular uptake under hypoxia

Israt S. Alam,[a]# Rory L. Arrowsmith,[b]# Fernando Cortezon-Tamarit,[b] # Frazer Twyman,[a] Gabrielle Kociok-Köhn,[b] Stanley W. Botchway,[c] Jonathan R. Dilworth,[d] Laurence Carroll,*[a] Eric O. Aboagye*[a] and Sofia I. Pascu*[b]

[a] Dr Israt S. Alam, Dr Laurence Carroll, Mr Frazer Twyman and Prof Eric O. Aboagye
Department of Medicine
Imperial College
Du Cane Road, W12 0NN

[b] Dr Rory L. Arrowsmith, Mr Fernando Cortezon-Tamarit, Dr Gabrielle Kociok-Köhn, and Dr Sofia Pascu*
Department of Chemistry
University of Bath,
Claverton Down, BA2 7AY
E-mail: s.pascu@bath.ac.uk

[c] Professor Stanley W. Botchway, PhD, MRSC
Oxford Brookes University, Faculty of Health and Life Sciences
The Science and Technology Facilities Council
Rutherford Appleton Laboratory, Harwell, Oxford

[d] Prof Jon Dilworth
Inorganic Chemistry Laboratory
South Parks Road
Oxford OX2 6TT

Table of Contents

1. General Information 3

2. Synthesis of the ligands 15

3. Fluorescence Spectroscopy 21

5. Radiochemistry 24

6. Laser scanning confocal microscopy 30

7. Two-photon excitation and fluorescence lifetime imaging microscopy 44

8. Kinetic stability testing in an aqueous environment 50

9. Stability assays to estimate kinetic stability against common biologically-relevant agents 55

10. *In vivo* imaging tests by microPET 62

11. Hypoxia selectivity testing 63

12. MTT assays 69

13. Cyclic voltammetry experiment of bis(thiosemicarbazonato) complexes and ligands 72

14. References 76

1. General Information

All reagents were purchased from Sigma-Aldrich, Alfa-Aesar, Merck Chemicals or Acros Organics and were used as received unless otherwise stated. ^1H NMR spectra were recorded on a Bruker Avance spectrometer (300 MHz, 400 MHz) or a Bruker Avance II+ (500 MHz) spectrometer at 298 K. Chemical shifts δ in ppm were referenced to the solvent residual peak as an internal standard. Peak multiplicities are given as follows: s, singlet; d, doublet; t, triplet; q, quartet; qt, quintet and m, multiplet. ^{13}C NMR was recorded on a Bruker Avance spectrometer (300 MHz, 400 MHz) or a Bruker Avance II+ (500 MHz) and spectra were referenced to the solvent residual peak. UV-visible spectra were obtained using a Perkin-Elmer Spectrometer, Lamda 650 in DMSO and processed using UV Winlab 3 software; the orientation of the 1.00 cm quartz cuvette was the same for each experiment for consistency. Fluorescence spectra were measured in a 1.00 cm quartz cuvette using a Perkin-Elmer LS55 luminescence spectrophotometer; a scan from 200-800nm with increments of 10 nm was initially carried out to discover excitation wavelength of maximum emission (λ_{ex-max}) and wavelength of maximum emission (λ_{em-max}). Elemental analysis were carried out by Mr Alan Carver, University of Bath.

1.1 Crystal structure determination by X-ray diffraction

Crystals were selected using the oil drop technique, in perfluoropolyether oil and mounted at 150(2) K with an Oxford Cryostream N_2 open-flow cooling device. Intensity data for **Ga(II)** was collected on an Nonius Kappa CCD single crystal diffractometer using graphite monochromated Mo-Kα radiation (λ = 0.71073 Å). The data was processed using the Nonius Software.[1] For **Ga(3)** data were collected on an Oxford Diffraction Gemini single crystal diffractometer at 150 K. The data was processed using the Oxford Diffraction software package (CrysAlisPro Version 1.171.33.55). The data for $\mathbf{3}_{S\text{-}S}$ and **4** was collected at Diamond using Synchrotron radiation (λ = 0.68890 Å) on a CrystalLogic Kappa (3 circle), Rigaku Saturn724 at 100 K. Data was processed using the Rikagu software package (CrystalClear-SM Expert 2.0 r5).

For most structures a symmetry-related (multi-scan) absorption correction had been applied. The structures were solved by direct methods using the programmes SIR97[2] or SHELXS-97[3] followed by full-matrix least squares refinement on F^2 using SHELXL-97[3] implemented in the WINGX-1.80 suite of programmes throughout. Additional programmes used for analysing and graphically handling data included: SHELXle,[4] PLATON,[5] and ORTEP 3 for

Windows. Hydrogen atoms were placed onto calculated positions and isotropically refined using a riding model.

1.2 HPLC methods

Method A was carried out using a Symmetry® C-18 column (4.6 x 260 mm) with UV/visible detection measured at λ_{obs} = 200 nm, 300 nm, 400 nm, 450 nm, 500 nm, 600 nm, 700 nm and 800 nm. The gradient elution was 1.1 mL/minute, with 0.1% TFA MilliQ water as solvent A and 0.1% TFA MeCN as solvent B. Start 95 % A reverse gradient until 5% A at 7.5 minutes, isocratic until 15 minutes, reverse gradient until 17.5 minutes 95% A, then hold to 18 minutes.

Method B was carried out using a Symmetry® C-18 column (4.6 x 260 mm) with UV/visible detection measured at eight wavelengths from λ_{obs} = 200 nm, 220 nm, 280 nm, 300 nm, 400 nm, 450 nm, 500 nm, 600 nm, 700 nm and 800 nm. The gradient elution was 0.8 mL/minute, with 0.1% TFA MilliQ water as solvent A and 0.1% TFA MeCN as solvent B. Start 95 % A reverse gradient until 5% A at 7.5 minutes, isocratic until 15 minutes, reverse gradient until 17.5 minutes 95% A, then hold to 18 minutes.

Method C was carried out using a Acclaim® 120 C-18 column (4.6 x 150 mm) with UV/visible detection measured at up to four λ_{obs} = 214 nm, 220 nm, 254 nm, 280 nm, 300 nm and 400 nm. The gradient elution was 1.0 mL/minute, with 0.1% TFA MilliQ water as solvent A and 0.1% TFA MeCN as solvent B. Start 95 % A reverse gradient until 5% A at 7.5 minutes, isocratic until 15 minutes, reverse gradient from 15.1 minutes 95% A, then hold to 18 minutes.

Method D was carried out using a Waters C-18 column (4.6 x 250 mm) with UV/visible detection measured at up to four λ_{obs} = 254 nm. The gradient elution was 1.0 mL/minute, with 0.1% TFA MilliQ water as solvent A and 0.1% TFA MeCN as solvent B. Start 95 % A reverse gradient until 5% A at 12 minutes, isocratic until 15 minutes, reverse gradient from 15.1 minutes 95% A, then hold until 21 minutes.

Method E was carried out using a Acclaim® 120 C-18 column (4.6 x 150 mm) with UV/visible detection measured at up to four λ_{obs} = 214 nm, 220 nm, 254 nm, 280 nm, 300 nm

and 400 nm. The gradient elution was 1.0 mL/minute, with 0.1% TFA MilliQ water as solvent A and 0.1% TFA MeCN as solvent B. Start 95 % A reverse gradient until 5% A at 10 minutes, isocratic until 15 minutes, reverse gradient from 15.1 minutes 95% A, then hold to 18 minutes.

Method F was carried out using a Waters C-18 column (4.6 x 250 mm) with UV/visible detection measured at λ_{obs} = 254 nm. The gradient elution was 1.0 mL/minute, with 0.1% TFA MilliQ water as solvent A and 0.1% TFA MeCN as solvent B. Start 95 % A, reverse gradient until 5% A at 10 minutes, isocratic until 12 minutes, reverse gradient until 95% A at 14 minutes, then hold to 15 minutes at 95% A.

Method G was carried out using a Waters C-18 column (4.6 x 250 mm) with UV/visible detection measured at up to four λ_{obs} = 214 nm, 220 nm, 254 nm, 280 nm, 300 nm and 400 nm. The gradient elution was 1.0 mL/minute, with 0.1% TFA MilliQ water as solvent A and 0.1% TFA MeCN as solvent B. Start 95 % A reverse gradient until 5% A at 15 minutes, isocratic until 22.5 minutes, reverse gradient from 22.6 minutes 95% A, then hold to 25.5 minutes.

Method H was carried out using an Eclipse C-18 column (2 x 50 mm) with UV/visible detection measured at up to four λ_{obs} = 214 nm, 220 nm, 254 nm, 280 nm, 300 nm and 400 nm. The gradient elution was 1.0 mL/minute, with 0.1% TFA MilliQ water as solvent A and 0.1% TFA MeOH as solvent B. Start 95 % A reverse gradient until 5% A at 8 minutes, hold until 10 minutes.

Method I was carried out using a Phenomenex Ultracarb C-18 column (4.6 x 150 mm) with UV/visible detection measured at up to four λ_{obs} = 254 nm. The gradient elution was 1.0 mL/minute, with 0.1% TFA MilliQ water as solvent A and 0.1% TFA MeOH as solvent B. Start 95 % A reverse gradient until 5% A at 12 minutes, hold until 15 minutes.

Semi-Preparative HPLC was carried out using a Phenomenex® Gemini C-18 column (10 x 250 mm) with UV/visible detection measured at up to four λ_{obs} = 214 nm, 220 nm, 254 nm, 280 nm, 300 nm and 400 nm. The gradient elution was 1.0 mL/minute, with 0.1% TFA MilliQ water as solvent A and 0.1% TFA MeCN as solvent B. Start 95 % A reverse gradient

until 5% A at 15 minutes, isocratic until 22.5 minutes, reverse gradient from 22.6 minutes 95% A, then hold to 25.5 minutes.

1.3 General Radiochemistry Procedures

The zinc complex was prepared as either 1 mg/mL or 2 mg/mL in DMSO. Gallium experiments were first carried at MSKCC, and the protocols repeated and optimised out at Hammersmith Hospital, Imperial College London. In the optimised procedures, 10 mL of 0.1 M HCl was used to elute ca. 222 MBq of $^{68}Ga^{3+}$ from the generator and was subsequently trapped on a 30 mg/mL Strata X-C cartridge. This was eluted with 700 µL of 0.02 M HCL / 98% acetone and dried for 15 minutes under a stream of nitrogen at 110°C. Next, 25 µL of 2 mg/mL zinc complex in DMSO and 2 mL of HPLC-grade ethanol. The solution was heated for 30 minutes at 90 °C. HPLC methods F, G, H and I were used in all cases.

1.4 Preliminary In vivo imaging

All experiments were carried out following an Institutional Animal Care and Use Committee-approved protocol, Memorial Sloan-Kettering Cancer Center, New York within the laboratory of Prof Jason Lewis. Athymic mice aging six to eight weeks were obtained from Taconic Farms Incorporated (Hudson, NY). The animals were given ca. 1 week prior to treatment to acclimatise in ventilated cages and were provided with food and water *ad libitum*. Xenografts were induced on the right shoulder by subcutaneous injection of 100 µL of 3 x 10^6 PC-3 cells.

Small-animal PET imaging was performed at MSKCC, New York, on a micro-PET R4 rodent scanner (Concorde Microsystems) by Dr Nerissa Vilega. Mice were injected with complex radiolabelled with Ga-68 of 10-15 MBq and anesthetised by inhalation of 2% isoflurane (Baxter Healthcare, Deerfield, IL)/oxygen gas mixture. The animal was positioned on the scanner bed with anaesthesia maintained by a 1% isoflurane/gas mixture. Measurements for each mouse were recorded using static scans at time points between 30 minutes and 24 hours. Each scan occurred for between 10 and 45 minutes and contained at least 20 million coincident events. The energy window used was 350-700 keV, with a coincidence timing window of 6 ns. Data were arranged into 2D histograms by Fourier rebinning, and transverse images were reconstructed by filtered back-projection into a 128 x 128 x 63 (0.72 x 0.72 x 1.3 mm^3) matrix. Images were normalised to correct for non-uniformity of response of the PET, dead-time count losses, positron branching ratio, and

physical decay to the time of injection but no attenuation, scatter, or partial-volume averaging correction was applied. The counting rates of the reconstructed images were converted to activity concentrations (percentage of injected dose [%ID] per gram of tissue) using a calibration factor based on the image of a mouse-sized water-equivalent phantom containing Ga-68. ASIPro VM™ software (Concorde Microsystems) was used to analyse the data.

1.5 pH and biomimetic media studies

Buffers from pH 1.1 to pH 10 were made using a Fisher brand Hydrus 600 pH meter in order to investigate the stability of compounds by fluorescence spectroscopy. The following buffer systems were used, following the procedure described in "Buffers: A guide for the preparation and use of buffers in biological systems"; Chandra Mohan, 1997.[6]

pH Buffer systems:
1.1 KCl/HCl
2.0 KCl/HCl
3.0 Citric acid/Sodium citrate dehydrate OR Glycine / HCl
5.0 Citric acid/Sodium citrate dehydrate OR Acetic Acid / Sodium acetate
7.0 Citric acid/Dibasic Sodium phosphate OR Sodium phosphate Monobasic / Sodium phosphate Dibasic
9.0 Glycine/Sodium Hydroxide
10.0 Sodium Carbonate Anhydrous/Sodium Hydrogen Carbonate

A 4 mL solution of twice the final concentration was added to 4 mL of the buffer system or biomimetic media and then scanned for fluorescence between 200-800 nm with an excitation wavelength of λ_{max}. If precipitation was observed samples were filtered using a Millex™ 0.22 μm filter prior to scanning. Eagle's Minimum Essential Medium containing 0.5% penicillin/streptomycin and 1% glutamine was used to assess the stability of the compounds in biomimetic media and also in relevance to the cell studies. Two equivalents of biologically relevant agents glutathione (GSH), L-cysteine, L-histidine, L-methionine, citric acid or chelator ethylenediaminetetraacetic acid (EDTA) were used.

1.6 Cell culturing and cell plate preparation

Cells were cultured at 37 °C in a humidified atmosphere in air and diluted once confluence had been reached. Culture occurred in Eagle's Minimum Essential Medium (EMEM) for

HeLa (cervical cancer cells) and FEK-4 (epithelial fibroblast cells), Dulbecco's Modified Eagle's Medium (DMEM) for MCF-7, RPMI 1640 for PC3 (prostate cancer cells) and Weymouth's medium for EMT6 (murine breast carcinoma). The media contained foetal calf serum (FCS) (10% for HeLa, PC-3, EMT6 and MCF-7 and 15% for FEK-4), 0.5% penicillin/streptomycin (10,000 IU mL^{-1}/10,000 mg mL^{-1}) and 200 mM L-Glutamine (5 mL). All steps were performed in absence of phenol red. Surplus supernatant containing dead cell matter and excess protein was aspirated. The live adherent cells were then washed with 2 x 10 mL aliquots of phosphate buffer saline (PBS) solution to remove any remaining media containing FCS, which inactivates trypsin. Cells were resuspended in solution by incubation in 3 mL of trypsin–PBS solution (0.25% trypsin) for 5 min at 37 °C. After trypsinisation, 5 mL of medium containing 10% serum was added to inactivate the trypsin and the solution was centrifuged for 5 min (1000 rpm, 25 ºC) to remove any remaining dead cell matter. The supernatant liquid was aspirated and 5 mL of cell medium (10% or 15% FCS) was added to the cell matter left behind. Cells were counted using a haemocytometer and then seeded as appropriate.

1.6.1 MTT assays.

Cells cultured as above were plated (5 x 10^4 cells mL^{-1}) and left for 48 h to adhere fully. All steps were carried out in the absence of phenol red. (a) For MI_{50} estimations by MTT assays, cells were incubated with each compound tested for 48 h at 37 °C. Concentrations used were 1 nM, 100 nM, 1 µM, 10 µM, 50 µM, 100 µM and 250 µM (1% DMSO, 99% Eagle's Modified Essential Medium containing FCS at standard concentration of the cell line). Subsequently, cells were washed three times with PBS and 100 mL of MTT was added (0.5 mg mL^{-1}, 10% PBS: SFM) followed by a 2 h incubation. Following aspiration, 100 mL of DMSO was added and 96 well plates were read at an ELISA plate reader, Molecular Devices Versa Max (BN02877). Data were obtained from five consistent results and MI_{50} was calculated using Origin 8 as half the height of the fitted curve for each compound and for each individual experiment. Due to background absorbance, 100% cell death would not correspond to zero absorbance, therefore the height of the curve was calculated as the highest absorbance of the fit plot minus the minimum absorbance of the curve, at which point death of all cells has been achieved. Where 100% cell death had not been attained, the MI_{50} was calculated using the same method in that the minimum absorbance of the fitted curve was subtracted. This value therefore indicates the minimum MI_{50} for this compound and is stated in the text/figure legend. The mean MI_{50} was calculated from the average of the five MI_{50}

values obtained from five consistent experiments. The error reported was the standard error of the mean and shown as ± S.E.M.

Time lapse studies by MTT assays were carried out in HeLa, MCF-7 and FEK-4 cells by an analogous method to that described above, but under serum free EMEM conditions aiming to link directly cytotoxicity tests to fluorescence imaging experiments. Concentrations used were 10 μM and 50 μM in 1% DMSO, 99% Eagle's Modified Essential Medium. Conclusions were reached following observations of cells' viability within 1 h incubation (37 °C) interval.

1.6.2 LDH assays

The medium containing serum was aspirated and the compounds were added in serum free medium (SFM). After drug treatment, the supernatant was removed and added to a separate 96 well plate. To the remaining cells 100 μL SFM containing SDS was added. A preliminary study showed that the optimum percentage of SDS for FEK-4 and MCF-7 cells was 0.015% and for HeLa cells 0.1 %. These were mixed by pipette and 20 μL of LDH reaction kit, Roche Scientific ® (1:40 catalyst:dye) was added to each well. After 20 min the 96 well plates were read by Molecular Devices Versa Max (BN02877) at 570 nm. % LDH leakage was calculated by supernatant OD divided by (supernatant OD + cell OD).

1.7 Fluorescence microscopy in cells.

At Bath fluorescence microscopy experiments were carried out on a Nikon eclipse TE2000 epi-fluorescence or a Zeiss LSM510META microscope, whereas at the Rutherford Appleton Laboratory a modified Nikon TE2000-U microscope was used. For all fluorescence microscopy experiments, cells were cultured as above and plated in glass-bottomed dishes as 1.5 x 10^5 cells per dish (*ca.* 60% coverage) and incubated for 12 h for HeLa, EMT-6 and FEK-4, 48 h for MCF-7 and 72 h for PC-3 cells. All steps were carried out in the absence of phenol red. This timing was found to be minimum necessary for the two different cell lines to adhere to the glass bottomed Petri dishes used for imaging. Prior to compound addition, cells were washed 3 times with PBS, before adding serum free medium (2 mL). Subsequently, a small volume of medium was removed and compound in DMSO was added to obtain a final volume of 1 mL and the desired concentration. The final concentration of compounds on the cell plate was 50 mM in medium, containing 0.5% DMSO or 1% DMSO, depending on the compound solubility. After 20 min or 1 h incubation with the compound cells were washed 3

times with PBS and fresh serum free medium was added (1 mL) and images were recorded immediately.

1.8 Co-localisation studies.

Cells were cultured using standard protocols, as described above, in RPMI-1640 (PC-3). Prior to addition of any commercial co-localisation dye, cells were washed 5 times with PBS. Protocols adapted from *Invitrogen* were used throughout.

(*a*) *Hoechst.* A stock solution of 100 mg mL^{-1} Hoechst 33342 (*Invitrogen*) was prepared in sterile MilliQ water. To 10 µL of the stock solution, a volume of 990 µL was added giving a final concentration of 1 mg mL^{-1}. After cells were incubated with this solution for 30 min a further 5 washings with PBS were carried out, before the addition of 1 mL of SFM. Subsequently, a small volume of serum free medium (SFM) was removed (e.g. 10 µL) and compound in DMSO was added in equal volume to what was removed, to obtain a final volume of 1 mL and the desired concentration (50 mM in cell medium containing 0.5% DMSO). After 20 min or 1 h incubation with the compound cells were washed 3 times with PBS and fresh serum free medium was added (1 mL) and images were recorded immediately.

(*b*) *Mitotracker.* A stock solution of 20 µM Mitotracker® Red FM (*Invitrogen*) was prepared in DMSO. To 10 µL of stock solution, 990 µL was added giving a final concentration of 200 nM. After cells were incubated with this solution for 30 min a further 5 washings with PBS were carried out, before the addition of 1 mL of SFM. Subsequently, a small volume of SFM was removed (e.g. 10 µL) and compound in DMSO was added in equal volume to what was removed, to obtain a final volume of 2 mL and the desired concentration (50 mM in cell medium containing 0.5% DMSO). After 20 min or 1 h incubation with the compound cells were washed 3 times with PBS and fresh serum free medium was added (1 mL) and images were recorded immediately.

(*c*) *Lysotracker* A stock solution of 20 µM Lysotracker® Red DND-99 (*Invitrogen*) was prepared in DMSO. To 10 µL of stock solution, 990 µL was added giving a final concentration of 200 nM. After cells were incubated with this solution for 60 min a further 3 washings with PBS were carried out, before the addition of 1 mL of SFM. Subsequently, a small volume of SFM was removed (e.g. 10 µL) and compound in DMSO was added in equal volume to that which was removed to obtain a final volume of 1 mL and the desired

concentration (50 mM in cell medium containing 0.5% DMSO). After 20 min or 1 h incubation with the compound cells were washed 3 times with PBS and fresh serum free medium was added (1 mL) and images were recorded immediately.

(d) ER tracker

A stock solution of 100 μM ER-tracker® Red (*Invitrogen*) was prepared in DMSO. To 10 μL of stock solution, 990 μL was added giving a final concentration of 100 μM. After cells were incubated with this solution for 20 min a further 3 washings with HBSS were carried out, before the addition of 1 mL of SFM. Subsequently, a small volume of SFM was removed (e.g. 10 μL) and compound in DMSO was added in equal volume to that which was removed to obtain a final volume of 1 mL and the desired concentration (e.g. 50 mM in cell medium containing 0.5% DMSO). After 20 min or 1 h incubation with the compound cells were washed 3 times with PBS and fresh serum free medium was added (1 mL) and images were recorded immediately.

(e) Nile Red

A stock solution of 10 μg/mL Nile Red (*Invitrogen*) was prepared in DMSO. To 10 μL of stock solution, 990 μL was added giving a final concentration of 100 ng/mL. After cells were incubated with this solution for 10 min a further 3 washings with HBSS were carried out, before the addition of 1 mL of SFM. Subsequently, a small volume of SFM was removed (e.g. 10 μL) and compound in DMSO was added in equal volume to that which was removed to obtain a final volume of 1 mL and the desired concentration (e.g. 50 mM in cell medium containing 0.5% DMSO). After 20 min or 1 h incubation with the compound cells were washed 3 times with PBS and fresh serum free medium was added (1 mL) and images were recorded immediately.

(f) Alexa Fluor

A stock solution of 50 μg/mL Alexa fluor® 350 conjugate (*Invitrogen*) was prepared in HBSS (Hank's Buffered Salt Solution). To 10 μL of stock solution, 990 μL was added giving a final concentration of 100 μM. After cells were incubated with this solution for 10 min a further 3 washings with HBSS were carried out, before the addition of 1 mL of SFM. Subsequently, a small volume of SFM was removed (e.g. 10 μL) and compound in DMSO was added in equal volume to that which was removed to obtain a final volume of 1 mL and

the desired concentration (e.g. 50 mM in cell medium containing 0.5% DMSO). After 20 min or 1 h incubation with the compound cells were washed 3 times with PBS and fresh serum free medium was added (1 mL) and images were recorded immediately.

1.9 Fixed cell imaging

Cells were plated in a Petri dish containing a glass cover slip. Before fluorescence imaging, the serum containing medium was replaced with SFM. After the drug treatment cells were fixed by inverting the coverslip placing it onto 50 μL of paraformaldehyde-PBS (4% paraformaldehyde). After 15 min, the coverslip was mounted to a slide using Vectashield®, hard set mounting medium with DAPI (Vector Laboratories, Inc.)

1.10 Two-photon excitation experiments

Mira titanium sapphire laser (Coherent Lasers Ltd), generating 180 fs pulses at 75 MHz and emitting light at a wavelength of 580-630nm nm. The laser was pumped by a solid state continuous wave 532 nm laser (Verdi V18, Coherent Laser Ltd), with the oscillator fundamental output of 915 ± 2 nm or 810 ± 2 nm. The laser beam was focused to a diffraction limited spot through a water immersion ultraviolet corrected objective (Nikon VC x60, NA1.2) and specimens illuminated at the microscope stage of a modified Nikon TE2000-U with UV transmitting optics.

The focused laser spot was raster scanned using an XY galvanometer (GSI Lumonics). Fluorescence emission was collected without de-scanning, bypassing the scanning system and passed through a coloured glass (BG39) filter. The scan was operated in normal mode and line, frame and pixel clock signals were generated and synchronised with an external fast microchannel plate photomultiplier tube used as the detector (R3809-U, Hamamatsu, Japan). These were linked *via* a Time-Correlated Single Photon Counting (TCSPC) PC module SPC830. Lifetime calculations were obtained using SPCImage analysis software (Becker and Hickl, Germany) or Edinburgh Instruments F900 TCSPC analysis software. Preliminary single-photon FLIM investigations were conducted using the Becker and HicklDCS120 system with a 40 ps 473 nm diode laser.

1.11 In vitro hypoxia testing study

Cells were cultured as described above with the final concentration on the cell plate 50 μM, containing 4% DMSO for 20 minutes. Normoxic conditions were 20.7% O_2 and 5% CO_2 at

37 °C, with hypoxic samples pre-incubated for 20 minutes at 1% O_2, 5% CO_2 at 37 °C before complex addition. Cells were washed 3 times with PBS, which was replaced with 2 mL of serum free media, with images recorded immediately.

1.12 Flow cytometry studies

Cells were seeded as 3 x10^5 cells per well in a 6 well plate and incubated for ca. 12 h.
The cells were subsequently washed twice with PBS, with the media replaced with serum free medium and compound of 50 μM 4%, DMSO final concentration. This was incubated for 20 minutes at 20.7% O_2 and 5% CO_2 at 37 ˚C for the normoxic sample. Hypoxic conditions were obtained by pre-incubating the cells for 20 minutes at 1% O_2 and 5% CO_2 at 37 ˚C, followed by incubation of a further 20 minutes under the same conditions with the compound. Following this cells were washed three times with PBS, trypsinised and centrifuged at 600 g for three minutes. The precipitate was washed with PBS, resuspended in 1 mL of serum free medium, kept on ice and analysed in an LSRII cytometer (BD Biosciences, Rockville, MD USA), with 10 000 cells counted per event. Each experiment was carried out at least three times, with data analysis performed using FlowJo software (TreeStar, USA).

1.13 Radioactive cell uptake investigation

Cells were seeded as 3 x10^5 cells per well in a 6 well plate and incubated for ca. 12 h.
The cell medium was aspirated and replaced with serum free medium containing the Ga-68 radiolabelled complex (following the radiochemical procedure above). This was incubated at 20.7% O_2 and 5% CO_2 at 37˚C for the normoxic sample. Hypoxic conditions were obtained by pre-incubating the cells for 20 minutes at 1% O_2 and 5% CO_2 at 37 ˚C, followed by incubation under the same conditions with the compound for the time course of the study. Cell plates were put on ice, washed 3 times with ice-cold PBS and lysed using 0.2 mL RIPA buffer for 10 minutes (Thermo Fisher Scientific Inc., Rockford, IL, USA). PBS (0.5 mL) was added to each well and cell lysates were transferred to counting tubes, with measurements of decay-corrected radioactivity performed using a gamma counter (Cobra II Auto-Gamma counter, Packard Biosciences Co, Pangbourne, UK). Aliquots were snap-frozen and subsequently protein determination was carried out using a BCA 96-well plate assay (Thermo Fisher Scientific Inc., Rockford, IL, USA). Decay corrected counts were corrected to protein concentration, with data presented as percent of total radioactivity per mg of protein,

1.14 Statistical analysis

Data were expressed as mean ± standard error of the mean (SEM), unless stated in the text. Student's *t* test (Prism v5.0 software for windows, GraphPad Software, San Diego, CA, USA) was used to assess the significance of comparison between two data sets. If $P \leq 0.05$ the differences between groups were regarded as significant.

2. Synthesis of the ligands

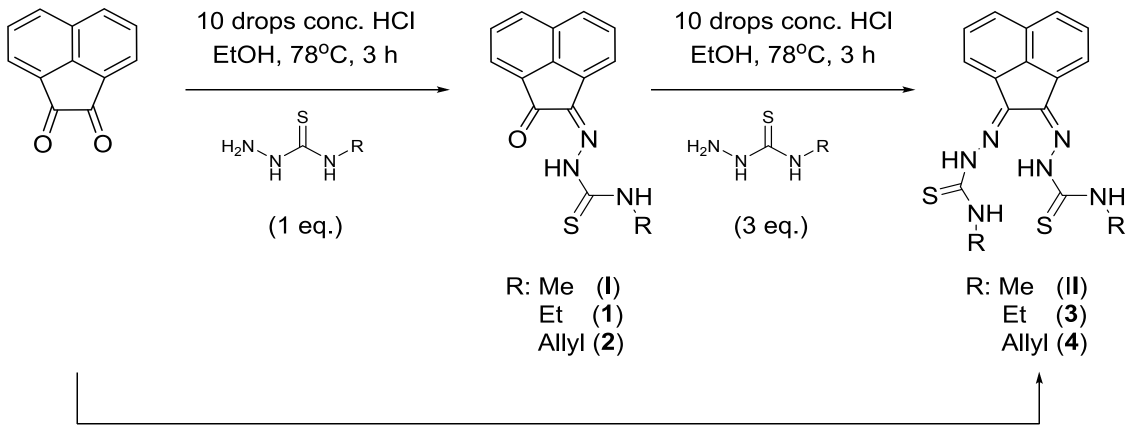

Scheme S.1. General scheme for the synthesis of mono and *bis*(substituted) thiosemicarbazones.

Microwave reactions were performed in a Biotage Initiator 2.5 system in 5 mL glass capped vials. The reaction mixture was prestirred for 30 s and then heated for the selected time. If the irradiation power is not set, it reaches its maximum (300 W from magnetron at 2.45 GHz) at the start of the reaction until the target temperature is reached, decreasing to lower values afterwards. The temperature of the reaction is controlled by an IR sensor and the vial cooled down by an air stream when the reaction has finished.

General method for microwave synthesis, A: acenaphthenequinone (0.200 g, 1.04 mmol) and the corresponding thiosemicarbazide (1.04 mmol) were suspended in 5-6 mL of ethanol in a microwave vial. Then, 3 drops of conc. HCl added were added and the vial was sealed. The reaction mixture was prestirred for 30 s and then heated to 90 ºC for 9 min. The yellow solid was filtered whilst hot, resuspended in hot methanol, stirred for 15 min, washed with methanol and diethyl ether and dried under vacuum.

General method for microwave synthesis, B: acenaphthenequinone (0.200 g, 1.04 mmol) and the corresponding thiosemicarbazide (3.13 mmol) were suspended in 5-6 mL of ethanol in a microwave vial. Then, 3 drops of conc. HCl added were added and the vial was sealed. The reaction mixture was prestirred for 30 s and then heated to 90 ºC for 9 min. The yellow solid was filtered whilst hot, resuspended in hot methanol, stirred for 15 min, washed with methanol and diethyl ether and dried under vacuum.

2.1 Mono(4-methyl-3-thiosemicarbazone) acenaphthenequinone (I)

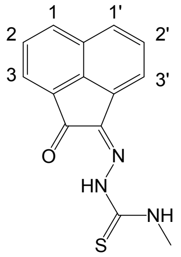

For Compound **I**, acenaphthenequinone (0.50 g, 2.74 mmol) and 4-methyl-3-thiosemicarbazide (0.29 g, 2.80 mmol) were added in absolute ethanol (15 mL) and refluxed for 2 hours. 10 drops of conc. HCl were added upon reflux. The solid was isolated by filtration whilst hot, resuspended in hot methanol (10 mL) and stirred for 15 minutes before filtering and washing with further methanol. The resultant yellow solid (0.60 g, 2.25 mmol, 82%), was dried under vacuum.

Microwave synthesis: carried out according to general method A, (0.199 g, 71 %)

^{1}H NMR (300 MHz, d_6–DMSO, 25 °C): δ 12.65 (s, 1H, N-N*H*), 9.38 (m, 1H, N*H*Me), 8.38 (d, 1H, *H-3*, *J* = 8.3 Hz), 8.14 (d, 1H, *H-3'*, *J* = 8.3 Hz), 8.10 (d, 1H, *H-1*, *J* = 7.1 Hz), 7.98 (d, 1H, *H-1'*, *J* = 6.9 Hz), 7.88 (overlapping t, 2H, *H-2*), 7.85 (overlapping t, 2H, *H-2'*), 3.13 (d, 3H, (C*H$_3$*)). **^{13}C NMR** (75.5 MHz, d_6–DMSO, 25°C): δ 188.6, 178.0, 139.1, 137.2, 132.9, 130.5, 130.1, 130.0, 129.0, 128.7, 127.2, 122.6, 118.3, 31.5. **Mass spectrum** ESI-MS calcd for $C_{14}H_{12}N_3OS^+$ $[M + Na]^+$ 292.0515, found m/z = 292.0494.

2.2 Mono(4-ethyl-3-thiosemicarbazone) acenaphthenequinone (1)

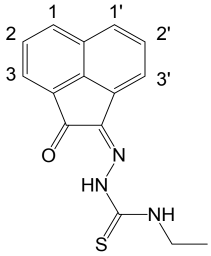

For Compound **1**, acenaphthenequinone (0.50 g, 2.74 mmol) and 4-ethyl-3-thiosemicarbazide (0.36 g, 3.00 mmol) were added in absolute ethanol (15 mL) and refluxed for 2 hours. 10 drops of conc. HCl were added upon reflux. The solid was isolated by filtration whilst hot, resuspended in hot methanol (10mL) and stirred for 15 minutes before filtering and washing with further methanol. The resultant yellow solid (0.66 g, 2.33 mmol, 85%), was dried under vacuum.

Microwave synthesis: carried out according to general method A, (0.2165 g, 73 %)

^{1}**H NMR** (300 MHz, *d$_6$*–DMSO, 25°C): δ 12.60 (s, 1H, N-N*H*), 9.42 (t, 1H,N*H*Et, *J* = 5.9 Hz), 8.38 (d, 1H, *H-3*, *J* = 8.2 Hz), 8.14 (d, 1H, *H-3'*, *J* = 8.3 Hz), 8.10 (d, 1H, *H-1*, *J* = 7.0 Hz), 8.01 (d, 1H, *H-1'*, *J* = 6.9 Hz), 7.88 (t+t,2H, *H-2* and *H-2'*), 3.68 (m, 2H, (C*H$_2$*)CH_3), 1.23 (t, 3H, CH_2C*H$_3$*, *J* = 7.2 Hz). ^{13}**C NMR** (75.5 MHz, *d*$_6$ –DMSO, 25 °C): δ 188.5, 176.9, 139.1, 137.2, 132.8, 130.5, 130.1, 129.9, 128.9, 128.6, 127.1, 122.5, 118.3, 39.1, 14.1. **Mass spectrum** ESI-MS calcd for $C_{15}H_{14}N_3OS^+$ $[M + Na]^+$ 306.0677, found 306.0667.

2.3 Mono(4-allyl-3-thiosemicarbazone) acenaphthenequinone (2)

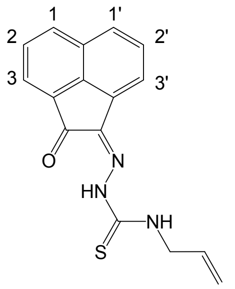

For Compound **2**, acenaphthenequinone (0.500 g, 2.745 mmol) and 4-allyl-3-thiosemicarbazide (0.393 g, 3.293 mmol) were added in absolute ethanol (15mL) and refluxed for 2 hours. 10 drops of conc. HCl were added upon reflux. The solid was isolated by filtration whilst hot, resuspended in hot methanol (10mL) and stirred for 15 minutes before filtering and washing with further methanol. The resultant yellow solid (0.595 g, 1.723 mmol, 63%), compound **2**, was dried under vacuum.

Microwave synthesis: carried out according to general method A, (0.2372 g, 77 %)

^{1}**H NMR** (*d$_6$*-DMSO, 25 °C): δ 12.61 (s, 1H, N-N*H'*), 9.57 (t, 1H, N*H*Allyl, *J* = 6.0 Hz), 8.34 (d, 1H, *H-3*, *J* = 8.2 Hz), 8.11 (d, 1H, *H-3'*, *J* = 8.3 Hz), 8.06 (d, 1H, *H-1'*, *J* = 7.0 Hz), 7.96 (d, 1H, *H-1*, *J* = 6.9 Hz), 7.85 (overlapping t, 1H, *H-2* or *H-2'*), 7.81 (overlapping t, 1H, *H-2* or *H-2'*), 5.95 (m, 1H, -CH_2C*H*CH_2), 5.27 + 5.21 (q, 1H, H_{trans}, *J* = 17.3 Hz), 5.19 + 5.15 (q, 1H, H_{cis}, *J* = 10.3 Hz), 4.30 (ddt, 2H, -C*H$_2$*$CHCH_2$). ^{13}**C NMR** (*d$_6$*-DMSO, 25 ◦C): δ 188.5, 177.5, 139.1, 137.3, 134.0, 132.8, 130.4, 130.0, 129.9, 128.9, 128.6, 127.1, 122.5, 118.4, 116.3, 46.5. **Mass spectrum** ESI-MS calcd for $C_{16}H_{13}N_3NaOS^+$ 318.0677 $[M+Na]^+$, found 318.0665. **Elem. Anal.**: Found: C; 65.09 %, H; 4.43 %, N; 14.21 %, Calc.: C; 65.06 %, H; 4.44 %, N; 14.23 %.

2.4 Bis(4-methyl-3-thiosemicarbazone) acenaphthenequinone (II)

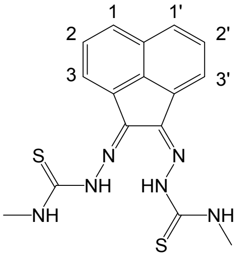

Acenaphthenequinone (0.25g, 0.137 mmol) and 4-methyl-thiosemicarbazide (0.43 g, 0.411 mmol) were suspended in 40 mL ethanol and refluxed for 4 hours. 10 drops of conc. HCl were added upon reflux. The solid was isolated by filtration whilst hot, resuspended in hot methanol (10 mL) and stirred for 15 minutes before filtering and washing with further methanol. The resultant yellow solid (266.6 mg, 54%) was dried under vacuum.
Microwave synthesis: carried out according to general method B, (0.353 g, 95 %)

^{1}H NMR (300 MHz, *d$_6$*-DMSO, 25ºC) δ 12.60 (s, 1H, N-N*H'*), 11.29 (s, 1H, N-N*H*), 9.15 (m, 1H, N*H'*CH_3), 8.86 (m, 1H, N*H*CH_3), 8.20 (d 1H, *H-1*, *J* = 7.3 Hz), 8.13 (d, 1H, *H-3'*, *J* = 8.3 Hz), 8.07 (d, 1H, *H-1'*, *J* = 7.0 Hz), 8.03 (d, 1H, *H-3*, *J* = 8.3 Hz), 7.79 (m, 2H, *H-2* and *H-2'*), 3.11 (m, 6H, overlapping CH_3 groups).**^{13}C NMR**- (75.5 MHz, *d$_6$*-DMSO, 25ºC) δ 178.42, 138, 136.45, 133.23, 130.25, 129.20, 128.20, 128.18, 127.03, 125.02, 31.79, 31.6. **Mass spectrum** ESI-MS calcd for $C_{16}H_{15}N_6S_2^-$ $[M-H]^-$ 355.0800, found 355.0804 **Elem. Anal.**: Found C; 53.7 %, H, 4.51 %, N; 23.4 %, Calc.: C; 53.91 %, H; 4.52 %, N; 23.58 %.

2.5 Bis(4-ethyl-3-thiosemicarbazone) acenaphthenequinone (3)

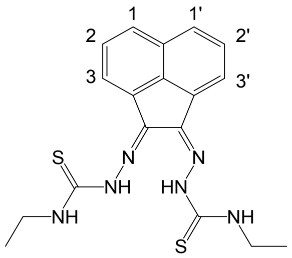

Acenenaphthenequinone (0.25 g, 0.137 mmol) and 4-ethyl-3thiosemicarbazide (0.48 g, 0.411 mmol) were suspended in 40 mL ethanol and refluxed for 4 hours. 10 drops of conc. HCl were added upon reflux. The solid was isolated by filtration whilst hot, resuspended in hot methanol (10 mL) and stirred for 15 minutes before filtering and washing with further methanol. The resultant yellow solid (446.7mg, 85%) was dried under vacuum.
Microwave synthesis: carried out according to general method B, (0.359 g, 90%)

^{1}**H NMR** (300 MHz, d_6-DMSO, 25ºC) 12.55 (s, 1H, N-N*H'*), 11.21 (s, 1H, N-N*H*), 9.19 (t, 1H, N*H'*Et, *J* = 6.0 Hz), 8.80 (t, 1H, N*H*Et, *J* = 5.5 Hz), 8.19 (d, 1H, *H-1*, *J* = 7.2 Hz), 8.13 (d, 1H, *H-3'*, *J* = 8.3 Hz), 8.09 (d, 1H, *H-1'*, *J* = 7.0 Hz), 8.03 (d, 1H, *H-3*, *J* = 8.3 Hz), 7.79 (overlapping t, 2H, *H-2* and *H-2'*), 3.66 (m, 4H, CH_2CH_3), 1.24 (m, 6H, CH_2CH_3). ^{13}**C NMR**- (75.5 MHz, d_6-DMSO, 25ºC) δ 178.77, 176.92, 139.25, 139.16, 136.66, 133.06, 130.04, 128.69, 128.58, 128.12, 128.35, 126.17, 124.64, 119.60, 31.05 **Mass spectrum** ESI-MS calcd for $C_{18}H_{19}N_6S_2$ [M-H]$^-$ 383.1113, found 383.1155. **Elem. Anal.**: Found C; 56.1 %, H, 5.23 %, N; 21.8 %. Calc.: C; 56.22 %, H; 5.24 %, N; 21.86 %.

2.6 Bis(4-allyl-3-thiosemicarbazone) acenaphthenequinone (4)

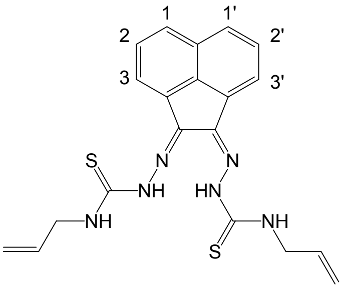

Acenaphthenequinone (0.25g, 0.137mmol) and 4-allyl-3-thiosemicarbazide (0.54 g, 0.411 mmol) were suspended in 40 mL ethanol and were refluxed for 4 hours. 10 drops of conc. HCl were added upon reflux. The solid was isolated by filtration whilst hot, resuspended in hot methanol (10 mL) and stirred for 15 minutes before filtering and washing with further methanol. The resultant yellow solid (415 mg, 74%), was dried under vacuum.

Microwave synthesis: carried out according to general method B, (0.402 g, 94 %)

^{1}**H NMR**: (300 MHz, d_6-DMSO, 25ºC) δ 12.63 (s,1H, N-N*H'*), 11.27 (s,1H, N-N*H*), 9.32 (t, 1H, N*H*Allyl, *J* = 6.0 Hz), 9.02 (t, 1H, N*H'*Allyl, *J* = 5.7 Hz), 8.21 (d, 1H, *H-1* or *H-1'*, *J* = 7.2 Hz), 8.11 (overlapping d, 2H, *H-3* and *H-3'*), 8.01 (d, 1H, *H-1* or *H-1'*, *J* = 7.1 Hz), 7.80 (overlapping t, 1H, *H-2* or *H-2'*), 7.78 (overlapping t, 1H, *H-2* or *H-2'*), 5.95 (m, 2H, CH_2CHCH_2) 5.35 + 529 (ddt, 2H, H_{trans}, *J* = 17.4 Hz), 5.25 + 5.18 (ddt, 2H, H_{trans}, *J* = 17.4 Hz), 5.17 + 5.14 (m, 2H, H_{cis}, *J* = 10.6 Hz), 4.31 (m, 4H, CH_2CHCH_2), . ^{13}**C NMR**: (75.5 MHz, d_6-DMSO, 25ºC) δ 182.52, 178.14, 138.23, 136.45, 134.86, 134.40, 133.21, 130.32, 129.28, 128.88, 128.55, 128.18, 127.09, 124.93, 116.83, 116.28, 47.07, 46.52. **Mass spectrum** ESI-MS calcd for $C_{20}H_{19}N_6S_2^-$ [M-H]$^-$ 407.1113, found 407.1106. **Elem. Anal.**: Found C; 58.9 %, H; 4.94 %, N; 20.5 %. $C_{20}H_{20}N_6S_2$. Calc.: C; 58.80 %, H, 4.93 %, N; 20.57 %.

3. Fluorescence Spectroscopy

Table S.1. Fluorescence spectroscopy data of bis(thiosemicarbazonato) ligand precursors (DMSO, 100 µM stock solutions).

Compound	$\lambda_{ex\text{-}max}$ / nm	Excitation range / nm	$\lambda_{em\text{-}max}$ / nm	Emission range / nm
**II**	470	260-610	618	432-800
**2**	490	240-615	554	454-735
**3**	490	380-530	550.5	496-663
**4**	480	260-560	547	499.5-695
**Ga(3)**	500	250-270	556	500-685
**Ga(4)**	520	250-550	554	490-690

Ranges of absorption and emission were assessed using 2D contours, which are significant to the choice of cytotoxicity assays and fluorescence imaging settings. Since relevant cytotoxicity assays use absorbance readings at 570 nm, it is preferable that there should be minimal or no excitation at this wavelength. This was confirmed for each ligand precursor making them suitable for later *in vitro* studies. In general the fluorescence of the bis(substituted) ligand precursors was very weak, with the overall range of absorption for these compounds approximately between 260 nm and 550 nm and an emission range of 450 nm and 680 nm.

The wavelengths resulting in maximum excitation ($\lambda_{ex\text{-}max}$) were found between 470 to 490 nm, indicating that compounds would be appropriate for excitation *via* a standard 488 nm confocal microscopy laser. With $\lambda_{em\text{-}max}$ lying between 545 and 555 nm the emission can be expected in a standard green channel of a confocal microscope. The notable exception of methyl substituted ligand **II** with the λ_{max} of emission at 618 nm and a significantly broader emission range of 432 nm to 800 nm is likely to be observable in both green and red channels of a standard confocal microscope. The mono(substituted) ligand precursor was only weakly fluorescent compared to the corresponding bis(substituted) compound. As a representative example the allyl-substituted mono(substituted) ligand precursor showed broader excitation and emission (240 nm to 615 nm and 454 nm to 735 nm respectively). While $\lambda_{ex\text{-}max}$ and $\lambda_{em\text{-}max}$ were 490 nm and 554 nm respectively for **2**, were comparable to the maxima of **4** *(***Table S.1.***)*.

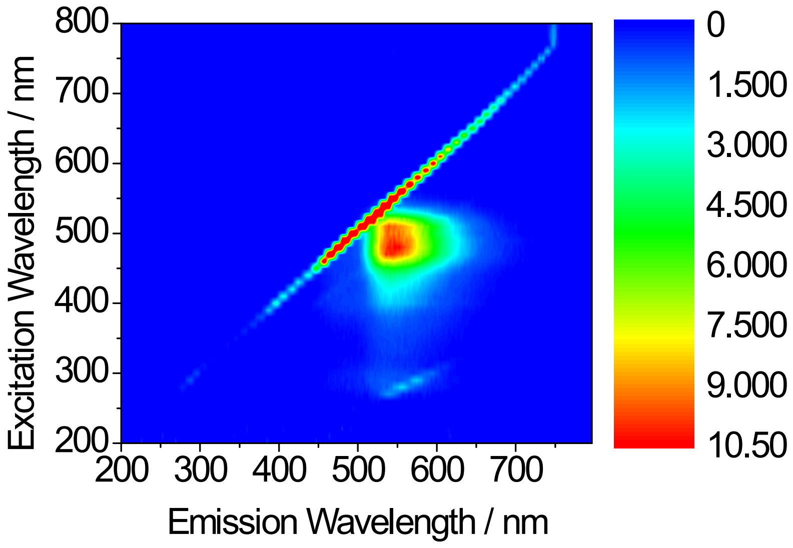

Figure S.1. Excitation/emission map of compound 4.

Compounds generally emitted between 520 and 675 nm, whereas excitation mainly occurred between 250 and 570 nm. All compounds displayed intrinsic fluorescence. $\lambda_{em\text{-}max}$ was found to vary for gallium complexes depending on the functionalities: 510 nm, 400 nm, 450 nm and 520 nm for Ga(3) and Ga(4) complexes respectively.

Quantum yields were calculated using the standard solution of $[Ru(bipy)_3](PF_6)$ in water as a reference and utilising the equation:

$$\Phi_S = \Phi_R \cdot \left(\frac{D_S}{D_R}\right) \cdot \left(\frac{A_R}{A_S}\right) \cdot \left(\frac{I_R}{I_S}\right) \cdot \left(\frac{\eta_S}{\eta_R}\right)^2$$

Where Φ = quantum yield, D = integrated area under emission band, S = sample, A = absorbance of solution at excitation wavelength, R = reference, η = refractive index of solvent, I = maximum intensity of excitation band $[Ru(bipy)_3][PF_6]_2$ in water was used as a reference, with a quantum yield of 0.042. Importantly, the quantum yields indicate sufficient fluorescence imaging in biological cells, with similar values for each gallium compound.

Table S.2. Fluorescence quantum yield measurements of gallium complexes.

Compound	$\lambda_{ex\text{-}max}$ / nm	Excitation range / nm	$\lambda_{em\text{-}max}$ / nm	Emission range / nm	Quantum Yield
**Ga(II)**	410	250-560	551	495-685	0.11
**Ga(3)**	500	250-570	556	500-685	0.19
**Ga(4)**	520	250-550	554	490-690	0.12

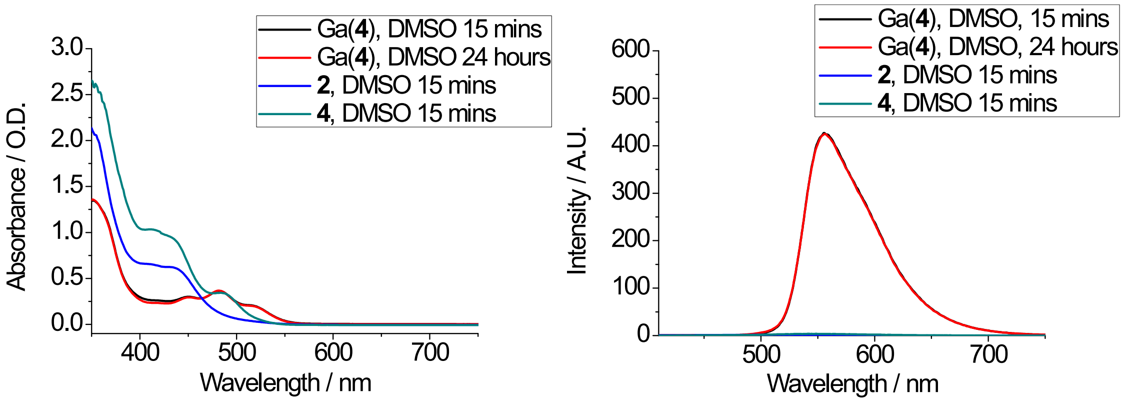

a) **b)**

Figure S.2. Spectroscopy of compound Ga(**4**) at a total concentration of 100 µM, a) single photon (steady state) and b) UV-Vis, monitored at time intervals of up to 24 h at room temperature.

5. Radiochemistry

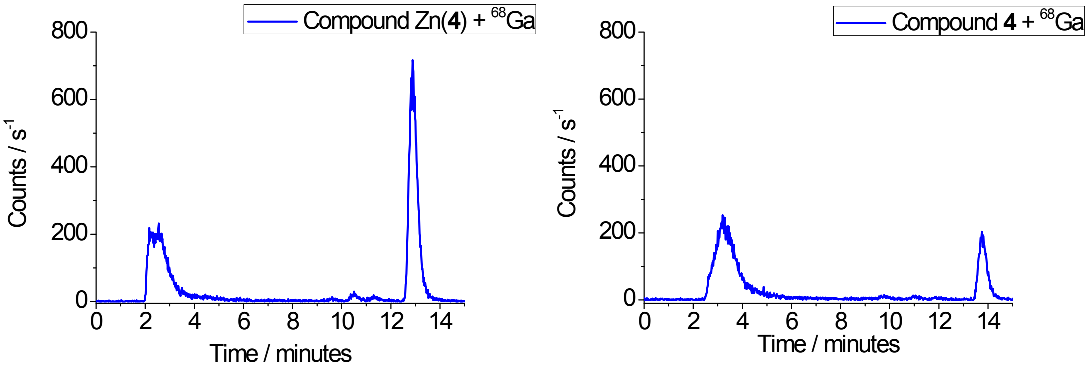

a) **b)**

Figure S.3. Standard (unoptimised) radio-HPLC trace (blue) of (a) aromatic zinc bis(thiosemicarbazonato) precursor compound Zn(4) (R = allyl) and (b) ligand precursor 4 radiolabelled with $^{68}GaCl_3$ using conventional heating techniques.

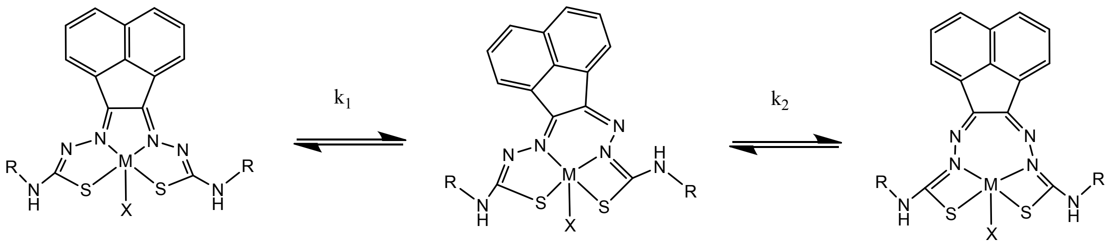

Sym [Xray/NMR] asym [Xray/NMR] [not observed]

For M = Zn: X = DMSO
For M = Ga(III): X = Cl⁻
R = Et

Figure S.4. Possible isomeratisation equilibria for the coordination of bis(thiosemicarbazonato) ligands to the metal centre.

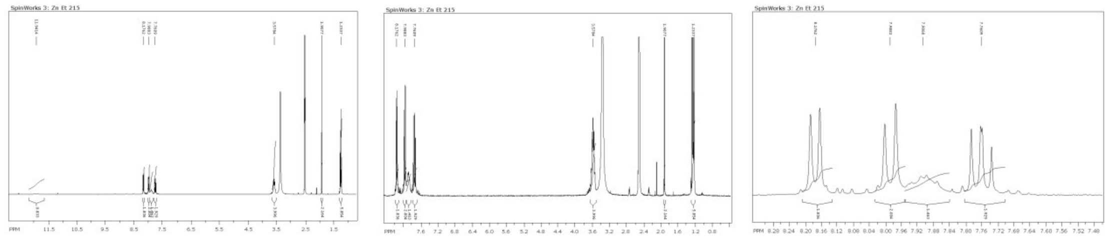

Figure S.5. Isomeric change observed with time in ^{1}H-NMR (DMSO d^6). This may account for some broadening in the HPLC data (The usa of TFA in the solvent system seems to solve this problem).

Gallium-68 labelling (uncomplexed $GaCl_3$: 20 min needed to evaporate 100-200 µL aq. HCl at 90 ºC under Ar depending on Ar flow).

Total: 300 µL scale (50 µL DMSO stock (1 mg/mL) + 250 µL EtOH; starting with ca. 18.5 MBq in 100 µL HCl).

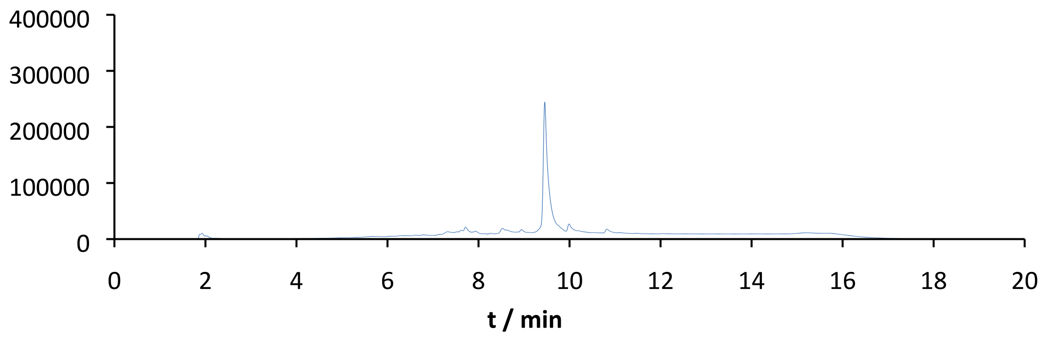

Figure S.6. HPLC trace of 24 h @ r .t. old stock solutions in DMSO [equilibrated]

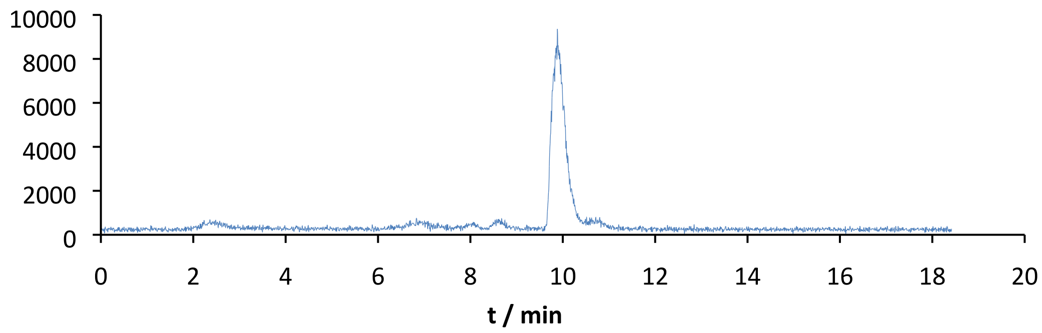

Figure S.7. RadioHPLC trace ethyl-derivatised Zn(II) complex and aq. Ga-68 after 10 min in the microwave at 90 ºC.

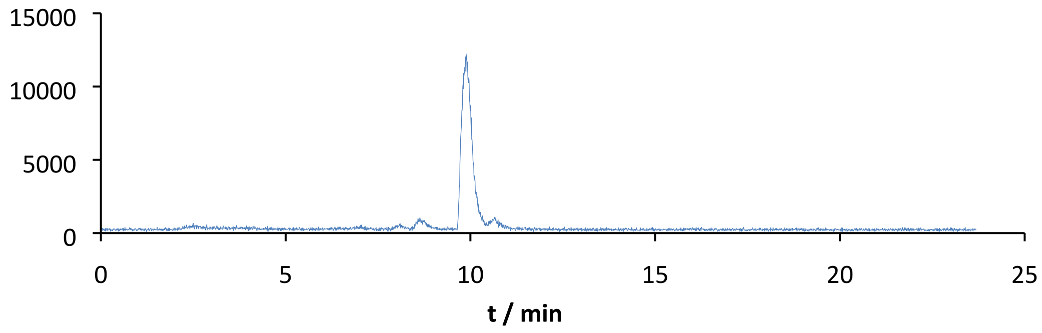

Figure S.8. RadioHPLC trace Zn(II) complex and aq. Ga-68; after 45 min heat 90 ºC.

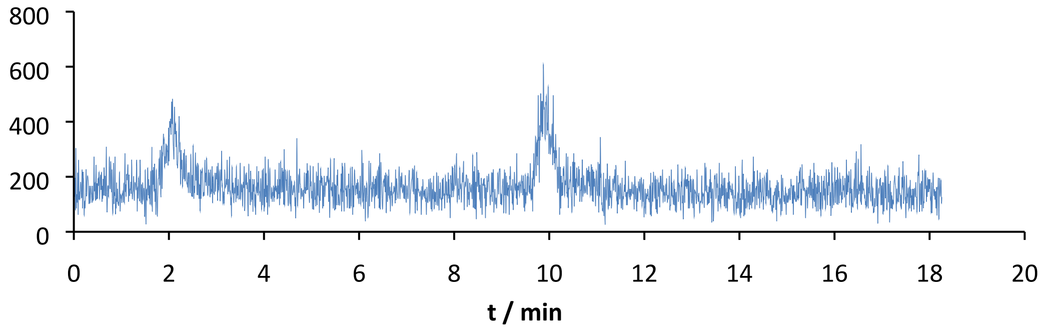

Figure S.9. Radio HPLC trace after saline wash 200 µL, 30 min -1h at r.t.

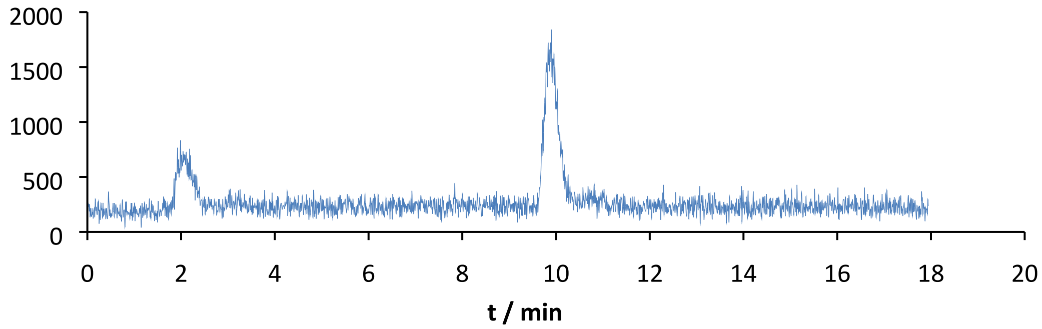

Figure S.10. Radio HPLC trace after the addition of EDTA, 200 µL, 30 min-1 h r.t.

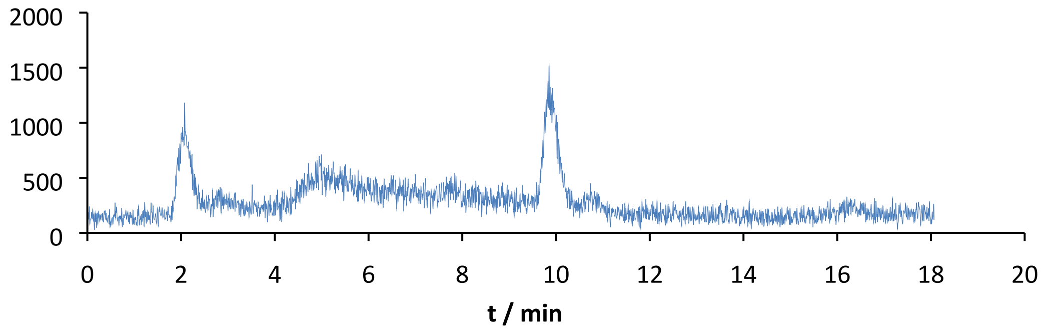

Figure S.11. Radio HPLC trace after the addition of citric acid 200 µL 30 min- 1h r.t.

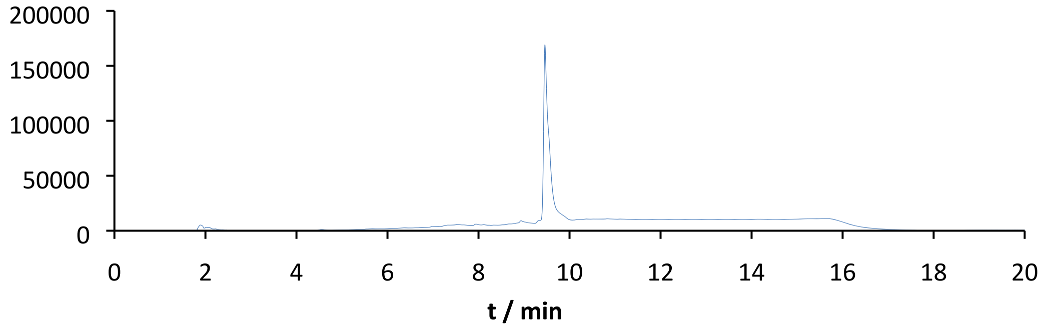

Figure S.12. Radio HPLC trace after 10 min microwave, 90 ºC, large volume in test tube. Scale up semic-prepHPLC conditions: $CH_3CN:H_2O$ 40:60 reverse phase C18 HPLC (0.1%

TFA / 0.1% TFA) using 100 µL DMSO stock of Zn complex + 700 µL EtOH (ca. 55.5 MBq / 200 µL HCl scale) and starting with 111 MBq. Only fresh stock solutions [< 1 h] were used and no major changes with 24 h in DMSO were observed for R = Et.

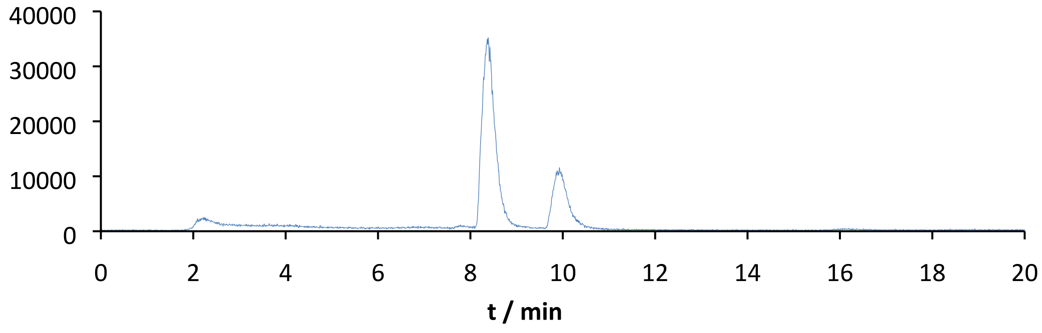

Figure S.13. HPLC trace of asymmetric + symmetric isomers of allyl Ga complex both form at high dilution - Formation of asymmeric isomer favoured. Reaction repeated twice under identical conditions.

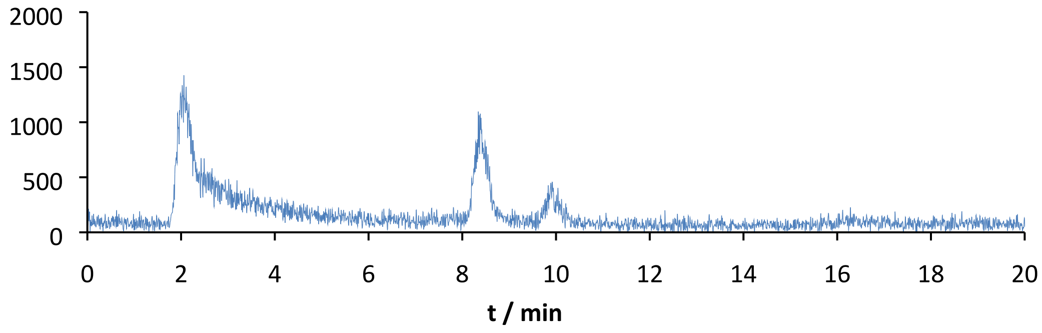

Figure S.14. Radio HPLC trace of PBS wash (200 µl, 30 min at r.t.).

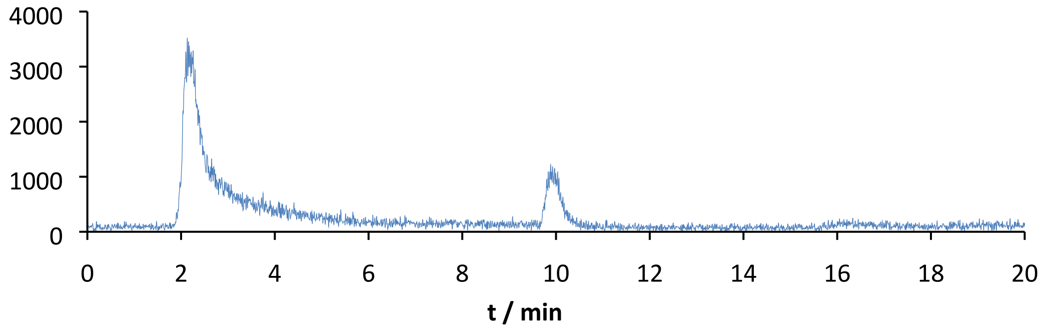

Figure S.15. Radio HPLC trace after the addition of excess of PBS, 500 µL (1h at r.t. Only sym. isomer (most stable) remains and the free GaX_3 conc. increases)

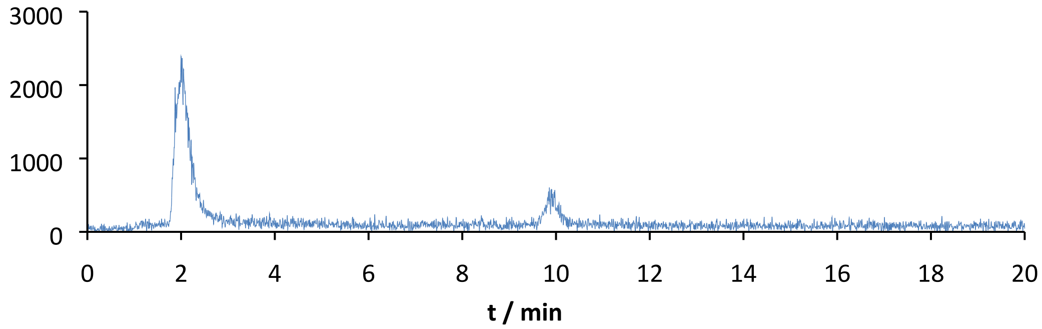

Figure S.16. Radio HPLC trace of EDTA challenge 200 µL 30 min-1h: Only sym. isomer.

Radiochemistry protocol for In vivo

Start with 4 x 111 MBq; 50 µL stock, 250 µL EtOH, 10 min µW heating at 90 ºC, semiprep HPLC purification and concentrated under vacuum. Na_2CO_3 1 M was then added until pH 6-7.

Prep. HPLC: 1 run on a ca. 37 MBq sample. Repeated 3 times and samples merged prior to injection in 3 mice.

Reinjected peak at 10 min after injection for microPET, checked pH ca 6.5 and standing 3 h r.t. (used for in vivo protocol: total 3 h from radiolabelling: increased presence of asymm vs sym isomer). RadioHPLC of sample brought to ready for injection show major isomer and minor isomer in equilibrium so the equilibrium is possibly pH controlled:

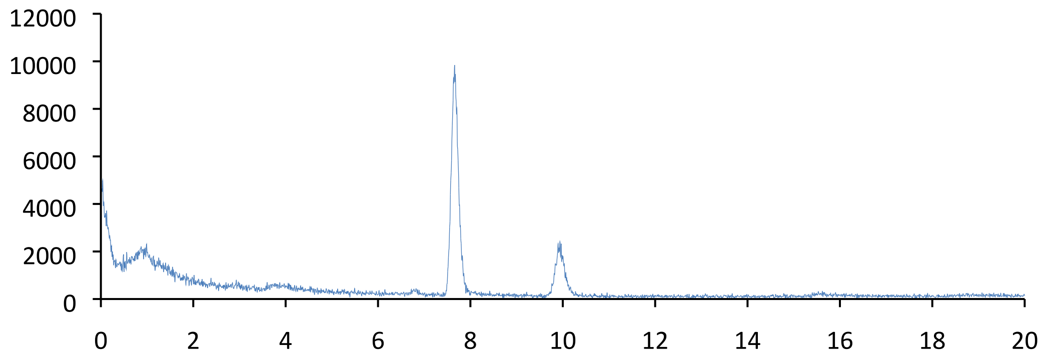

Figure S.17. HPLC trace after injection for microPET.

6. Laser scanning confocal microscopy

Experiments were carried out to ascertain if the weak fluorescence of the ligand precursors was sufficient to be observed *in vitro*. Cells were cultured using standard protocols as described in 1.6.

Cell viability prior to experiments and over their course was monitored by optical microscopy, and validated at the start of the experiments by standard trypan blue tests. The compounds were imaged in HeLa (cervical carcinoma), PC-3 (prostate carcinoma), MCF-7 (breast cancer) and FEK-4 (non-cancerous fibroblast) cell lines using standard confocal fluorescence microscopy with one photon excitation at 488 nm.

The imaging studies were performed using concentrations of 100 μM in a DMSO: RPMI (Royal Park Memorial Institute) cell medium 1:99 solvent mix, whereby the final DMSO concentration on the imaging plate was lower than 1%. The fluorescence of the compounds was very weak, therefore such high concentrations of the ligand precursors were necessary to assess uptake within cells. Compound **I,** the methyl substituted mono(substituted) thiosemicarbazone ligand was incubated in HeLa cells and was found to possess a good colocalisation with lysotracker, suggesting that this compound is likely to enter the lysosome. Further investigations with colocalisation dyes demonstrated that the ligand precursor did not localise in the mitochondria or nucleus. Experiments showed that bis(substituted) ligand precursor **4** possessed weak uptake in HeLa cells, which was barely detectable when incubated in FEK-4 cells, under the same conditions. The latter result suggests that there could be a preference for the compound to enter cancerous cell lines over non-cancerous cells. Optimal imaging conditions were found to be at 405 nm rather than at 488 nm excitation and this emission was observed maximally in the green channel for methyl, ethyl and allyl substituents.

Rather than being a hindrance, the weak fluorescence of the ligand precursors within cells is a significant advantage in being able to assess the stability of their respective metal complexes *in vitro* as will be demonstrated in subsequent chapters. Moreover, stability in cells was subsequently investigated using fluorescence lifetime imaging microscopy (FLIM), as a quantitative means of stability assessment.

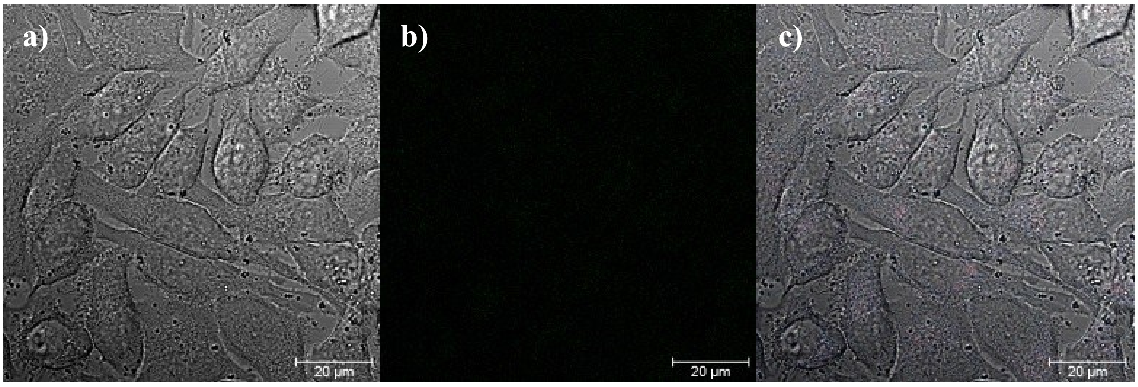

Figure S.18. Confocal imaging of PC-3 cells in serum free medium (EMEM) used as a control. (a) DIC images, (b) fluorescence channel at 488 nm excitation and (c) overlay of the DIC and fluorescence channels. Scalebar: 20 µm.

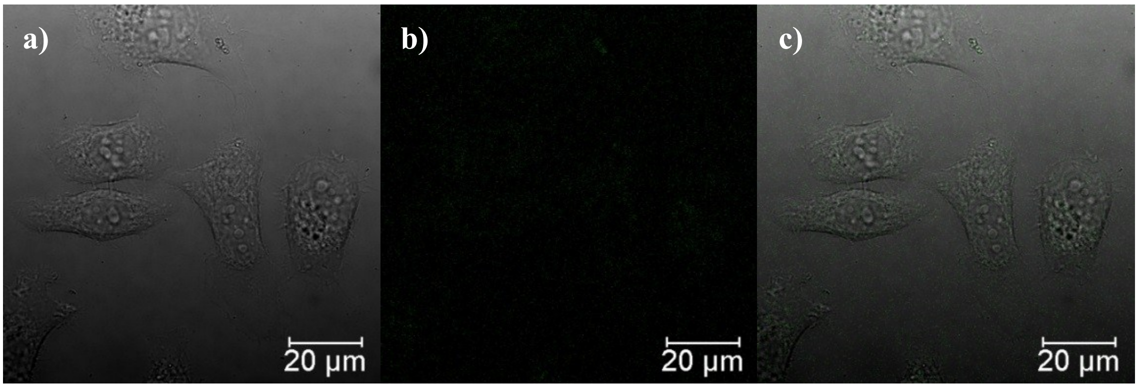

Figure S.19. Confocal imaging of compound **3** at 100 µM, 1% DMSO, 20 minutes incubation, 37°C. (a) DIC images, (b) fluorescence channel at 488 nm excitation and (c) overlay of the DIC and fluorescence channels. Scalebar: 20 µm.

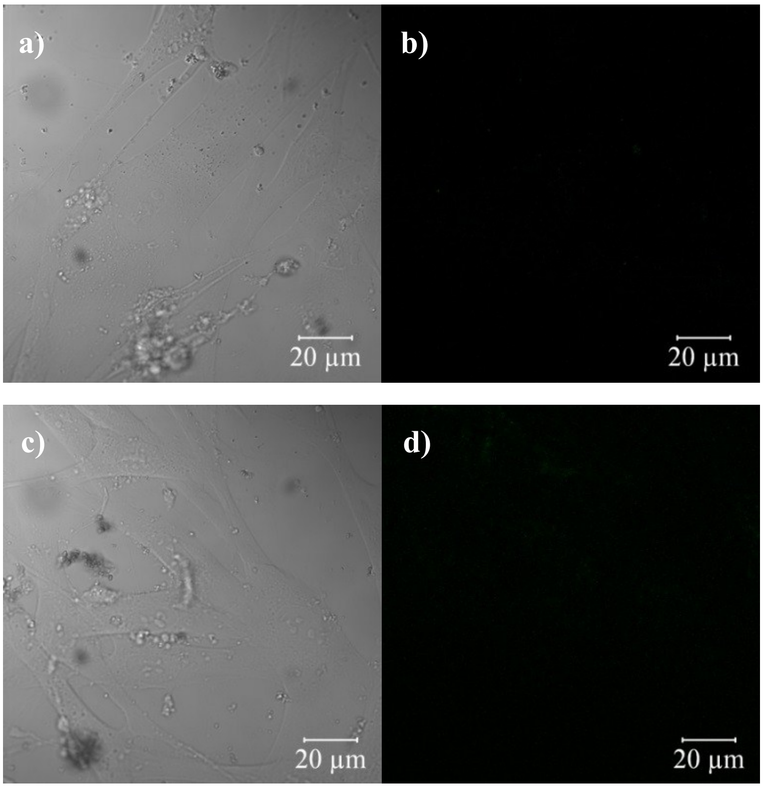

Figure S.20. Confocal microscopy of compound **4** at 100 μM, 1 % DMSO, 20 minutes incubation time in, (a, b) FEK-4 living cells and (c, d) HeLa living cells. (a, c) DIC images and (b, d) micrographs of the fluorescence channel with excitation at 488 nm. Scalebar 20 μm

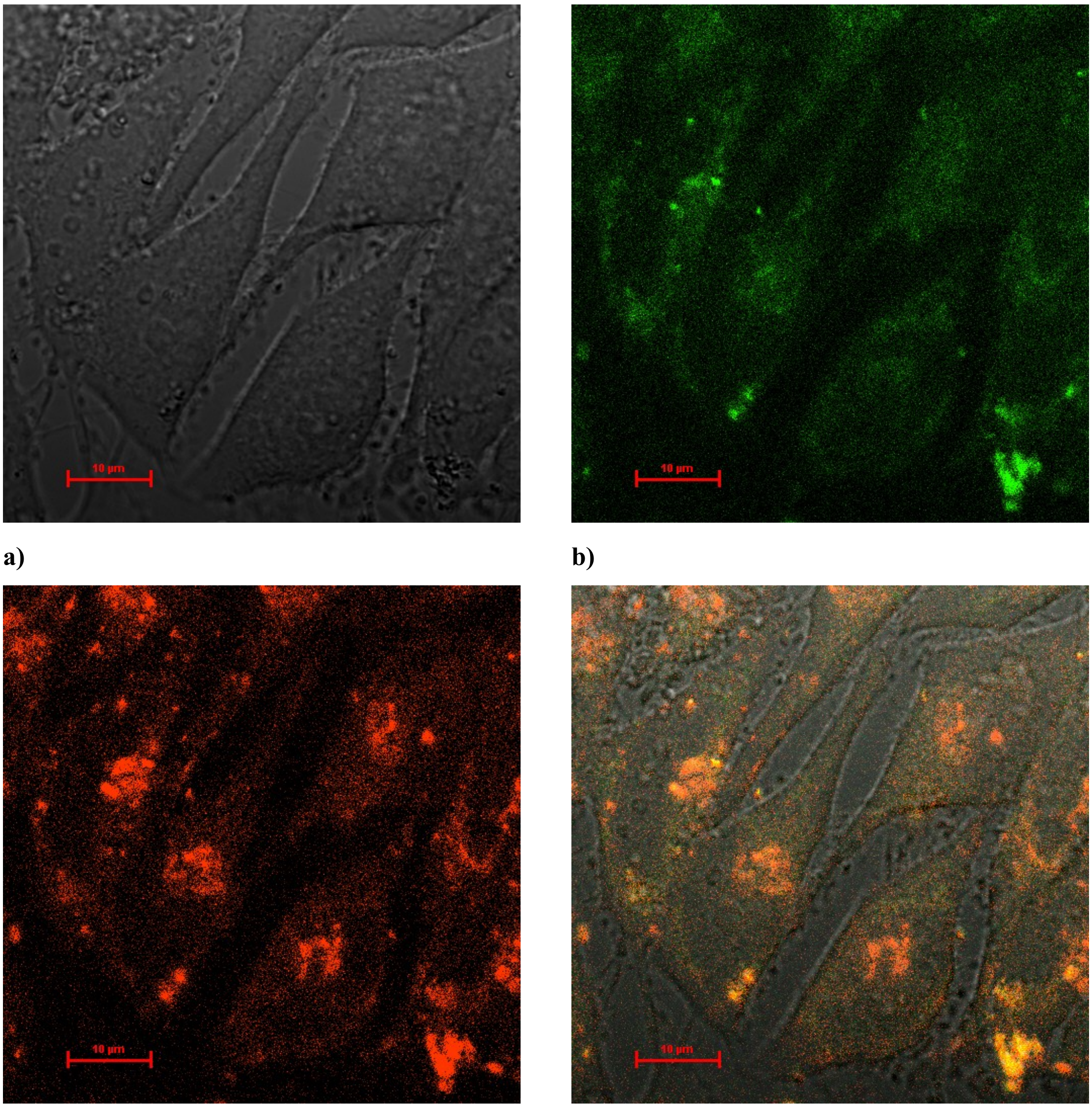

a) b) c) d)

Figure S.21. Single-photon confocal microscopy images of **I** in HeLa cells, incubated with lysotracker 100 µM, 1% DMSO, 20 minutes, at 37°C. a) Differential Interference Contrast (DIC) image. b) Micrograph of cells after excitation at 488 nm, green channel, showing localisation of the compound I. c) Micrograph of cells after excitation at 543 nm, red channel, showing localisation of the lysotracker. d) Overlay of a), b) and c). Scalebar: 10 µm.

.

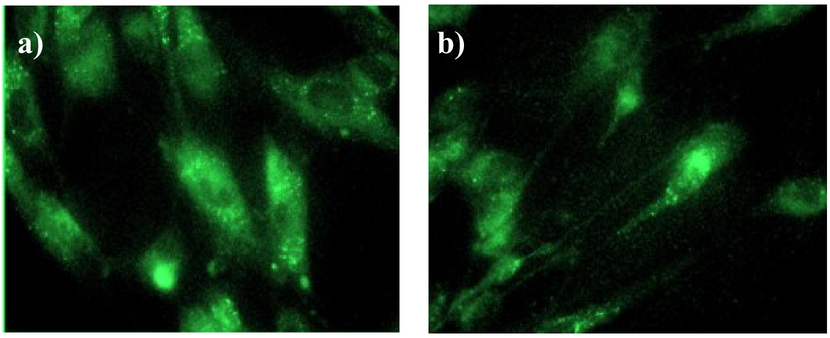

Figure S.22. Confocal micrographs of compound Ga(**4**) at 100 µM, 1 % DMSO, 20 minutes incubation time in FEK-4 living cells under, (a) normoxic and (b) hypoxic conditions in the fluorescence channel with excitation at 488 nm. Scalebar 20 µm.

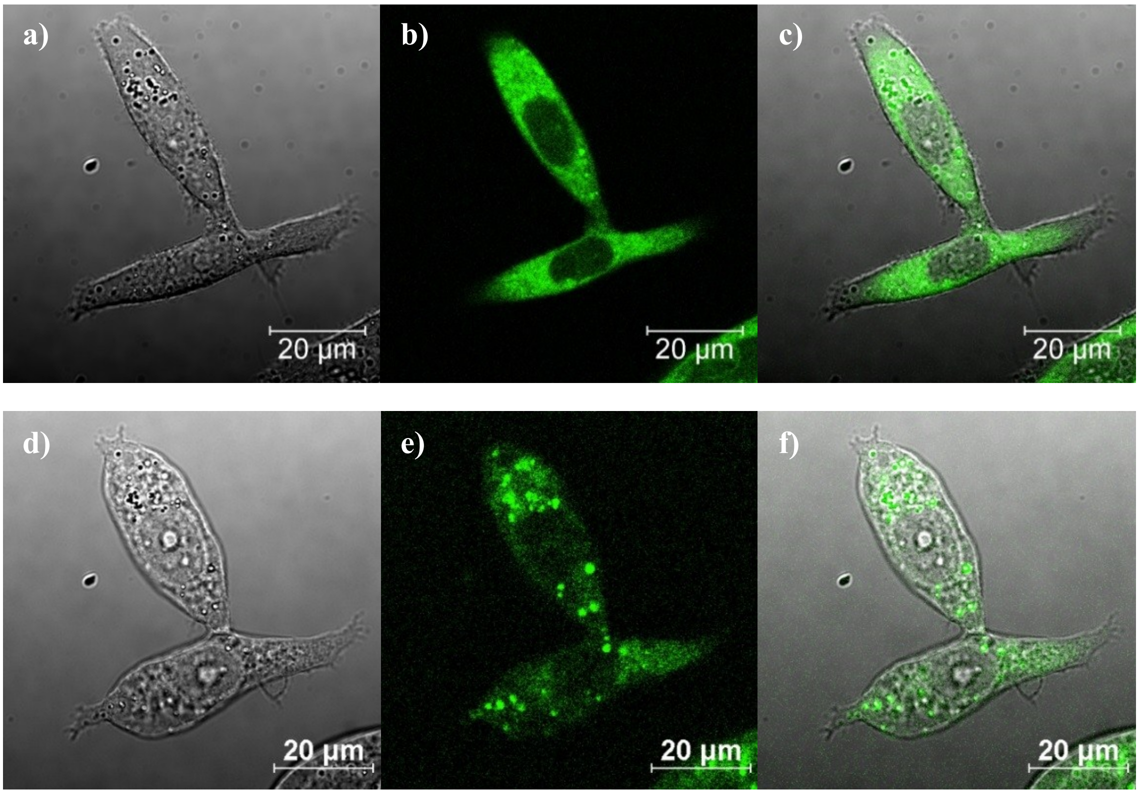

Figure S.23. Confocal imaging of complex Ga(**3**) in PC-3 cells at 50 µM, 0.5% DMSO, 20 minute incubation and 37°C during an irradiation experiment at 488 nm. Micrographs showing images recorded, (a – c) before irradiation and (d – f) after irradiation for ca. 10 minutes. (a, d) DIC images, (b, e) fluorescence channel at 488 nm excitation and (c, f) overlay of the DIC and fluorescence channels. Scalebar: 20 µm.

Complexes Ga(**3**) and Ga(**4**) was imaged in HeLa (cervical carcinoma), PC-3 (prostate carcinoma), MCF-7 (breast cancer) and FEK-4 (non-cancerous fibroblast) by confocal

fluorescence microscopy using one photon excitation at 488 nm with the emission long pass filtered at 515 nm.

The imaging studies were performed using concentrations of 50 μM compound in a DMSO: RPMI 0.5:99.5 cell medium solvent mix, whereby the final DMSO concentration on the imaging plate was lower than 1%. The solutions were left to incubate with the adherent cancer cell lines for 20 or 60 min, with preliminary investigations incubating up to 3 hours and at concentrations of 100 μM, at 4 °C or 37 °C. The cells were carefully washed with Phosphate Buffered Saline (PBS) pre-warmed to 37°C, which was replaced by FCS-free medium to remove the non-internalised fluorescent dispersion prior to fluorescence imaging.

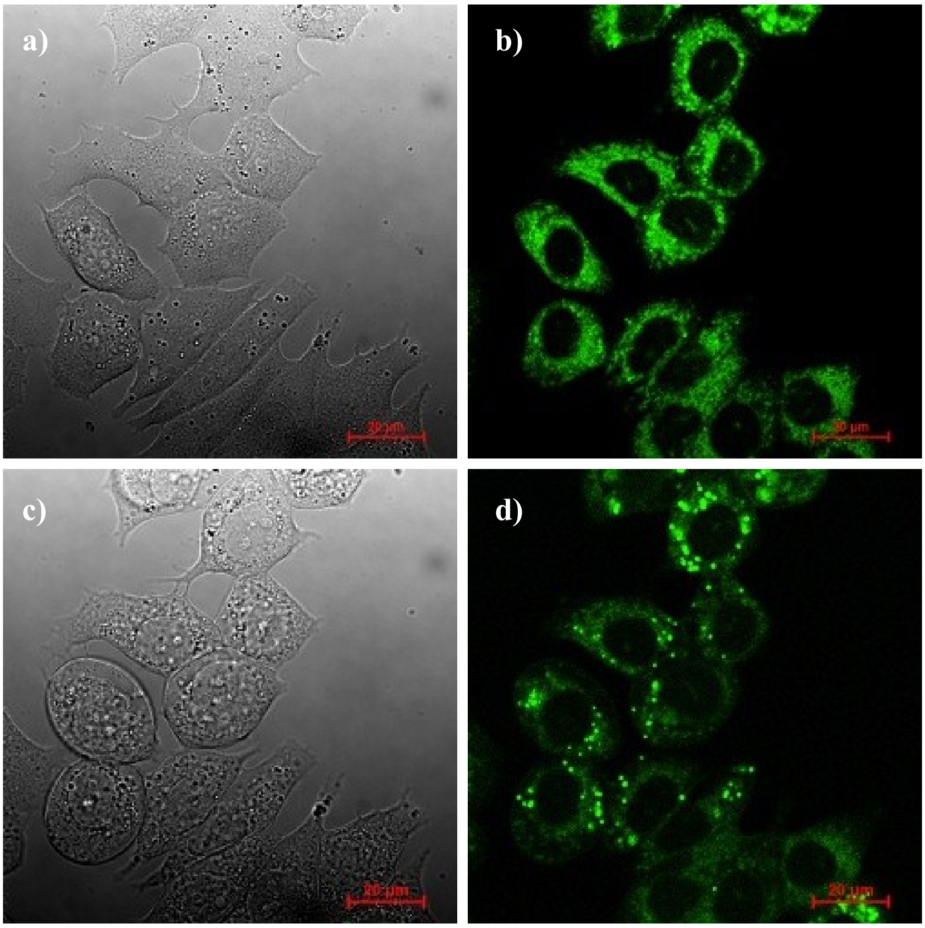

Figure S.24. Complex Ga(**3**), 50 μM, 0.5% DMSO, 20 minutes, at 37°C irradiation experiment at 488 nm in MCF-7 cells: (a, b) before irradiation and (c, d) after irradiation for ca. 10 minutes, where DIC image (a, c), micrograph of cells after excitation at 488 nm (b, d) Scalebar: 20 μm.

Co-localisation dyes and cells are shown and were recorded a minimum incubation of 20 minutes and a maximum of up to 3 hours. The observation of fluorescence in these images suggests that substantial amounts of these complexes remain intact inside the cell. Within the first 20 minutes there is widespread distribution of the gallium complexes within the cell cytoplasm.

Co-localisation with commercially available dyes (e.g. Mitotracker Red and Green, Hoechst, Lysotracker Red and Green) were used to identify regional uptake within the cell, investigating whether the gallium complexes enter the mitochondria, lysosomes, the nucleus and lipid-rich regions of the cell, which may in turn shed light upon the nature of compound activity.

Additionally, complex **Ga(3)** displayed interesting photoinduced changes in intracellular localisation within cells and was studied in MCF-7 and PC-3 cells, showing comparable data in each cell line. After irradiation with 488 nm light the green fluorescence was less evenly distributed within the cell with a more punctuated appearance. It was noticeable that the cell morphology was dramatically altered resulting in rounder and larger cells indicating occurrence of blebbing, which occurs during the process of apoptosis (programmed cell death).

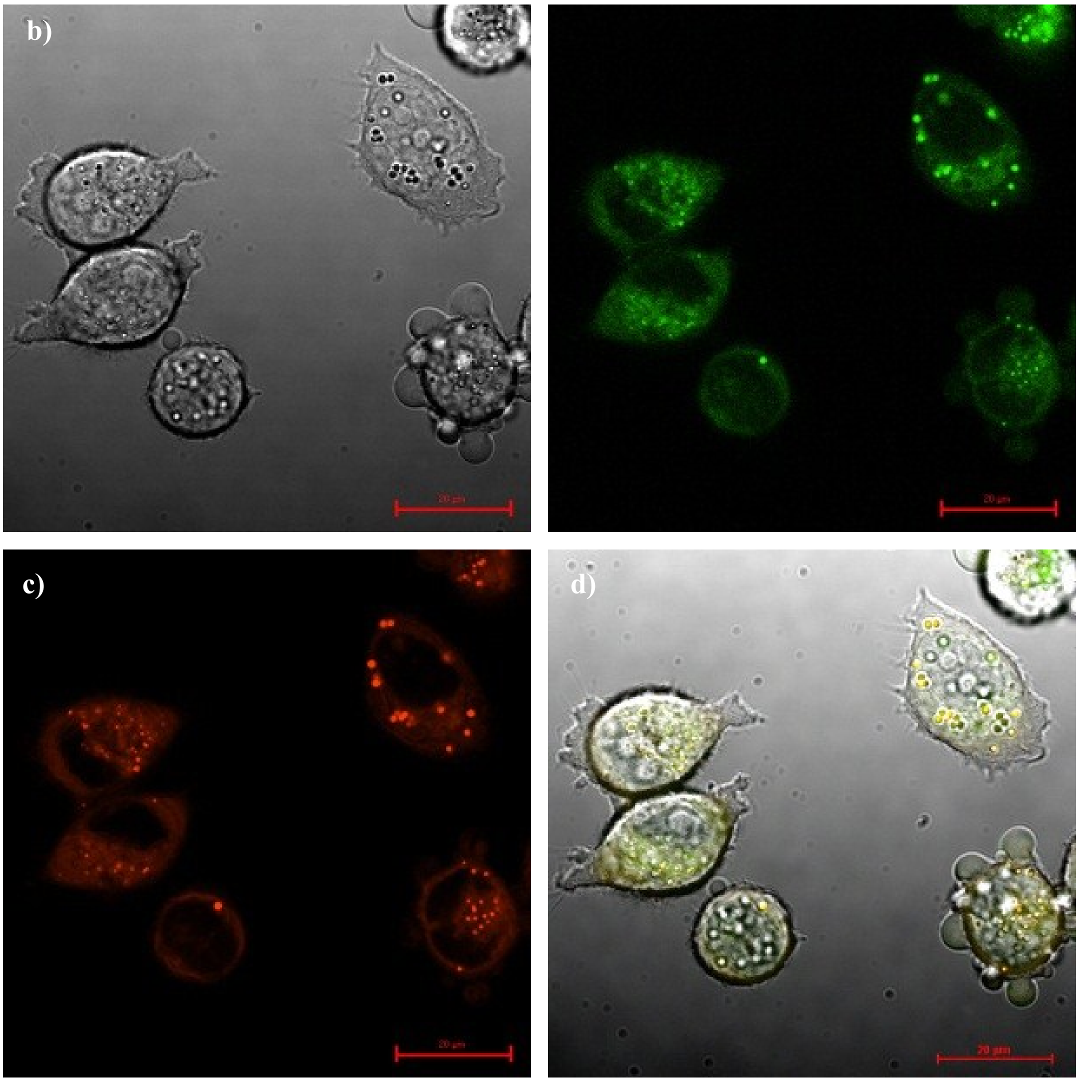

Figure S.25. Complex Ga(**3**), 50 µM, 0.5% DMSO, 20 minutes at 37°C and Nile Red in MCF-7 cells after ca. 5 minutes of irradiation: DIC image (a), micrograph of cells after excitation at 488 nm (b, compound, green channel), micrograph of cells after excitation at 543 nm (c, Nile Red, red channel). Image (d) is an overlay of (a), (b) and (c) images suggesting localisation with lipid rich regions of the cell. Scalebar: 20 µm

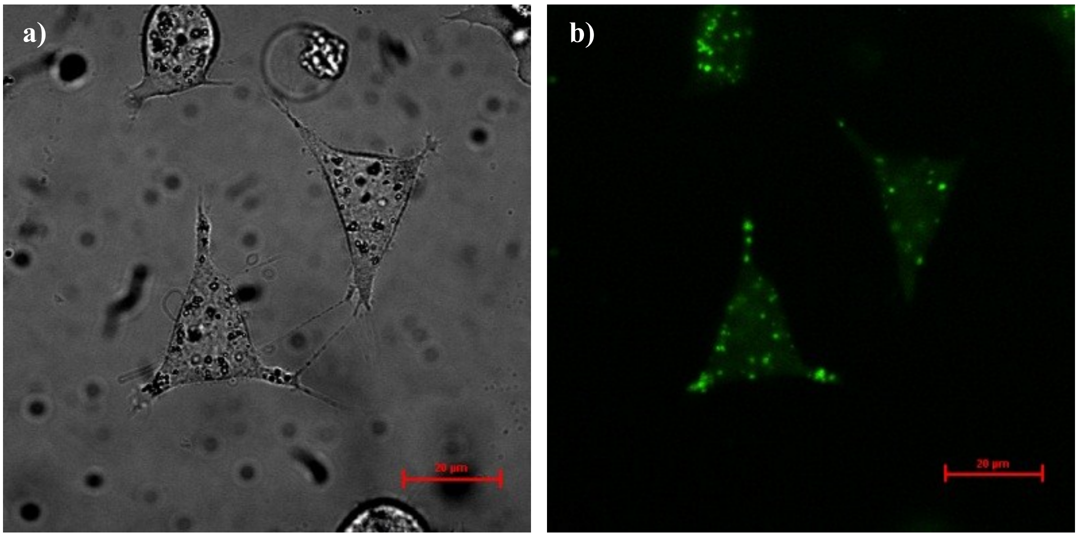

Figure S.26. Single-photon confocal fluorescence imaging: Complex **Ga(4)**, 50 µM, 0.5% DMSO, at 37°C in HeLa cells incubated for 1 hour: (a – b) the cells were irradiated with 488 nm light for less than 5 minutes, where DIC image is represented by (a) and micrograph of cells after excitation at 488 nm by (b, compound, green channel). Scalebar: 20 µm.

Interestingly, complex **Ga(4)** when incubated for 60 minutes, punctuation was also observed during the process of finding a representative area to image. A further study was carried out incubating HeLa cells with **Ga(4)** for 3 hours at 100 µM, 1% DMSO to determine if this effect occurs without irradiation when incubation is longer and more compound is present. This effect was not observable in absence of irradiation indicating that this process does not occur with time alone. Additionally, the study was carried out at 37 °C and 4 °C, to investigate if the uptake occurred by passive diffusion alone, since mechanisms such as endocytosis do not occur at low temperatures. **Ga(4)** entered cells both at 4 ºC and 37 ºC, implying that cell uptake occurs by passive diffusion alone in fixed cells with the nucleus stained with DAPI. Nuclear uptake was observed at 37 ºC, but not at 4 ºC, signifying that the process did not occur by passive diffusion.

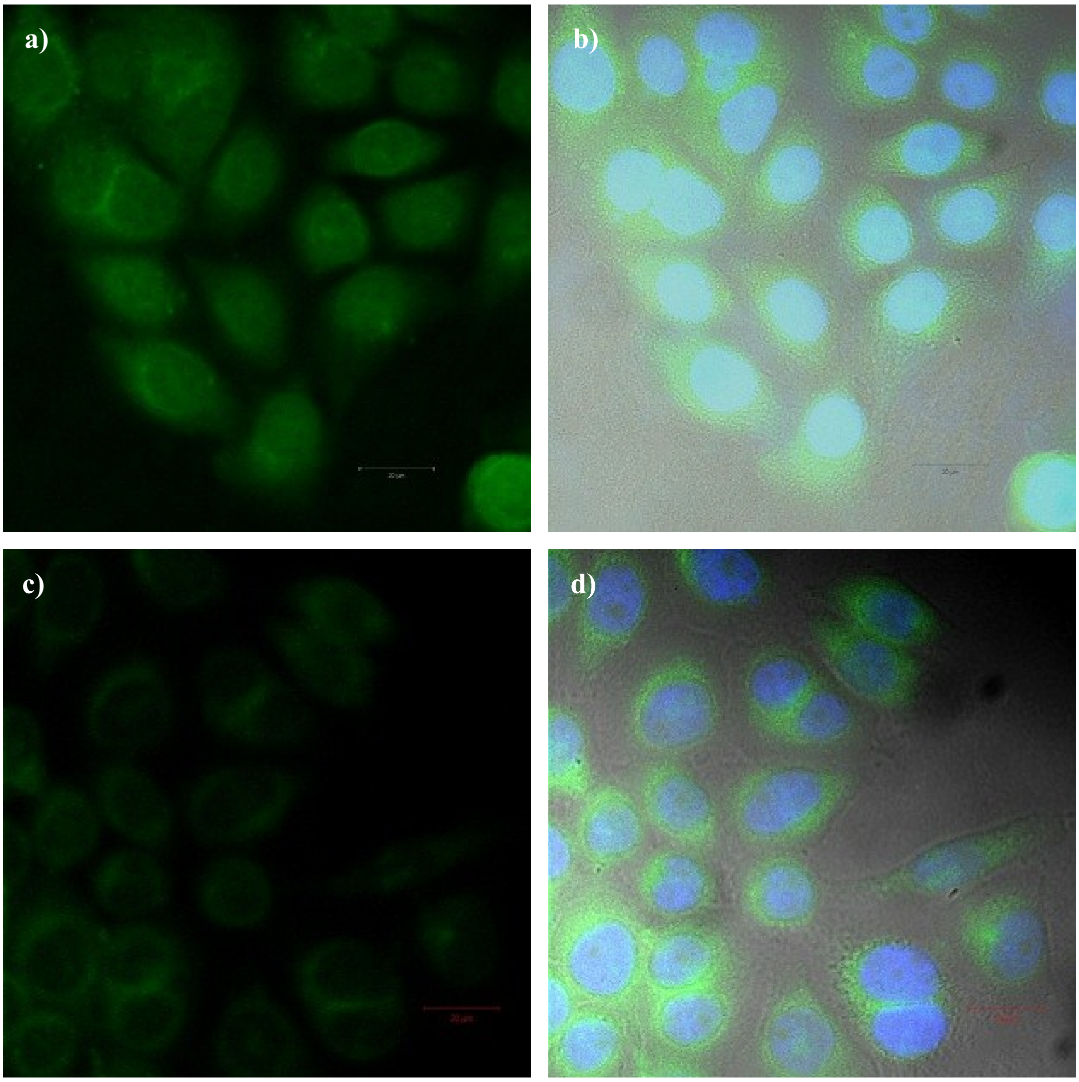

Figure S.27. Single-photon confocal microscopy images of complex Ga(4) and DAPI in HeLa (fixed cells), incubated for 3 h, 100 μM, 1% DMSO where (a) – (b) at 37 ^{o}C and (c) – (d) at 4 ^{o}C; (a) and (c) represent the micrograph with excitation at 488 nm, emission >505 nm, (b) and (f) represent an overlay of the micrograph of excitation at 405 nm, emission 420-480 nm (a) and (c) respectively and the DIC image.

The study was further carried out with **Ga(3)** with more standard incubation times of 20 minutes and 50 μM 0.5% DMSO, to determine if the same effect is observed for shorter incubations and lower concentrations, which indicated no nuclear uptake at 4 ºC and very little complex within the nucleus at 37°C signifying that entry to the nucleus is only significantly achieved at longer time-points as above. Interestingly it appears that the fluorescence of **Ga(3)** was red-shifted at 4 ºC as well as being taken up by HeLa cells .

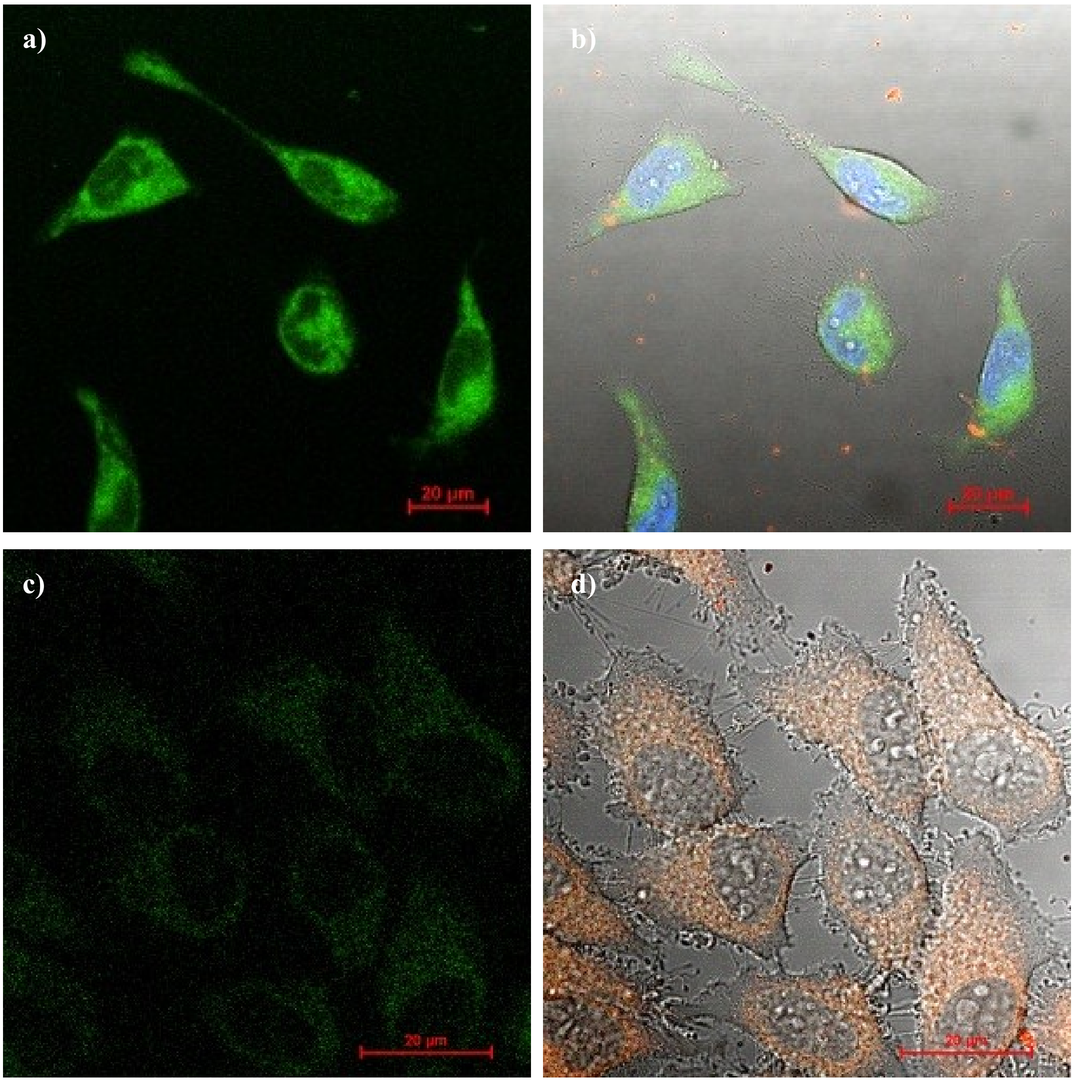

Figure S.28. Single-photon confocal microscopy images of (a-d) Complex Ga(**3**), 50 μM, 0.5% DMSO, 20 minutes and Hoechst (nuclear stain) in HeLa cells at 37°C: DIC image (a), micrograph of cells after excitation at 488 nm (b, compound, green channel), micrograph of cells after excitation at 543 nm (c, compound, red channel). Image (d) is an overlay of (a), (b), (c) and the micrograph from excitation at 405 nm. (e-h) Ga(**3**), 50 μM, 0.5% DMSO, 20 minutes in HeLa cells incubated at 4 °C, (e) DIC image, (f) excitation at 488 nm, emission >505 nm, (g) after excitation at 543 nm and (h) is an overlay of (e), (f) and (g). Scalebar: 20 μm.

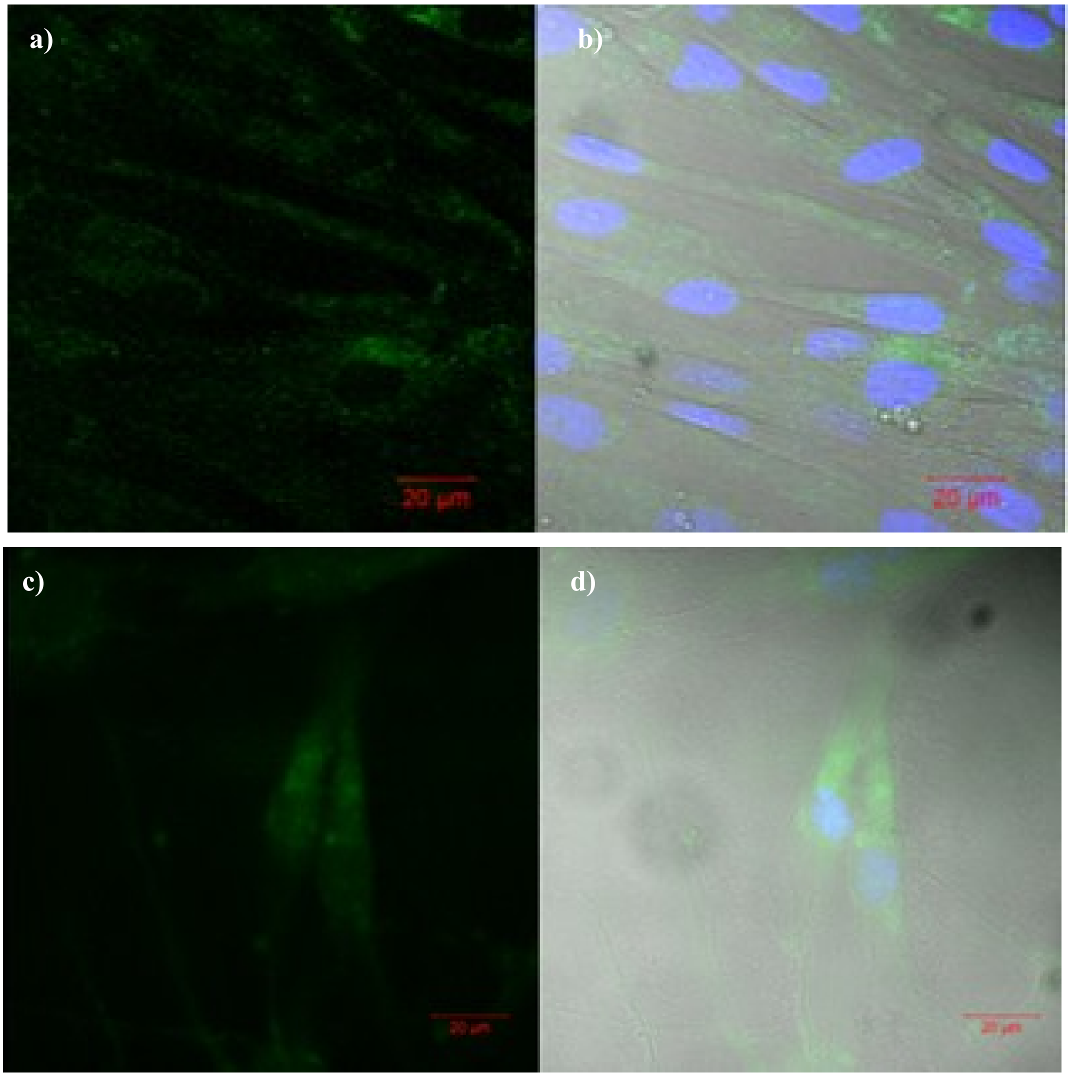

Figure S.29. (a-b) Complex Ga(4) in FEK-4 cells, 50 µM, 0.5% DMSO, 20 minutes: (a) is micrograph of cells after excitation at 488 nm (compound, green channel). Image (b) is an overlay of (a), the DIC image and micrograph of cells after excitation at 405 nm. Figure (c-d) Complex Ga(4) irradiation experiment at 488 nm in FEK-4 cells, 100 µM, 1 % DMSO, ca. 3 hours images, fixed cells stained with DAPI, scalebar: 20 µm, (c) is micrograph of cells after excitation at 488 nm, (d) is an overlay of (c), the DIC image and micrograph of cells after excitation at 405 nm.

These gallium complexes enter both cancer and non-cancer cells. Localisation appears to occur in many organelles and indicates that, if cytotoxic these compounds may have many modes of action. Long incubations saw the complex Ga(**4**) enter the nucleus indicating a possibility DNA damage during prolonged exposure, whereas rapid uptake was observable in

the lysosome and the mitochondria, meaning that these complexes may possess potential to disrupt these organelles. Data suggest that the complex can enter cells by passive diffusion, yet enters the nucleus *via* other means.

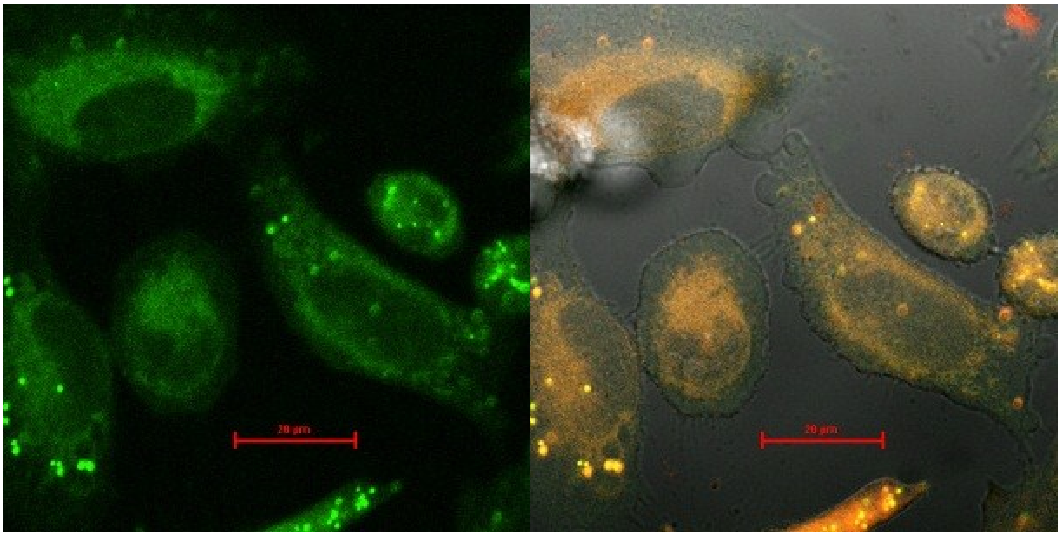

c) d)

Figure S.30. Complex **Ga(4)**, 50 µM, 0.5% DMSO, 20 minutes at 37 °C and Lysotracker in HeLa cells, (a) micrograph of cells after excitation at 488 nm, (c) is an overlay of DIC image and micrograph of cells after excitation at 543 nm. Scalebar: 20 µm.

7. Two-photon excitation and fluorescence lifetime imaging microscopy

Two-photon absorption occurs when the combined energy of two simultaneous photons results in molecular excitation. The first photon to interact generates what is known as a virtual state, which has no classical analogue and only exists transiently (generally for femtoseconds) in which time a photon can travel ca. 1 μm. Arrival of the second photon must occur before the virtual state decays (dephases), which will occur if the laser intensity is high and focussed. The probability of single photon absorption is proportional to the intensity of light and is in contrast to two-photon absorption, which is dependent on both spatial and temporal coincidence *(Equations 2.1 and 2.2)*.[7] Therefore, where NA_x is the number of photons absorbed per second, I is the intensity, σ_x is the cross-section and x is 1 or 2 photon absorption.

Single-photon absorption: $NA_1 = \sigma_1 I$ (Equation 2.1)

Two-photon absorption: $NA_2 = \sigma_2 I^2$ (Equation 2.2)

An advantage of two-photon excitation therefore, is that imaging occurs only from the focal plane. This is contrasting with single-photon excitation microscopy, in which the image is often distorted as it may occur outside the focal plane.[7] Furthermore, despite the requirement of high-powered lasers, two-photon microscopy has been found to cause less damage to biological cells than single-photon microscopy, as well as to decrease photo-bleaching and augment imaging depth.[8]

Fluorescence lifetime is often considered as a means of distinguishing between different fluorophores, which possess sufficiently different lifetimes and can also be achieved using spectrally resolved microscopy.

FLIM is frequently used to separate different portions of the same fluorophore, for purposes such as quenching of luminescence by intracellular ions (e.g. Ca^{2+} or Cl^-) or by oxygen. Furthermore, this technique can be used to monitor binding of and even distance from a fluorophore, with a conformational change is likely to result in a different rate of internal non-radiative decay and, therefore, a modification of the fluorescence lifetime.[9, 10] pH effects can also be studied, since protonated and non-protonated forms of a molecule may possess different lifetimes. In addition investigations of aggregation, viscosity, proximity to metal surfaces and nanoparticles (due to their long luminescent lifetimes) are carried out.[9] There

has, however, been little use of FLIM for the purpose of assessing the stability of sodium[11] and magnesium[12] complexes *in vitro*, but thus far no reports of use of the stability evaluations technique with transition metal complexes.

Consequently, if the fluorescence lifetime of the complex is significantly different to that of the ligand precursor, FLIM could be utilised to assess the complex stability *in vitro*. Two-photon excitation experiments were performed at the Rutherford Appleton Laboratory following the methodology described in Botchway *et al.* 2008[13] and Pascu *et al.* 2011.[14]

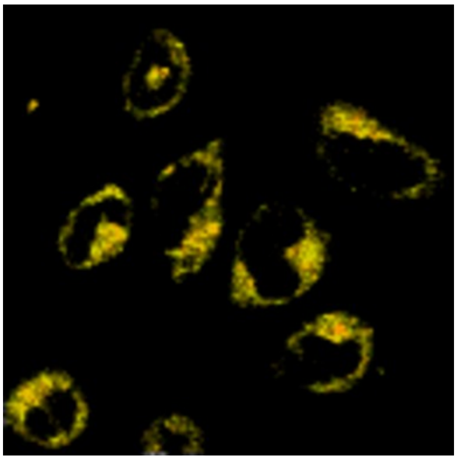

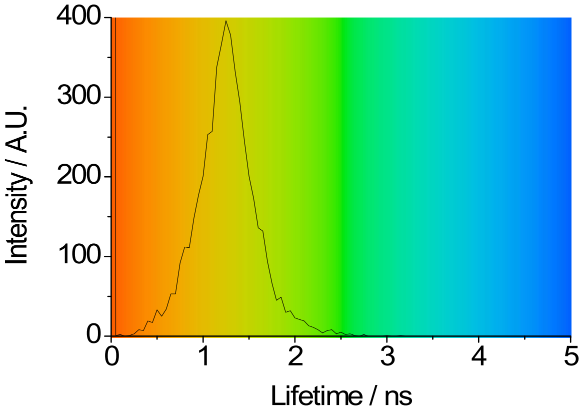

a) **b)**

Figure S.31. Control experiment at 810 nm, in un-treated HeLa cells where **a)** is the colour image corresponding to the two-photon fluorescence lifetime map of the τ_m (the weighted average fluorescence lifetime), **b)** is the distribution curve of the τ_m where the colour represents the lifetime of a).

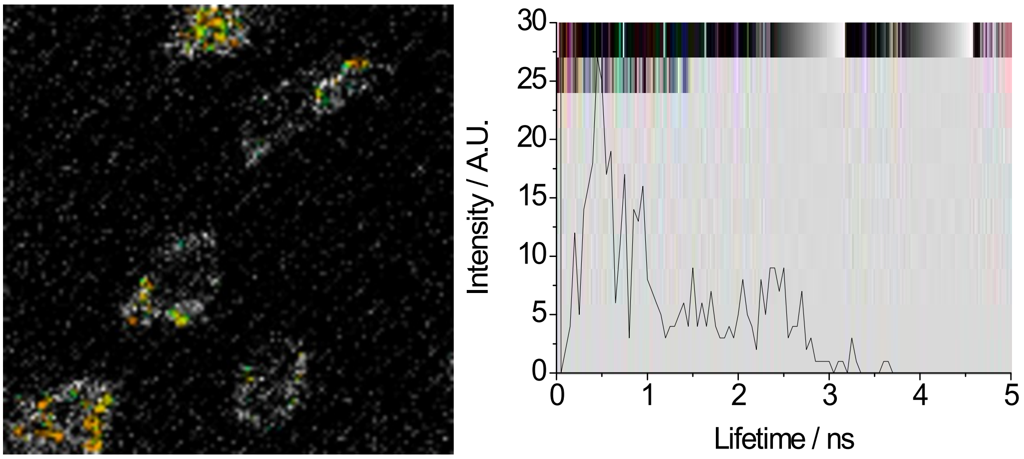

a) **b)**

Figure S.32. Control experiment at 910 nm, two-photon fluorescence lifetime map (τ_m) in HeLa cells showing weak autofluorescence where a) is the colour image corresponding to the two-photon fluorescence lifetime map of the τ_m, b) is the distribution curve of the τ_m where the colour represents the lifetime of a).

In order to be able to quantify compound fluorescence the background autofluorescence of cells had first to be assessed. The 810 nm control possessed the fluorescence lifetimes of 0.812 ns (τ_1 79 % 0.214 ns FWHM) and 3.140 ns (τ_2 21 %, 0.647 ns FWHM. The background fluorescence can be attributable to biological fluorophores; at 910 nm these include flavin adenine dinucleotide (FAD) and lipoamide dehydrogenase (LipDH), which also absorb at 810 nm.[15] Biological molecules such as reduced nicotinamide adenine dinucleotide (NADH) and nicotinamide adenine dinucleotide phosphate (NADPH) absorb 810 nm light, but not 910 nm, therefore contributing to the difference in autofluorescence intensity observed at the respective wavelengths.[15]

The full width at half maximum (FWHM), calculated from the lifetime distribution curve within the focal area was used to assess the error. The percentage of components τ_1 (major) and τ_2 (minor) in cells was from the respective amplitudes a_1 and a_2 calculated using SPCImage software, which models the data for each individual pixel to Equation 2.3, where F is fluorescence, a_0 is background and t is time:

$$F(t) = a_0 + a_1 e^{-t/\tau_1} + a_2 e^{-t/\tau_2}$$

7.1 Ligand precursor fluorescence lifetime investigation

For each bis(substituted) ligand precursor tested by Time-Correlated Single Photon Counting (TCSPC) two fluorescence lifetime components could be determined in DMSO at 10 mM, with the τ_1 accounting for at least 94 % in each case. The τ_1 values were very similar for compounds **II** and **3** when excited at 810 nm (0.226 ns with χ^2 of 1.88 and 0.183 ns with a χ^2 of 1.63). The excitation wavelength did not appear to have a significant effect on the fluorescence lifetime. Similarly the τ_1 of **4** was comparably shorter (0.020 ns at 810 nm and 0.021 ns at 910 nm), indicating no observable difference between the lifetimes when exciting at the respective wavelengths in DMSO solution.

The fluorescence lifetime of the compounds were obtained from the FLIM distribution curve in cancer cells (HeLa, MCF-7 and PC-3 cells). Standard conditions used were 1% DMSO 100 μM, with an incubation time of 20 minutes. The majority of cellular studies could be modelled to two fluorescence lifetime components, one of which was short (ca. 0.2 ns) with the other being long (ca. 2.5 ns). The solution data were within the FWHM, which represents the error for *in vitro* data and thus confirms their comparability.

There were some exceptions however, such as the long components of **3** in PC-3 cells (0.434 ns, 0.209 ns FWHM) and of **4** in HeLa and PC-3 cells at 910 nm (0.436 ns, 0.653 ns FWHM and 0.418 ns, 0.455 ns FWHM, respectively). Other notable exceptions were the major components observed for **II** and **3** when excited at 810 nm, with lifetimes of 0.903 ns and 0.868 ns, correspondingly. These are within the FWHM of the control, which suggests that most of the fluorescence observed at this wavelength for these two compounds is as a result of cellular autofluorescence. This could indicate the weakness of the fluorescence of the proligand or simply that the conformation of the compound within the cell and in turn the fluorescence lifetime has been significantly altered as opposed to in DMSO.

The minor components nevertheless, could be correlated to the ligand precursor, confirming the weak fluorescence of the compound in cells. The fluorescent lifetime measurements of the mono(substituted) ligands were similar to their bis(substituted) analogues, possessing in general a short, major component of the order of 0.4 ns and a long, minor component in the region of 3 ns. Unlike the bis-thiosemicarbazonato ligands, this was consistently observed in cells when excited at 910 nm. Different molecular orientations would result in a change in

fluorescent lifetime, which could suggest that the mono(substituted) proligand geometry was less variable than its bis(substituted) counterpart.[9] Interestingly, although consistency of data was observed in cells, the respective short, major component is slightly shorter (ca. 0.2 ns), which suggests that the complexity of the cellular environment does have impact on the lifetime. There appeared to be a more limited effect on the mono(substituted) than the bis(substituted) ligand precursor, which displayed greater variation of lifetime within cells. Notably, the fluorescence of the mono(substituted) ligand precursors was very weak and comparable to the bis(substituted) ligands.

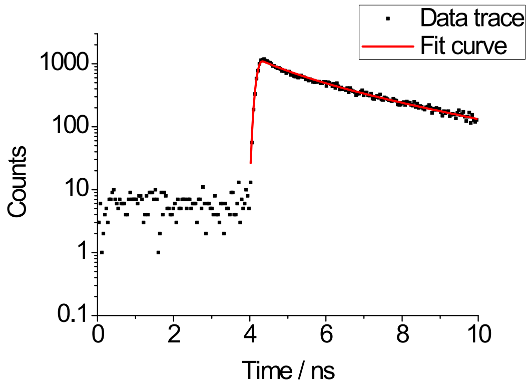

χ^2 = 1.18, τ_1 = 0.252 ns (43.3%), τ_2 = 0.858 ns (56.7%)

Figure S.33. Two-photon excitation Time-Correlated Single Photon Counting decay curve at 910 nm excitation for Ga(**3**) at 1 mM concentration in DMSO.

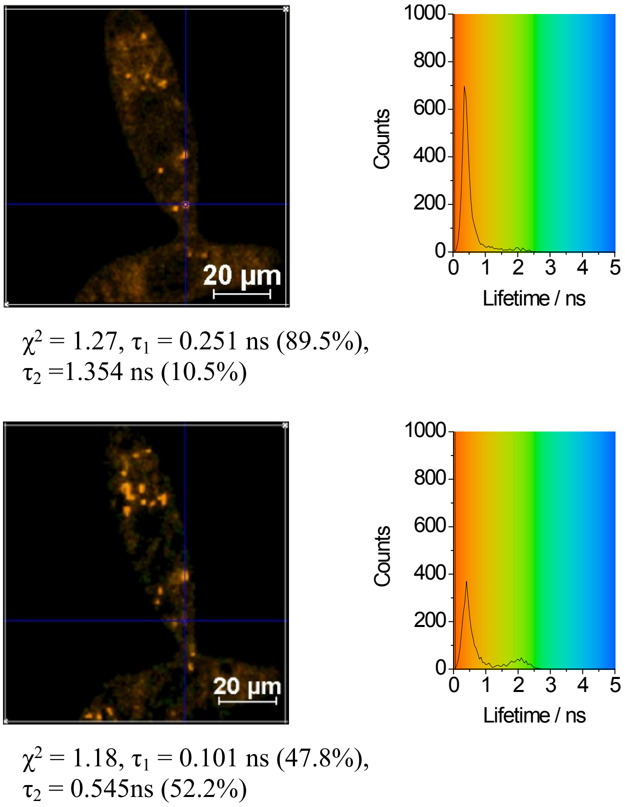

Figure S.34. Two-photon fluorescence lifetime imaging of complex Ga(**3**) at 50 µM, 0.5% DMSO, (a, b) before irradiation and (c, d) after irradiation for ca. 10 minutes. Where (a, c) are τ_m lifetime mapping images and (b, d) corresponding fluorescence lifetime distribution curves. Scalebar: 20 µm.

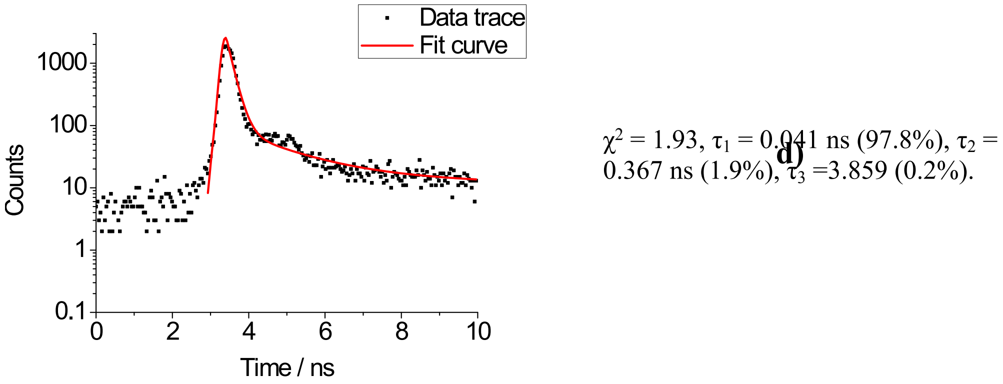

Figure S.35. Two-photon excitation Time-Correlated Single Photon Counting decay curve at 910 nm excitation for compound 3 at 10 mM concentration in DMSO. Notably, when a 1 mM DMSO solution of 3 was used, the resulting signal was not distinguishable from the background.

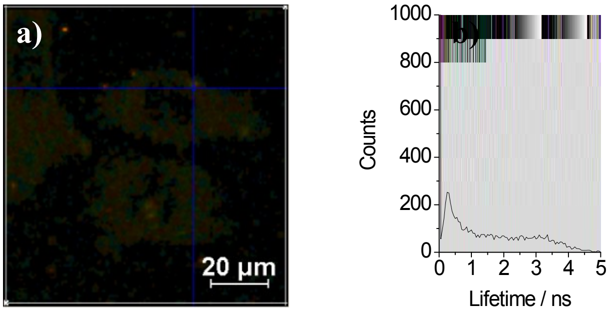

$\chi^2 = 1.24$, $\tau_1 = 1.556$ ns (100%)

Figure S.36. Two-photon fluorescence lifetime imaging of compound (3) at 100 µM, 1% DMSO, 20 minutes incubation time at 37°C. Where (a) is a τ_m lifetime mapping image and (b) the corresponding fluorescence lifetime distribution curve.

8. Kinetic stability testing in an aqueous environment

Gallium complex **Ga(4)** had an unexpected intensity increase with time up to 8 h. The compounds remained fluorescent at 24 h, but at a lower intensity than at 8 h. Preliminary assays with media in the absence of serum displayed a gradual decrease in fluorescence with time, which is consistent with the zinc complexes **Zn(4)** in serum and serum free media. The difference between these two assay types is protein content, indicating that the gallium compounds likely interact with the protein.

Whilst changes in the UV/Vis spectra for the gallium complex **Ga(4)** were observed over an incubation of 24 h in serum it is apparent that the product of the assay has not completely converted to free ligand **4**. UV/Vis spectroscopy studies on **Ga(4)** suggested that little decomposition occurs up to 3 h although some deteriation of the gallium species is evident after 18 h with LCMS analysis at this time suggesting the presence of both the parent chloride complex and some of the analogue in which the chloride ion is replaced by hydroxide.

This investigation utilised both UV-visible absorption (at 10 μM and at 100 μM) and fluorescence emission at 100 μM, with 1:1 DMSO:[assay agent] to limit precipitation. This allowed a % stability to be estimated *via* UV-Visible spectroscopy at 15 minutes and 24 hours as calculated by (100 % - percentage converted to free ligand) *(***Table S.3***)*. The first assays carried out in DMSO, water, PBS, 5% FCS EMEM and SFM EMEM enabled complex stability to be understood in media likely to be encountered in a cell biology experiment.

By UV-vis complex **Ga(4)** displayed encouraging results in DMSO, water and 5% FCS (88.2 %, 39.6 % and 79.1 % after 24 h respectively) (

Figure S.37 and **Figure S.38**). Complex **Ga(4)** was not stable during a 24 h incubation in PBS *(***Figure S.39***)* or in SFM and showed contrastingly high stability in SFM after 15 minutes (64.3 %) and limited remaining complex in PBS (10.9% after 15 minutes.) Moreover, **Ga(4)** demonstrated 75.3 % estimated stability in DMSO:water 1:1 after 15 minutes, but with a significant decrease in absorbance representing 39.6 % remaining complex after 24 h.

Table S.3. Summary of estimated complex remaining from UV-visible data at 1:1 DMSO:biologically relevant agent at 15 minutes and 24 h where Citric Acid, EDTA, L-Cys, L-His, L-Met and GSH were in MilliQ water.

Assay	Compound **Ga(4)**, 100 μM	
	15 min[a]	24 h[a]
DMSO	92.1	88.2
H_2O	75.3	39.6
PBS	10.9	NS
5% FCS, MEM	75.4	79.1
SFM, MEM	64.3	NS
Citric Acid	80.0	NS
EDTA	87.1	51.6
L-Cys	80.0	51.1
L-His	44.6	28.1
L-Met	58.1	45.1
GSH	52.8	7.7

For the fluorescence spectra, there was no significant change in intensity of complex **Ga(4)** in DMSO over a period of 24 h, which had showed a small decrease in fluorescence for the zinc complex **Zn(4)**. Subsequently complexes were tested and compared to the ligand precursor **4** in a 1:1 MilliQ water:DMSO solution over a period of 24 h. Under these conditions the gallium complex displayed a significant decrease in fluorescence after 1 day of incubation and a blue shift in fluorescence maxima of 13 nm after 24 h from 559.5 nm at 15 mins, indicative of some conversion to free ligand (a λ_{max} of 542 nm at 15 mins). The fluorescence intensity observed was sufficient to indicate the presence of some remaining gallium complex after 1 day, in agreement with UV-visible data.

Fluorescence data in PBS were significantly weaker than in water, in combination with a blue shift in absorbance is indicative of decomposition into bis-substituted free ligand. Most of the gallium complex appears to have become ligand within 15 minutes as confirmed by the estimated 10.9 % remaining complex calculated using UV-visible measurements and

possessed very similar λ_{max} of 537.5 nm for **Ga(4)** at 24 h and 538 nm for compound **4** at 15 mins.

The stability of the complexes was tested against Eagle's Minimum Essential Medium Serum Free Medium (SFM), which complexes are often incubated in for cell imaging. The fluorescence of each complex decreases over the time period studied at the λ_{max}, however complexes **Zn(4)** and **Ga(4)** interestingly show an increase in fluorescence at ca. 450 nm and 460 nm.

With λ_{max} of **Ga(4)** 536 and 537 nm at 15 minutes and 24 h respectively it is likely that a significant proportion of complex has rapidly become free ligand. An augmentation of absorbance and fluorescence was also observed in the case of complex **Ga(4)** after incubation of 24 h and retaining a band in the UV-visible spectrum at ca. 500 nm not observed in either free ligand **2** or **4**.

This is not surprising since FCS is known to increase background absorbance; interestingly, there was no fluorescence increase at ca. 450 nm or 460 nm as was observed in the absence of serum. This shows that presence of FCS is significant with regards to interaction of media and likely causes formation of the zinc and gallium complexes in an orientation or binding mode unlike that produced in EMEM alone.

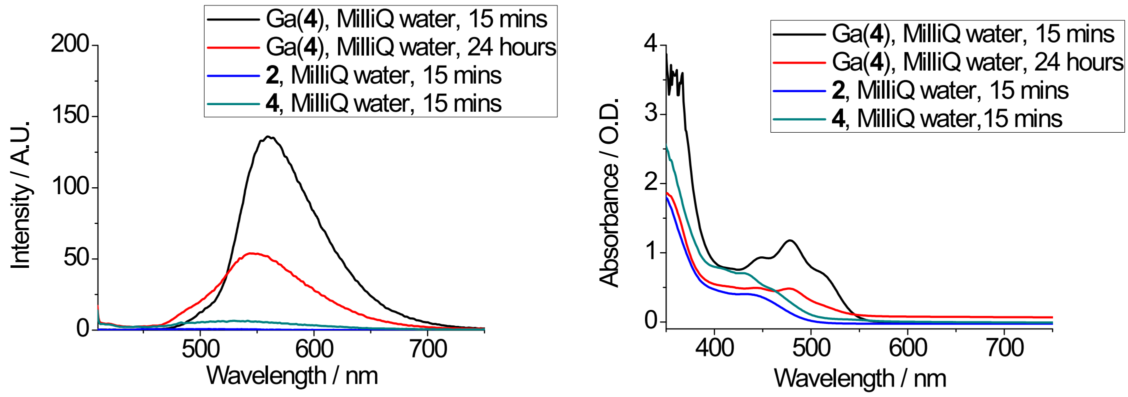

a) **b)**

Figure S.37. Kinetic stability assay in aqueous environment (50% DMSO, 50% water) for compound Ga(**4**) at a total concentration of100 μM, (a) by fluorescence spectroscopy and (b) by UV/Vis spectroscopy. In both cases, solutions were monitored at time intervals up to 24h at room temperature.

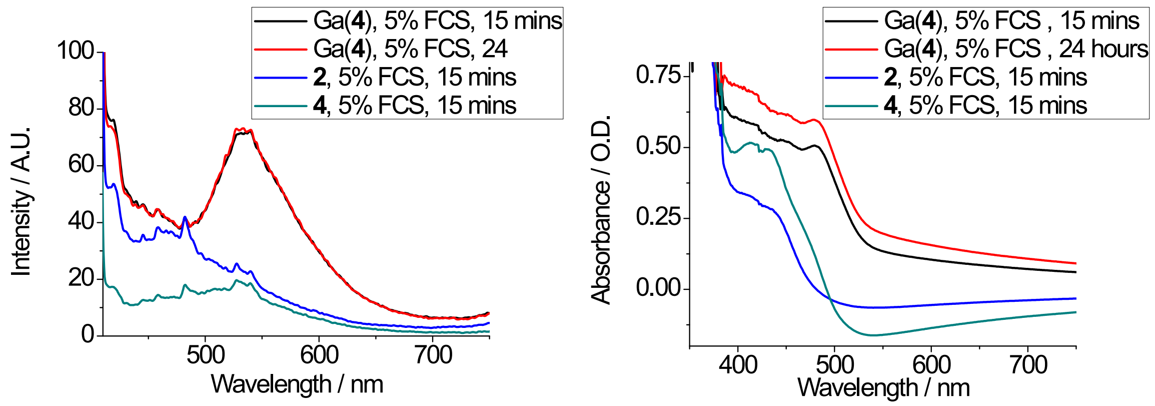

a) **b)**

Figure S.38. Kinetic stability assay in Foetal Calf Serum assay (50% DMSO, 45% EMEM, 5% FCS) for compound Ga(**4**) at a total concentration of 100 μM, (a) by fluorescence spectroscopy and (b) by UV/Vis spectroscopy. In both cases, solutions were monitored at time intervals up to 24 h at room temperature.

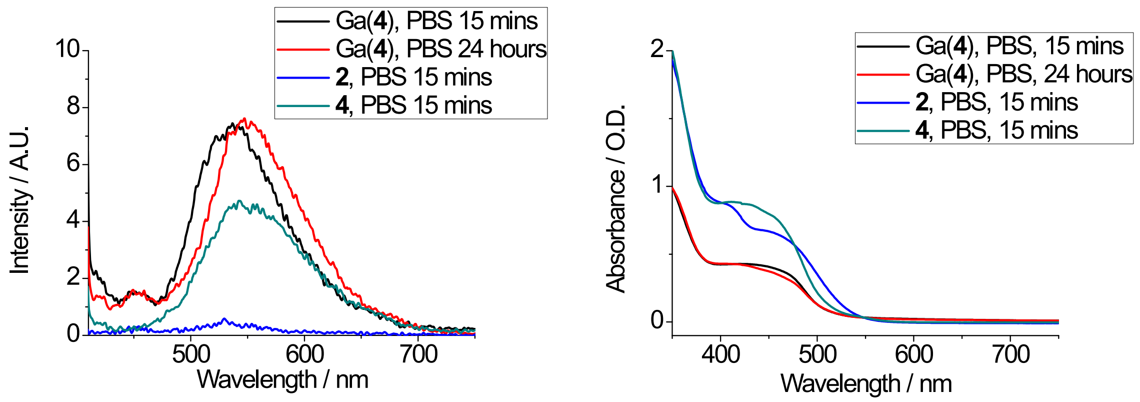

a) **b)**

Figure S.39. Kinetic stability assay in Phosphate Buffered Saline (50% DMSO, 50% PBS) for compound Ga(4) at a total concentration of 100 μM, (a) by fluorescence spectroscopy and (b) by UV-Vis spectroscopy. In both cases, solutions were monitored at time intervals up to 24 h at room temperature.

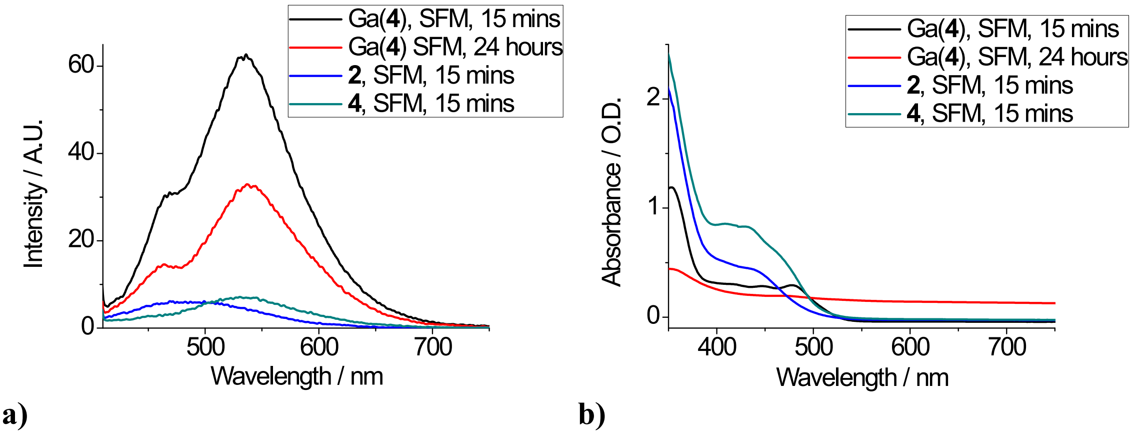

Figure S.40. Kinetic stability assay in serum free medium EMEM (50% DMSO, 50% EMEM) for compound Ga(**4**) at a total concentration of 100 μM, (a) by fluorescence spectroscopy and (b) by UV/Vis spectroscopy. In both cases, solutions were monitored at time intervals up to 24 h at room temperature.

9. Stability assays to estimate kinetic stability against common biologically-relevant agents

Biologically relevant agents were chosen to provide a better insight into the stability of these complexes,[16] as were introduced in. Complexes were tested in presence of 2 equivalents of citric acid, L-methionine, Glutathione (GSH), L-cysteine, L-histidine, EDTA and DFO. Complex **Ga(4)**, displayed less promising stability. In citric acid **Ga(4)** demonstrated significantly reduced fluorescence and absorbance in milliQ water whereby the complex appears stable at 15 minutes, at 24 h both fluorescence and UV-visible spectra were not characteristic of the allyl functionalised gallium complex. When incubated in PBS and citric acid rapid conversion to free ligand was observable due to similarity of the **Ga(4)** spectrum to distinctive absorption spectrum of **4** under the same conditions.

Conversely it is apparent that the **Ga(4)** UV-vis spectrum is not identical to **4** at 15 minutes, with significantly greater fluorescence at this timepoint showing that some gallium complex is remaining in PBS with citric acid. It is likely that **Ga(4)** has almost completely converted to **4** after 24 h of incubation attributable to the comparable spectra. In aqueous solutions of L-methionine, L-cysteine, L-histidine, or EDTA, **Ga(4)** displayed similar properties, each with the characteristic **Ga(4)** absorbance spectrum at 15 minutes followed by a significant decrease in absorbance at ca. 480 nm, but not the formation of data corresponding to that of the **4** free ligand.

This combined with the greater fluorescence of **Ga(4)** after 24 h incubation as compared with **4** at 15 minutes is indicative that not all of the complex has formed free ligand. The 24 h spectrum of **Ga(4)** in GSH is very similar to that of **4** at 15 minutes, displaying a very similar absorbance spectrum, indicating that most of **Ga(4)** has been converted to compound **4** under these conditions.

It can be observed that there is a much higher fluorescence intensity for **Ga(4)** at 24 h than for **4** at 15 minutes, indicating that although most complex may have converted to free ligand, it is likely that not all of the complex has. In contrast to the data acquired for L-cysteine, L-methionine and L-histidine, in which **Ga(4)** appeared much more stable.

In the presence of 2 eq. of DFO, it was observed that the UV-vis spectrum of Ga(**3**) was very similar to the initial one after 15 min compared to the one after 24 h where the band structure

changed drastically. On the other hand, the fluorescence spectra showed a reduction of fluorescence intensity to half of the initial value only after 15 min and after 24 h the fluorescence almost completely disappeared. When the complex Ga(**4**) was subjected to the same experiment with DFO it was observed that the intensity of the UV-vis spectrum was reduced after 15 min and after 24 h. The fluorescence intensity was reduced for Ga(**4**) to approximately half of the initial value after 15 min and totally disappeared after 24 h.

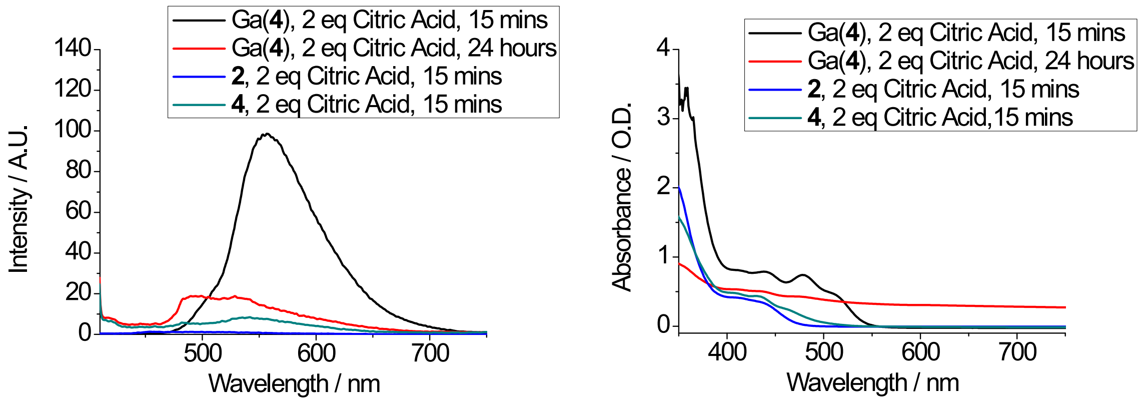

a) b)

Figure S.41. Kinetic stability assay in the presence of citric acid (50% DMSO, 50% water, 2 eq of citric acid with respect to the complex) for compound Ga(**4**) at a total concentration of 100 µM, in the mixture above, (a) by fluorescence spectroscopy and (b) by UV/Vis spectroscopy. In both cases, solutions were monitored at time intervals up to 24 h at room temperature.

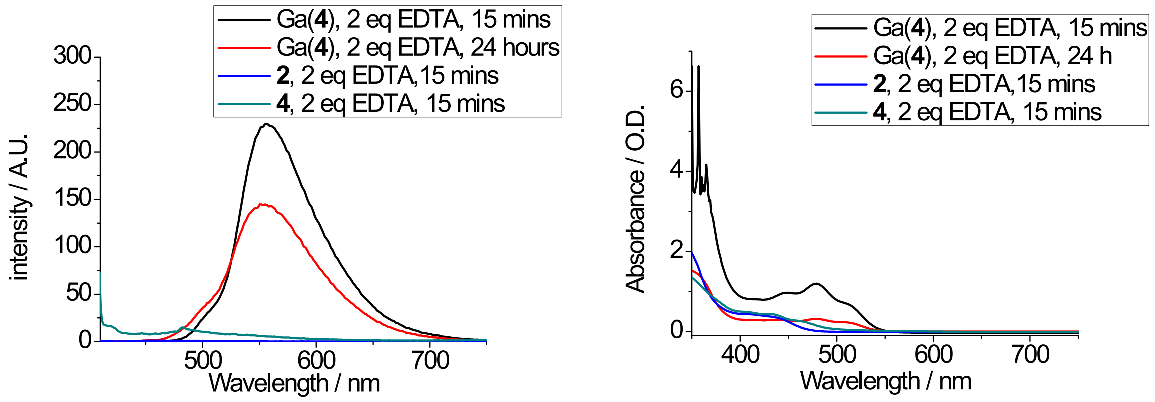

a) b)

Figure S.42. Kinetic stability assay in the presence of EDTA (50% DMSO, 50% water, 2 eq of EDTA with respect to the complex) for compound Ga(**4**) at a total concentration of 100 µM, by (a) fluorescence spectroscopy and (b) UV/Vis spectroscopy. In both cases, solutions were monitored at time intervals up to 24 h at room temperature.

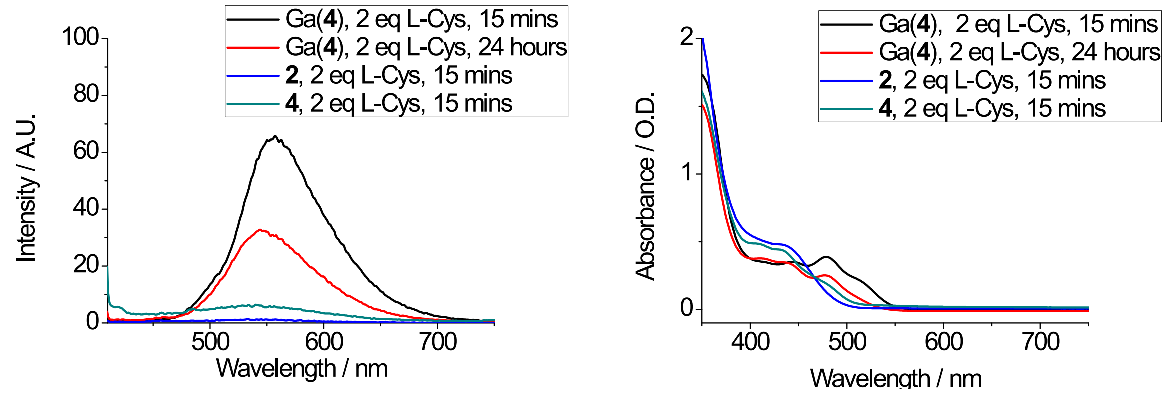

a) b)

Figure S.43. Kinetic stability assay in the presence of L-cysteine (50% DMSO, 50% water, 2 eq of L-cysteine with respect to the complex) for compound Ga(**4**) at a total concentration of 100 µM, (a) by fluorescence spectroscopy and (b) by UV/Vis spectroscopy. In both cases, solutions were monitored at time intervals up to 24 h at room temperature.

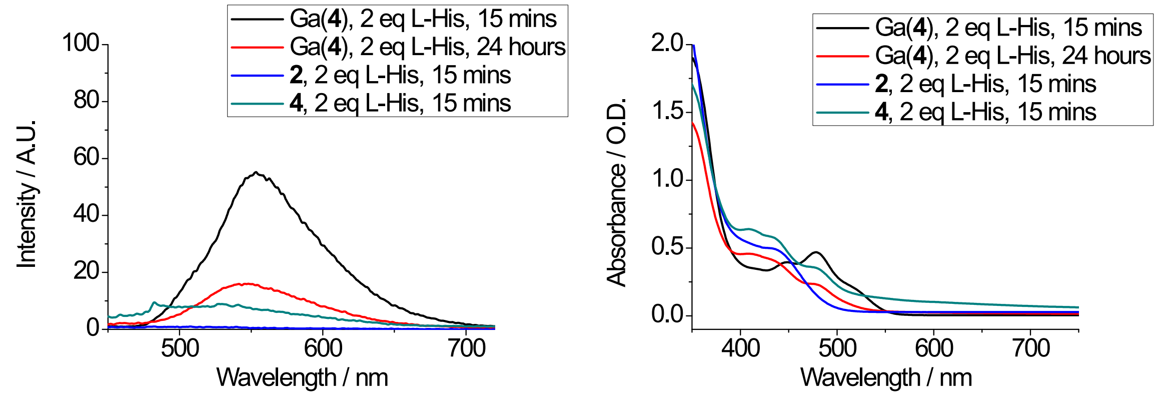

a) b)

Figure S.44. Kinetic stability assay in the presence of L-histidine (50% DMSO, 50% water, 2 eq of L- histidine with respect to the complex) for compound Ga(**4**) at a total concentration of 100 µM, (a) by fluorescence spectroscopy and (b) by UV/Vis spectroscopy. In both cases, spectroscopy of solutions above was monitored at time intervals up to 24 h at room temperature.

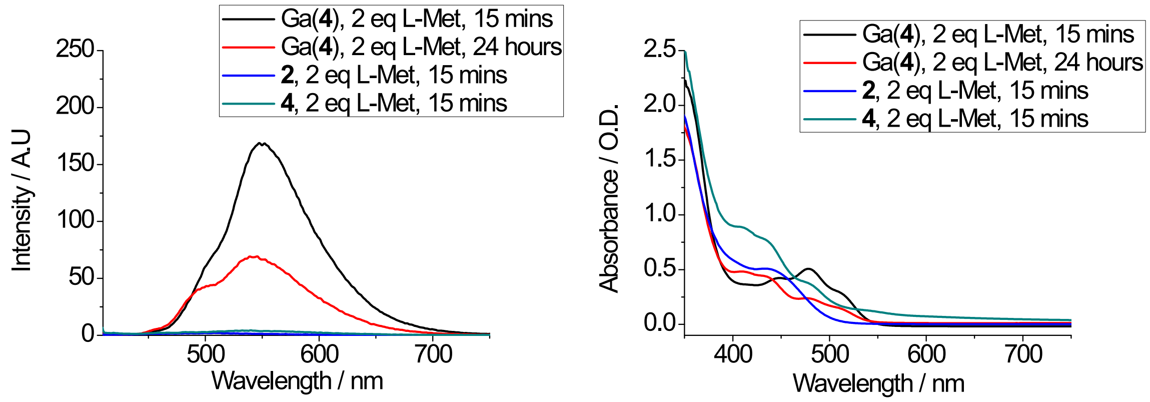

a) b)

Figure S.45. Kinetic stability assay in the presence of L-methionine (50% DMSO, 50% water, 2 eq of L-methionine with respect to the complex) for compound Ga(**4**) at a total concentration of 100 µM, (a) by fluorescence spectroscopy and (b) by UV/Vis spectroscopy. In both cases, solutions were monitored at time intervals up to 24 h at room temperature.

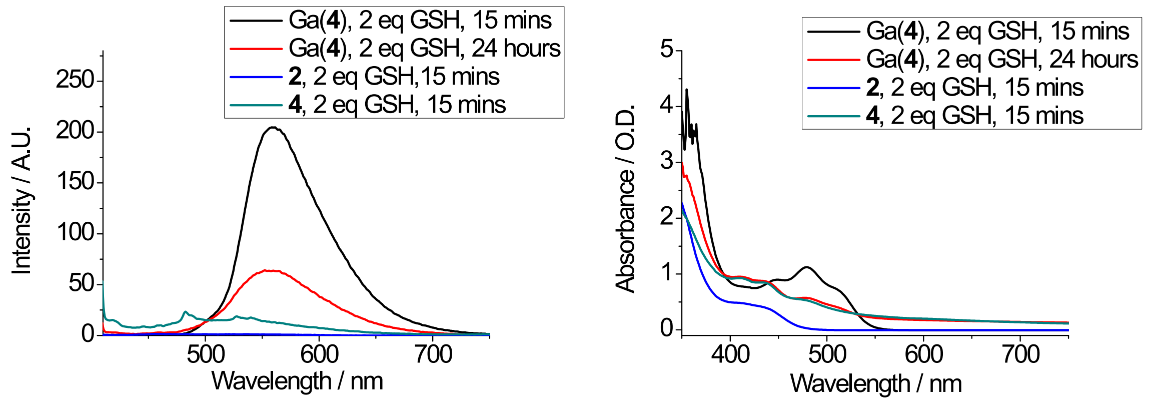

a) b)

Figure S.46. Kinetic stability assay in the presence of Glutathione (50% DMSO, 50% water, 2 eq of Glutathione with respect to the complex) for compound Ga(**4**) at a total concentration of 100 µM, (a) by fluorescence spectroscopy and (b) by UV/Vis spectroscopy. In both cases, solutions were monitored at time intervals up to 24 h at room temperature.

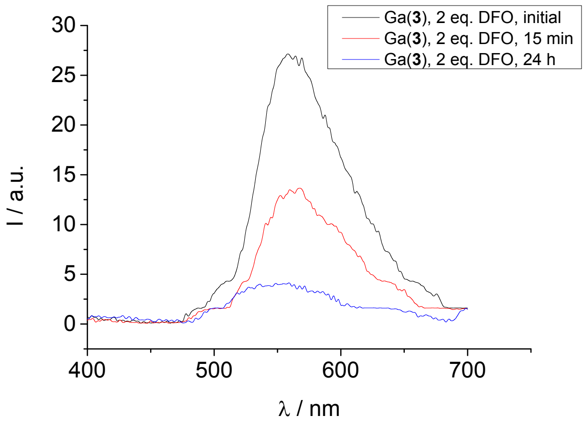

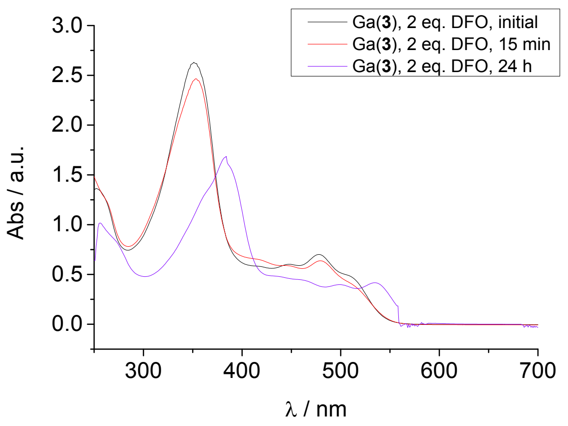

Figure S.47. Kinetic stability assay in the presence of 2 eq. of DFO (50% DMSO, 50% water) for compound Ga(**3**) at a total concentration of 100 μM, (a) by fluorescence spectroscopy and (b) by UV/Vis spectroscopy. In both cases, solutions were monitored at time intervals up to 24 h at room temperature.

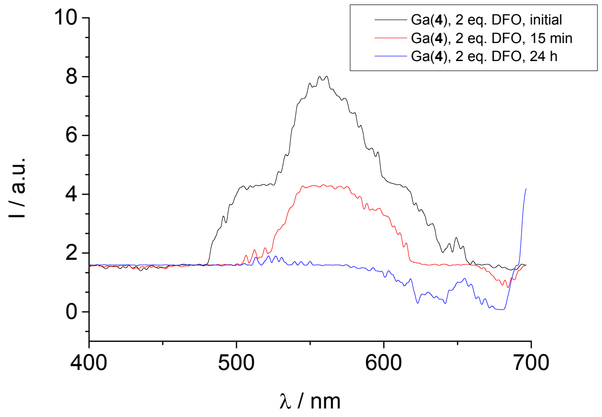

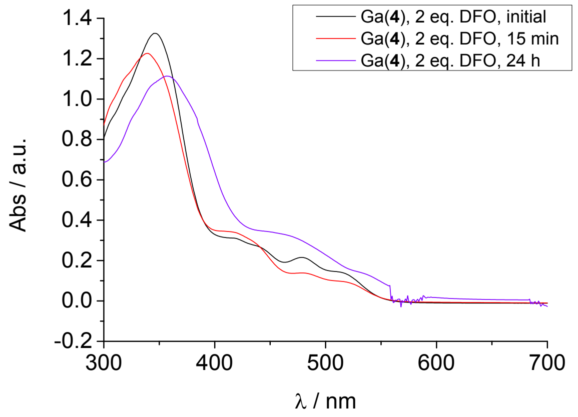

Figure S.48. Kinetic stability assay in the presence of 2 eq. of DFO (50% DMSO, 50% water) for compound Ga(**4**) at a total concentration of 100 μM, (a) by fluorescence spectroscopy and (b) by UV/Vis spectroscopy. In both cases, solutions were monitored at time intervals up to 24 h at room temperature.

9.1 pH study

The stability of complexes at biologically relevant pH is an important factor in this study. Cancer cells are slightly more acid than non-cancerous cells it is important that the molecular probes are stable at lower pH.[17] All fluorescent scans were carried out with an excitation wavelength of 400 nm and at a concentration of 100 μM, with a 1:1 DMSO:buffer solution. An initial preliminary study was carried out using fluorescence spectroscopy for the gallium complexes.

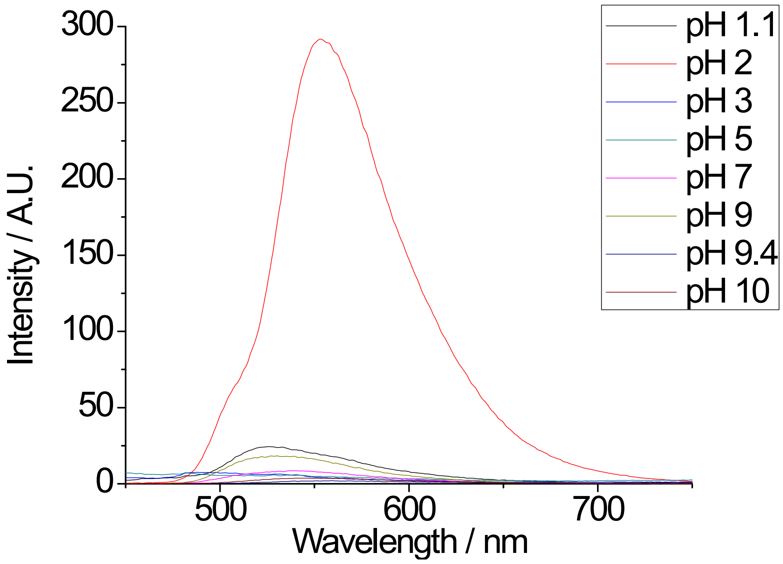

Figure S.49. Fluorescence spectroscopy buffer assay **Ga(3)** pH study (above). Where pH 2 >> pH 1.1 > pH 9 > pH 7 > pH 3 ≥ pH 5> pH 10 > pH 9.4

The gallium compounds all displayed maximum fluorescence at pH 2, with particularly intense fluorescence for **Ga(II)** and **Ga(3)** at this pH. It is interesting to notice that **Ga(II)** and **Ga(3)** are very fluorescent at pH 2, but very little fluorescent at pH 3. This could be explained by the nature of the species involved in the buffer. Buffer pH 2 contained HCl and KCl, whereas the pH 3, pH 5 and pH 7 buffers contained citric acid. Holland *et al.* have recently shown that citric acid can be used to remove zinc from zinc bis(thiosemicarbazonato) complexes, [18] which may have happened in the preliminary study, since dissociation of the metal centre would cause a decrease in fluorescence.

Table S.4. Summary of UV-visible data at 50: 50 DMSO: pH buffer at 15 minutes and 24 h, where NS = not stable.

	**Ga(4)**, 100 μM		
Buffer	Measured pH	% stability at 15 mins	% stability at 24 h
pH 1.1	1.6	68.6	NS
pH 2	2.5	85.5	53.9
pH 3	4.4	86.9	NS
pH 5	6.9	73.7	51.7
pH 7	9.4	5.0	NS
pH 9	9.35	6.2	NS
pH 10	12.7	6.8	NS

Therefore an intial study where citric acid was used was carried out (prior to the assays described above) followed by another investigation that did not include citric acid as part of the buffer systems, a means to monitor if the nature of the buffer had a significant effect. These gallium complexes are therefore very pH sensitive.

Furthermore all gallium complexes appeared most stable at either pH 2 or pH 3 after 15 minutes or less, which is also in agreement with data acquired where citric acid was a component of the buffer solutions. By UV-visible spectrometry compound **Ga(4)** seems to decompose completely after 24 hours when incubated in a pH 1.1 buffer, yet appears to remain intact at 15 minutes, as its spectrum has formed the shape characteristic of **4** in this time, fluorescence however appears to be fully quenched at 15 minutes. It is likely that stability of the gallium complex is poor at this pH. In pH buffers 2.0, 3.0 and 5.0 there is complex presence indicated by both absorption and emission spectra at 15 minutes, however a clear decrease in fluorescence intensity and absorbance including a blue shift for pH 3.0 and pH 5.0 buffers are indicative of instability of this compound at longer incubations.

As was observed with **Zn(4)**, **Ga(4)** spectra closely resembled those of compound **4** when incubated with a pH 7.0 buffer, however due to the high stability observed in the 5% FCS assay the apparent lack of stability could be explained by interaction with the components of the buffer rather than the pH itself.

Whilst spectroscopic (fluorescence and UV-vis) data acquired with the pH 7.0 and pH 9.0 buffers were similar, in both cases it was observable that conversion to **4** was not complete, indicating a small proportion of complex remaining. UV-visible and Fluorescence spectra of **Ga(4)** when incubated with a pH 10.0 buffer (measured pH with blank pH 12.7) displayed spectra more closely resembling the free ligand **4**, indicating that the stability was poorer under more alkaline conditions. The range of good stability of this complex when considering the measured pH with the blank is pH 2.5 to pH 6.9, however under no conditions tested using the pH buffers was complete conversion to **4** after 15 minutes, indicating that short-term experiments in biological environments are valid with this compound, especially with the stability observed after 24 h in 99% FCS for this complex, with a blank pH of 7.35. In summary the spectra acquired support the legitimacy carrying out cell uptake experiments, where incubations would be less than 20 minutes, whereby the complex would most likely intact under these biomimetic conditions.

10. Preliminary *In vivo* imaging tests by microPET

Normoxic PC-3 xenografts were grown on the right shoulder of nude mice in Memorial Sloan-Kettering Cancer Center, New York. MicroPET was carried out for ^{68}Ga complex Ga(**3**), good renal clearance was observed, indicating suitability of these probes. Furthermore, no tumour uptake under these normoxic conditions was observed for all cases, with some limited uptake in the lungs, liver and spleen and clear bladder localisation/excretion.

This represents a promising result for probes designed, signifying that it would be beneficial for an *in vitro* hypoxia uptake focused future study to be carried out to reveal the exact biodistribution.

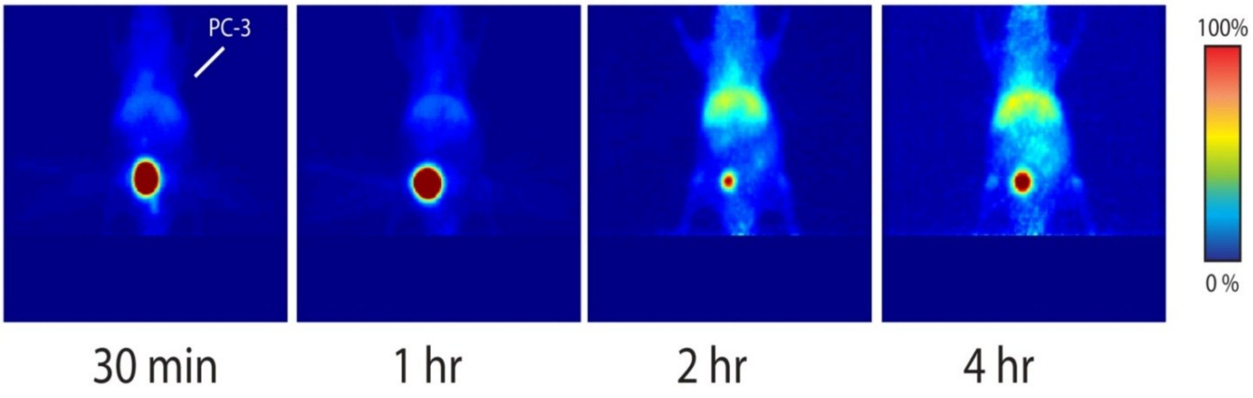

a) **b)** **c)** **d)**

Figure S.50. Preliminary MicroPET experiments showing images of in nude mice, for M = 68-Ga, R = Et, where a, b, c and d are images recorded at timepoints 30 mins, 1h, 2h and 4h respectively post-injection.

11. Hypoxia selectivity testing

Compound **Ga(4)** was incubated in cells at 50 µM, 4% DMSO, with normoxic conditions of 20.7% O_2 and 5% CO_2 at 37 °C, with hypoxic samples pre-incubated for 20 minutes at 1%

O_2, 5% CO_2 at 37 °C before complex addition. Following addition of the compound cells were incubated for a further 20 minutes and subsequently washed three times with PBS before being returned to serum free media and imaged immediately.

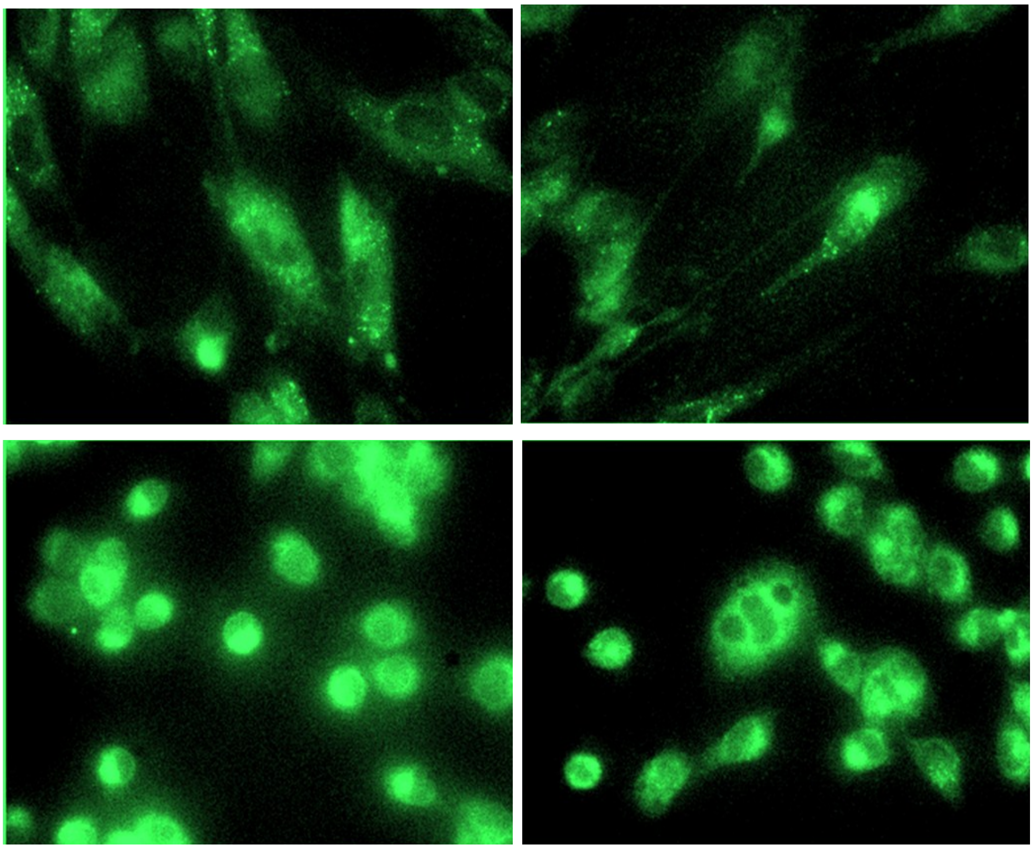

Figure S.51. Confocal micrographs of **Ga(4)** in FEK-4 (top) and EMT6 (bottom) cells under normoxic (left) and hypoxic (right) conditions.

Using confocal imaging the compound preference for hypoxia was not easily deducible, however, flow cytometry and radioactive cell uptake studies, provided quantitative means to assess the selectivity (conditions used are within the experimental section).

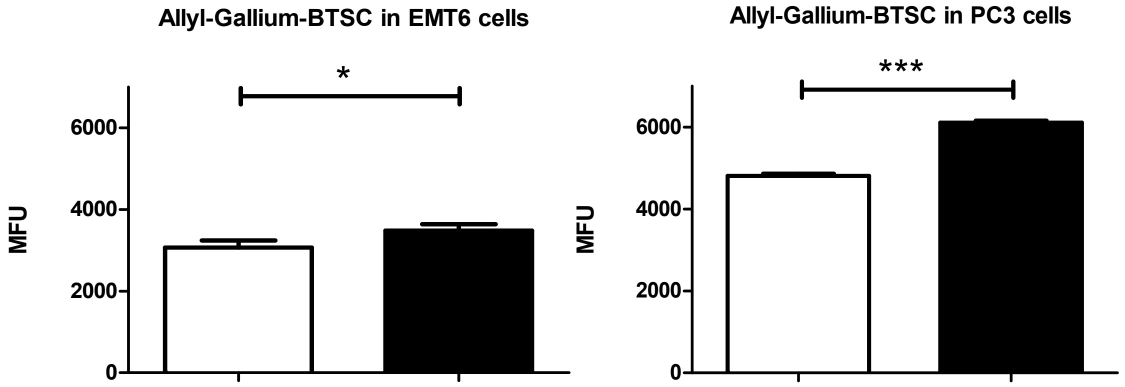

Figure S.52. Flow cytometry studies under normoxia and hypoxia of Ga(4) (a) in EMT6 and (b) in PC-3 cells under hypoxic (white bars) and normoxic (black bars) conditions. Hypoxia in cells was induced for 20 minutes prior to addition of compound in 1% O2 and for a further 20 minutes in the same conditions post addition of the compound. Data shown are median fluorescence intensities (MFU) +/- SD (*n* =2 or 1 experiments, samples run in triplicates). MFU values are in arbitrary units (a.u.) and were standardized to green autofluorescence levels. (Exc, λ =350; Em, λ = 475 nm, *x*-axis). Staining is significantly lower in cells under hypoxia (*P<0.05 and ***P<0.001 for EMT6 and PC3 cells respectively).

Median fluorescence intensities (MFU) obtained using flow cytometry under hypoxic conditions were lower than those of the normoxic cells (12% lower for EMT6 (*P<0.05) and 21 % lower in PC-3 cells (***P<0.001). Although this decrease in fluorescence may be understood as lower uptake, it is more likely that incubation under hypoxic conditions causes reduction of the complex, which would result in demetallation and therefore loss of fluorescence. It consequently is difficult using this technique alone to determine if the lower fluorescence is due to less uptake, conversion to ligand and lower uptake or conversion to ligand and increased uptake.

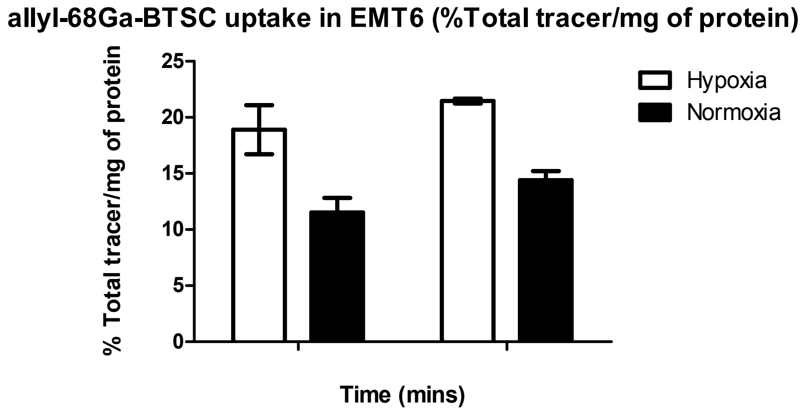

Figure S.53. Radioactive cell uptake studies under normoxia and hypoxia of Ga(**4**) in EMT6 cells.

To investigate this issue a preliminary radioactive cell uptake experiment was carried out, showing that ^{68}Ga uptake was greater in hypoxic cells than normoxic cells (64% and 49% higher at 30 and 60 minutes respectively). This therefore indicates that the most likely cause of the reduction in fluorescence is due to the combination of increase in uptake as well as conversion to free ligand, signifying that the gallium complexes possesses selectivity for cells under hypoxic conditions. Figure below shows relatively high uptake of the allyl-^{68}Ga-BTSC compound in the EMT6 cells, especially when compared to uptake with ^{68}GaCl3 (Figure 2). Total activity counted (% total tracer/mg of protein) is higher under hypoxia than normoxia (need at least another experiment to do appropriate statistics and test significance). This difference observed is highest at 2 hours (2-fold increase in uptake under hypoxia).

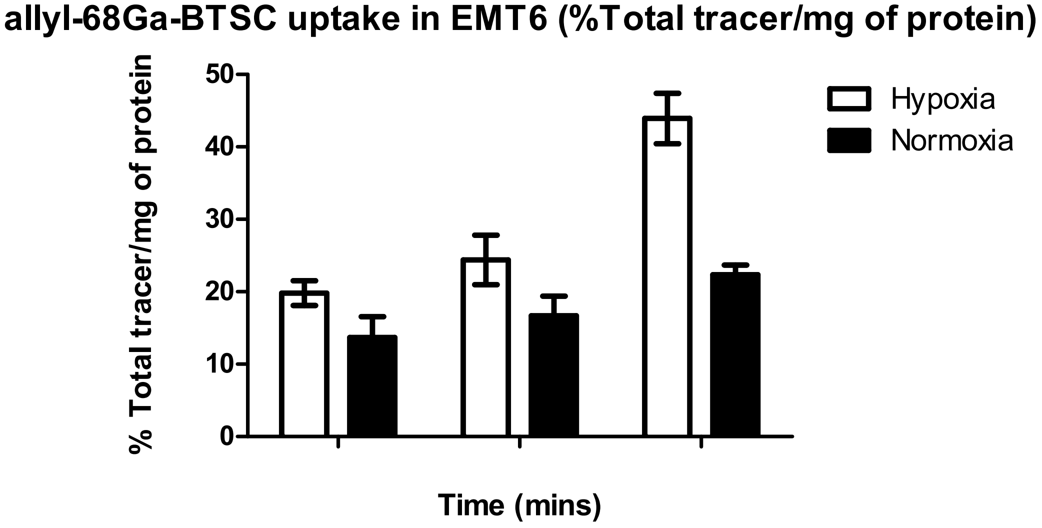

Figure S.54. allyl-^{68}Ga-BTSC uptake in EMT6 cells. Uptake of allyl-^{68}Ga-BTSC in EMT6 cells under hypoxic (white bars) or normoxic conditions (black bars) was measured at 30, 60 and 120 minutes post addition of tracer and is expressed as % of total tracer/mg of protein. Hypoxia in cells was induced for 20 minutes prior to addition of the tracer in 1% O_2 and cells were maintained under the same conditions for the remainder of the time course. Higher uptake is observed under hypoxic conditions. Data represents mean values +/-SD (n=2 separate experiments, samples were run as duplicates).

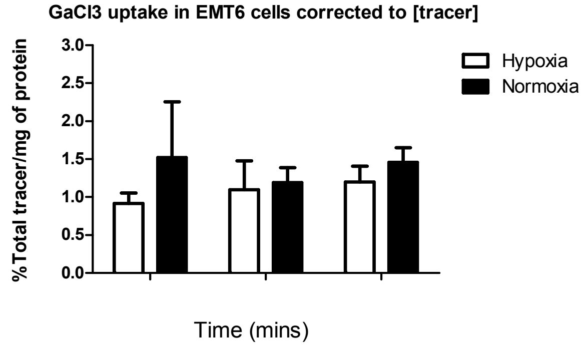

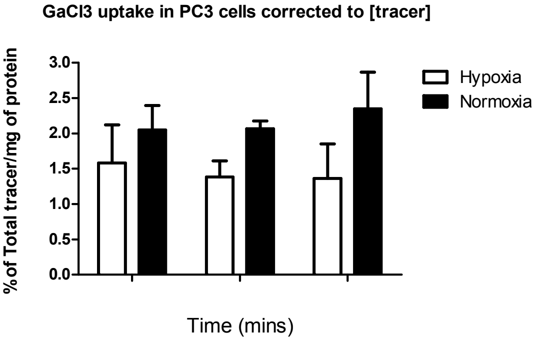

Figure S.55. $^{68}GaCl_3$ uptake in EMT6 and PC3 cells. Uptake of $GaCl_3$ in cells under hypoxic (white bars) or normoxic conditions (black bars) was measured at 30, 60 and 90 minutes post addition of tracer and is expressed as % of total tracer/mg of protein. Hypoxia in cells was induced for 20 minutes prior to addition of $^{68}GaCl_3$ in 1% O_2 and cells were maintained under the same for the remainder of the time course. There is no significant difference in the uptake of $^{68}GaCl_3$ in hypoxic vs. normoxic cells. Data represents mean values +/-SD (n=2, samples were run as duplicates).

12. MTT assays

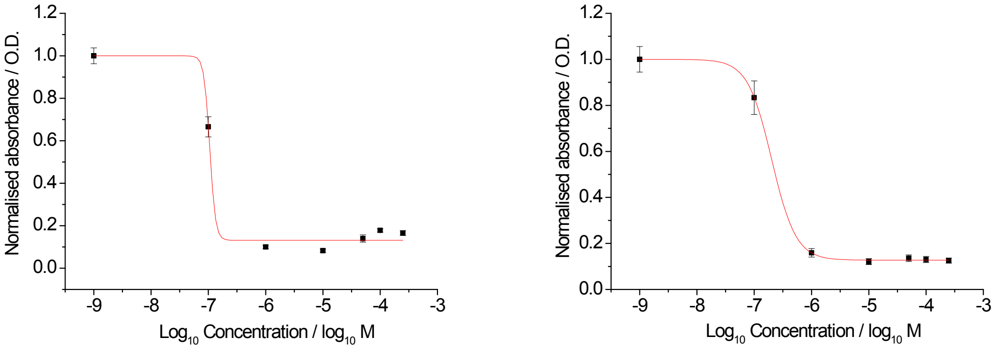

a) b) b)

Figure S.56. Scatter graph representing MI50 in HeLa cells a) of **I** and b) of **2**

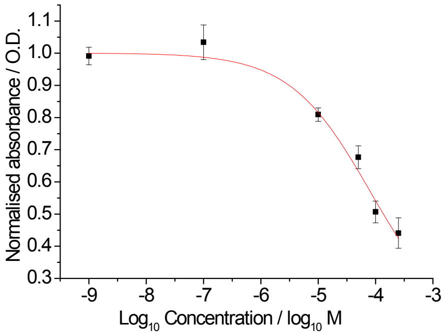

Figure S.57. Scatter graph representing MI_{50} in HeLa cells of **3**

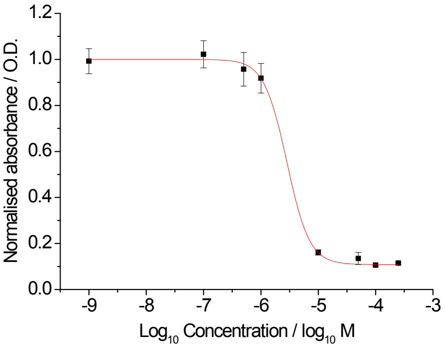

Figure S.58. Scatter graph representing MI_{50} in HeLa cells of Zn(**3**)

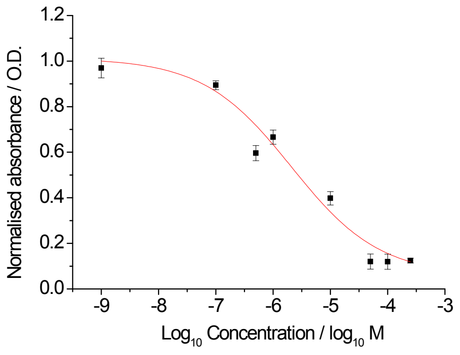

a)

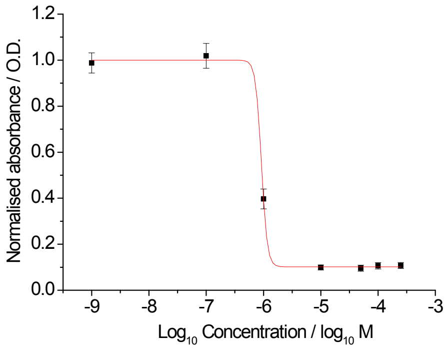

b)

Figure S.59. Scatter graph representing MI_{50} in HeLa cells of a) Ga(**3**) and b) Ga(**4**)

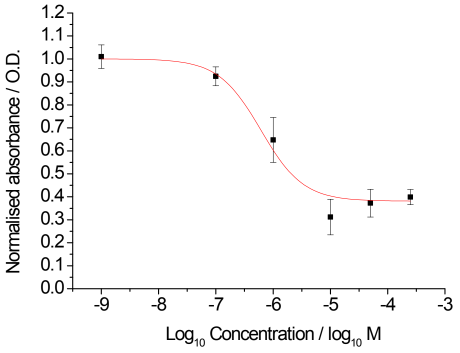

a)

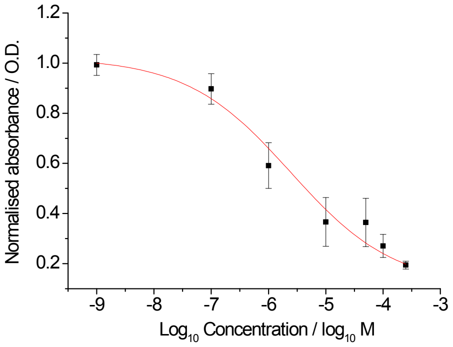

b)

Figure S.60.Scatter graph representing MI_{50} in FEK-4 cells a) of **I** b) of **2**

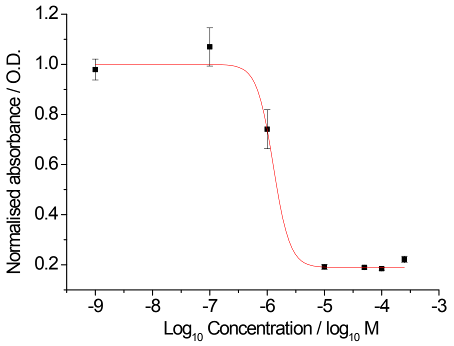

b)

Figure S.61. Scatter graph representing MI_{50} in FEK-4 cells b) of Ga(**4**)

13. Cyclic voltammetry experiment of bis(thiosemicarbazonato) complexes and ligands

Cyclic voltammetry measurements were carried out in degassed dimethylformamide using glassy carbon as working electrode, platinum as supporting electrode and a silver wire as pseudo reference electrode. Compounds **3**, **4** and complexes Zn(**3**), Zn(**4**), Ga(**3**) and Ga(**4**) were present at a 1 mM concentration, ferrocene was used as a reference at a 1 mM concentratrion and tetrabutylammonium tetrafluoroborate was added as supporting electrolyte in a 0.1 M concentration.

Initial and final potential was -1.6 V and scan rate was set at 100 mV/s. Five cyclic voltammograms were acquired for each compound.

Table S.5. Anodic (Epa) and cathodic (Epc) peak potentials, half-wave potential ($E_{1/2}$) and anodic and cathodic peak separation (Δ_{Ep}) for ferrocene. All parameters expressed in Volts.

	**Epa**	**Epc**	**$E_{1/2}$**	**Δ_{Ep}**
**Fc**	0.42	0.55	0.48	0.13

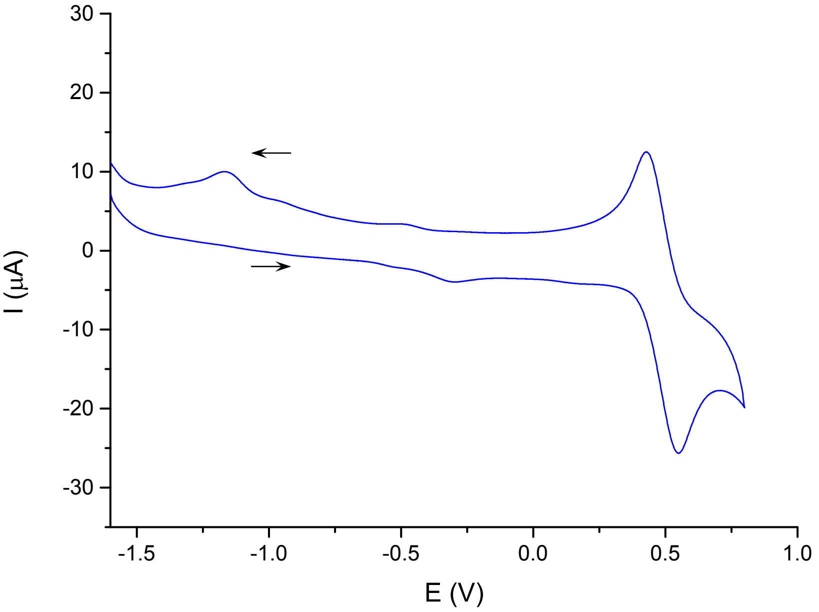

Figure S.62. Cyclic voltammogram of compound **3** and ferrocene.

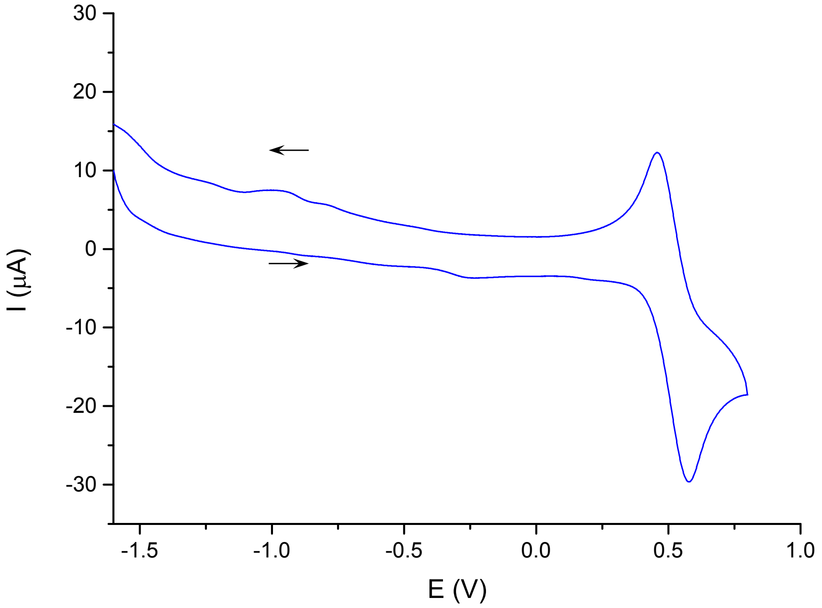

Figure S.63. Cyclic voltammogram of compound **4** and ferrocene.

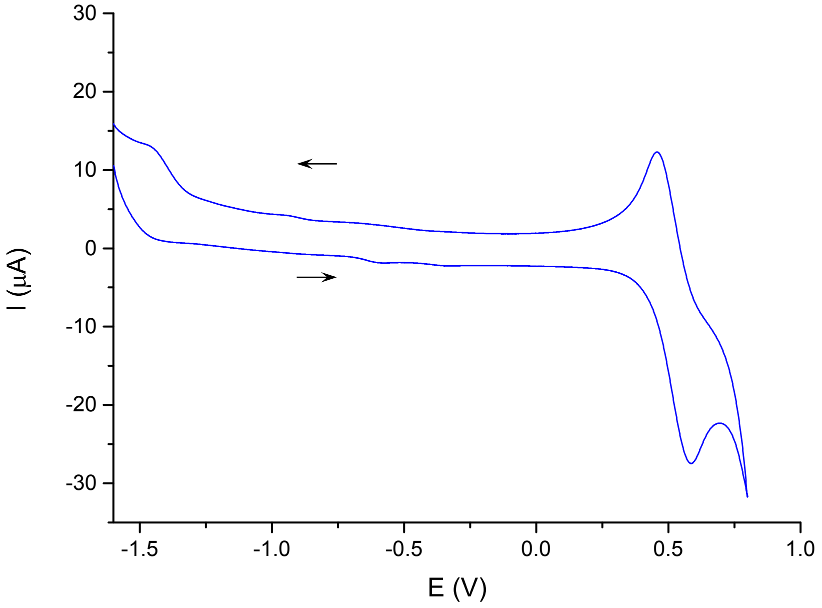

Figure S.64. Cyclic voltammogram of complex Zn(**3**) and ferrocene.

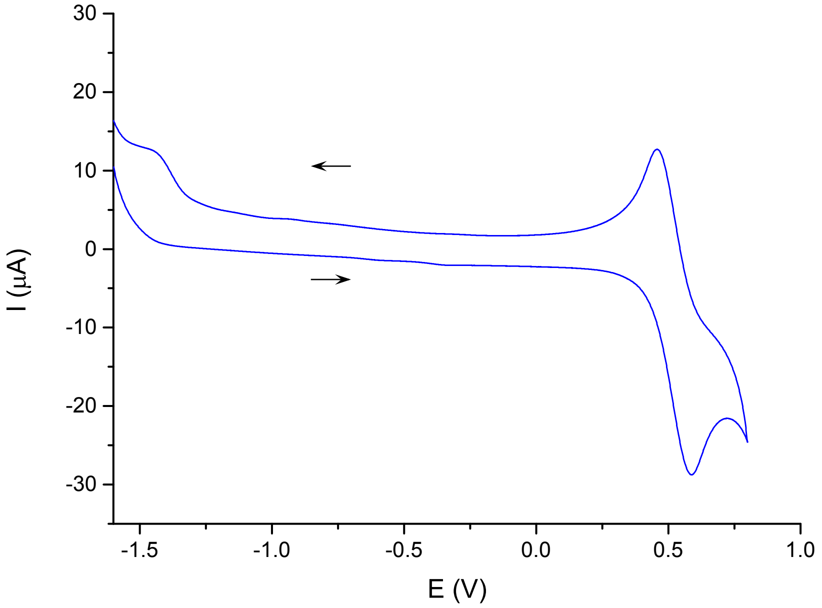

Figure S.65. Cyclic voltammogram of complex Zn(**4**) and ferrocene.

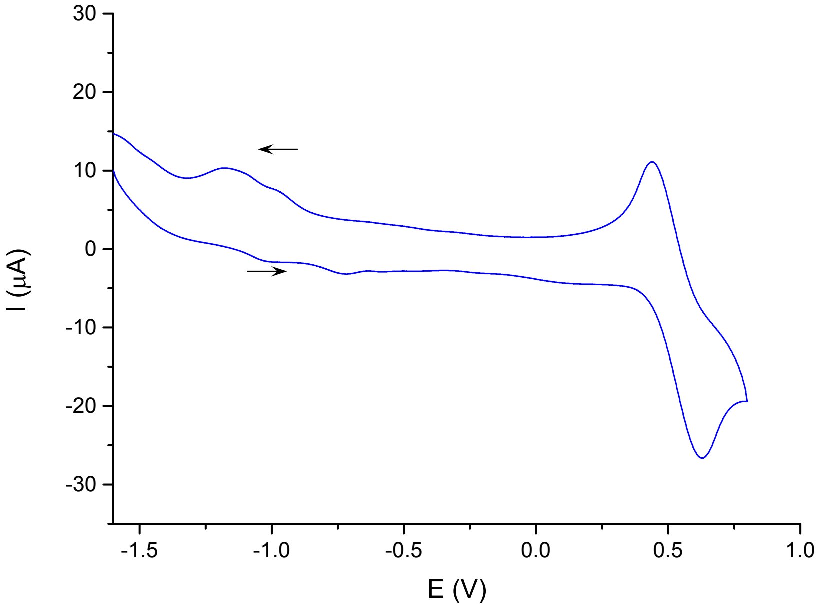

Figure S.66. Cyclic voltammogram of complex Ga(**3**) and ferrocene.

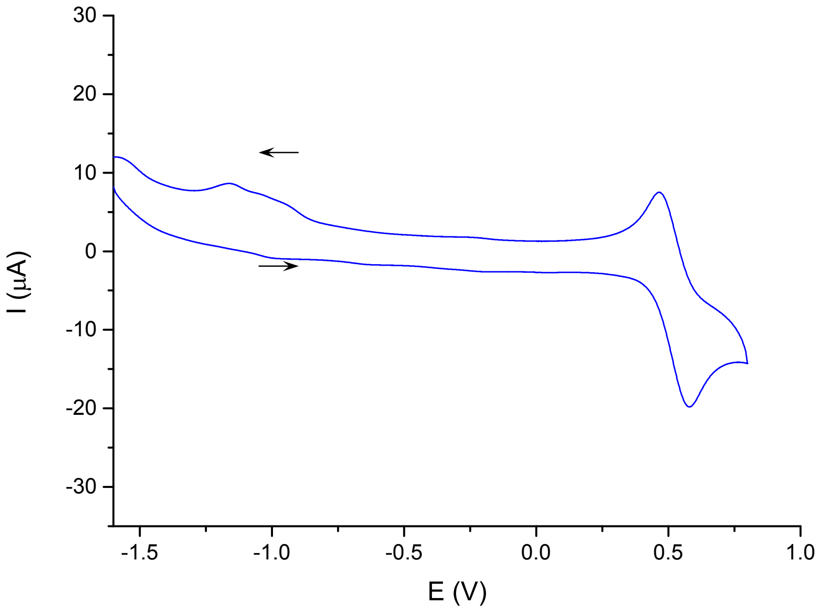

Figure S.67. Cyclic voltammogram of complex Ga(**4**) and ferrocene.

14. References

1. Z. Otwinowski and W. Minor, *Macromolecular Crystallography, Pt A*, 1997, **276**.
2. A. Altomare, M. C. Burla, M. Camalli, G. L. Cascarano, C. Giacovazzo, A. Guagliardi, A. G. G. Moliterni, G. Polidori and R. Spagna, *J. Appl. Crystallogr.*, 1999, **32**, 115-119.
3. G. M. Sheldrick, *Acta Crystallographica Section A*, 2008, **64**, 112-122.
4. C. B. Huebschle, G. M. Sheldrick and B. Dittrich, *J. Appl. Crystallogr.*, 2011, **44**, 1281-1284.
5. P. Vandersluis and A. L. Spek, *Acta Crystallographica Section A*, 1990, **46**, 194-201.
6. C. Mohan, *Buffers: A guide for the preparation and use of buffers in biological systems*, 1997.
7. J. R. Lakowicz, *Principles of fluorescence spectroscopy*, 4th Printing edn., Springer, 2006.
8. P. G. Bush, D. L. Wokosin and A. C. Hall, *Front. Biosci.*, 2007, **12**, 2646-2657.
9. W. Becker, *J. Microsc.*, 2012, **247**, 119-136.
10. N. I. Cade, G. Fruhwirth, S. J. Archibald, T. Ng and D. Richards, *Biophys. J.*, 2010, **98**, 2752-2757.
11. S. Despa, P. Steels and M. Ameloot, *Anal. Biochem.*, 2000, **280**, 227-241.
12. J. R. Lakowicz and H. Szmacinski, *Journal of Fluorescence*, 1996, vol. 6, pp. 83-95.
13. S. W. Botchway, A. W. Parker, R. H. Bisby and A. G. Crisostomo, *Microsc. Res. Tech.*, 2008, **71**, 267-273.
14. R. L. Arrowsmith, P. A. Waghorn, M. W. Jones, A. Bauman, S. K. Brayshaw, Z. Hu, G. Kociok-Koehn, T. L. Mindt, R. M. Tyrrell, S. W. Botchway, J. R. Dilworth and S. I. Pascu, *Dalton Trans.*, 2011, **40**, 6238-6252.
15. S. H. Huang, A. A. Heikal and W. W. Webb, *Biophys. J.*, 2002, **82**, 2811-2825.
16. P. J. Barnard, S. R. Bayly, J. P. Holland, J. R. Dilworth and P. A. Waghorn, *Quarterly Journal of Nuclear Medicine and Molecular Imaging*, 2008, **52**, 235-244.
17. J. J. Steffan, J. L. Snider, O. Skalli, T. Welbourne and J. A. Cardelli, *Traffic*, 2009, **10**, 737-753.
18. J. P. Holland, J. A. Hickin, E. Grenville-Mathers, T. Nguyen and J. M. Peach, *J. Chem. Res.*, 2008, 702-703.
